# Enhancing the Discovery of Bioactive Secondary Metabolites From Fungal Endophytes Using Chemical Elicitation and Variation of Fermentation Media

**DOI:** 10.3389/fmicb.2022.898976

**Published:** 2022-06-06

**Authors:** Martin Muthee Gakuubi, Kuan Chieh Ching, Madhaiyan Munusamy, Mario Wibowo, Zhao-Xun Liang, Yoganathan Kanagasundaram, Siew Bee Ng

**Affiliations:** ^1^Singapore Institute of Food and Biotechnology Innovation (SIFBI), Agency for Science, Technology and Research (A*STAR), Singapore, Singapore; ^2^School of Biological Sciences, Nanyang Technological University, Singapore, Singapore

**Keywords:** bioactive compounds, drug discovery, diversity, endophytic fungi, secondary metabolites

## Abstract

Endophytic microorganisms are an important source of bioactive secondary metabolites. In this study, fungal endophytes obtained from A*STAR’s Natural Product Library (NPL) and previously isolated from different habitats of Singapore were investigated for their diversity, antimicrobial, and cytotoxic activities. A total of 222 fungal strains were identified on the basis of sequence analysis of ITS region of the rDNA gene. The identified fungal strains belong to 59 genera distributed in 20 orders. Majority of the identified strains (99%; 219 strains) belong to the phylum *Ascomycota*, while two strains belonged to the phylum *Basidiomycota*, and only one strain was from *Mucoromycota* phylum. The most dominant genus was *Colletotrichum* accounting for 27% of all the identified strains. Chemical elicitation using 5-azacytidine and suberoylanilide hydroxamic acid (SAHA) and variation of fermentation media resulted in the discovery of more bioactive strains. Bioassay-guided isolation and structure elucidation of active constituents from three prioritized fungal strains: *Lophiotrema* sp. F6932, *Muyocopron laterale* F5912, and *Colletotrichum tropicicola* F10154, led to the isolation of a known compound; palmarumycin C_8_ and five novel compounds; palmarumycin CP_30_, muyocopronol A-C and tropicicolide. Tropicicolide displayed the strongest antifungal activity against *Aspergillus fumigatus* with an IC_50_ value of 1.8 μg/ml but with a weaker activity against the *Candida albicans* presenting an IC_50_ of 7.1 μg/ml. Palmarumycin C_8_ revealed the best antiproliferative activity with IC_50_ values of 1.1 and 2.1 μg/ml against MIA PaCa-2 and PANC-1 cells, respectively.

## Introduction

Microbial secondary metabolites remain a major source of bioactive molecules with a broad range of applications in the pharmaceutical, food, cosmetic, and agrochemical industries (Singh et al., 2017). Microbial-originated drug discovery has gained new impetus in recent years as a result of the tremendous advancements that have been witnessed in genomics especially high-throughput sequencing, bioinformatics in addition to the increasing incorporation of synthetic biology in natural product research (Bachmann et al., 2014; Keller, 2019). Following decades of exploration of bioactives from the traditional microbial sources such as soil, and with the continued diminishing hit rates from these microbial sources, natural product researchers have had to search for alternative sources of microbes for lead molecules in drug discovery (An et al., 2020). Some of the most promising group of microbes in this regard are those derived from the less studied and specialized niches such of endophytic microorganisms.

Fungal endophytes represent a relatively underexplored resource with a huge potential for the discovery of molecules with greater chemical novelty and interesting bioactivities (Zheng et al., 2016; Strobel, 2018; Gakuubi et al., 2021). These microbes occupy a unique ecological niche that is characterized by a wide range of interactions established over the years through their co-existence with host plants (Kusari et al., 2012; Gupta and Shukla, 2020). Endophytic fungi are among the most diverse group of fungi and have been isolated from practically all types of plants and plant tissues (Chowdhary et al., 2012; Yu et al., 2018). Indeed, every plant studied up till now has been found to harbor at least one fungal endophyte with some plants hosting hundreds of fungal strains (Faeth and Fagan, 2002). Already, a plethora of valuable molecules including antibiotics (Deshmukh et al., 2014; Martinez-Klimova et al., 2017), antifungal compounds (Deshmukh et al., 2018; Sinha et al., 2019), antimycobacterial compounds (Alvin et al., 2014), anticancer compounds (Kharwar et al., 2011; Chen et al., 2014; Uzma et al., 2018), antioxidants molecules (Toghueo and Boyom, 2019), and enzymes with diverse applications (Corrêa et al., 2014; Khan et al., 2017; Mishra et al., 2019) have been isolated from these gifted microbial factories.

Bioprospecting of fungal endophytes from diverse geographies and habitats enhances the likelihood of discovering promising strains capable of biosynthesis of novel molecules with valuable industrial and biotechnological applications. With a typical tropical climate, Singapore is home to a diverse collection of native and exotic plant species that may as well harbor an equally diverse collection of fungal endophytes including potentially new undescribed strains. The objective of this work therefore was to evaluate the diversity and bioactive potential of fungal endophytes from the A*STAR Natural Product Library (NPL). Aptly described as among the largest natural organism libraries in the world, the library which is housed at the Singapore Institute of Food and Biotechnology Innovation (SIFBI) boasts a collection of more than 54,000 and 58,000 fungi and actinomycetes, respectively, in addition to thousands of eubacteria and plant specimens (Ng et al., 2018).

## Materials and Methods

### Endophytic Fungal Strains

**Figure 1:**
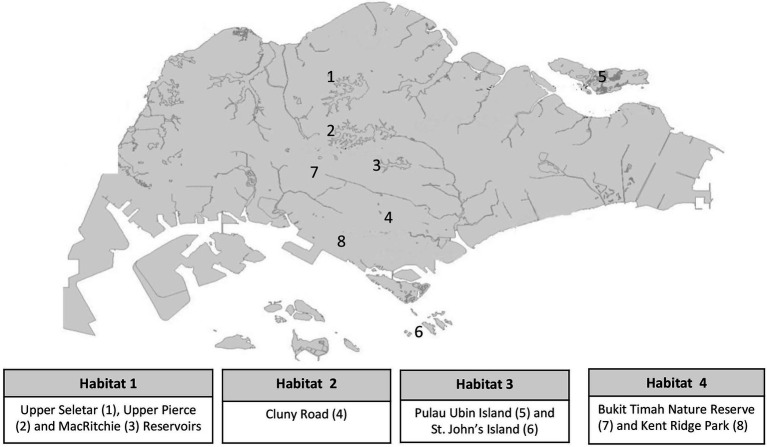
A map of Singapore showing eight locations where the identified fungal endophytes were originally isolated from.

Endophytic fungal isolates selected in the current study were grouped into four categories on the basis of their habitats of isolation in Singapore. *Habitat 1* consists of strains isolated from the three water catchment areas of Upper Seletar, Upper Pierce, and MacRitchie reservoirs. *Habitat 2* consists of strains isolated from Cluny road along Singapore Botanic Gardens. Strains from *Habitat 3* were isolated from two offshore islands of Pulau Ubin and St. John’s, while *Habitat 4* consists of strains isolated from Bukit Timah Nature Reserve and Kent Ridge Park. The substrate and plant tissue details from which the fungal strains were isolated from are given in Supplementary Table S1, while the eight locations from where the isolates were derived from are shown in Figure 1. Reference stock cultures stored at −80°C were revived by sub-culturing on either malt extract agar (MEA, Oxoid, United Kingdom) or potato dextrose agar (PDA, Sigma, United States) plates and incubated at 24°C for 5 days and up to 14 days for the fast-growing and slow-growing strains, respectively.

### Molecular Identification of the Fungal Isolates

Genomic DNA was isolated from mycelia harvested from freshly subcultured plates. Approximately, 100 mg of mycelia from each fungus was scraped using sterile toothpick and grounded to powder in liquid nitrogen using a sterile mortar and pestle. When this method failed to produce quality genomic DNA from some fungal isolates, the following method was used to obtain better quality gDNA: Three mycelial agar disks (5 mm in diameter) from the periphery of an actively growing culture of the fungi grown on malt extract agar (MEA, Oxoid, United Kingdom) were inoculated in a 250 ml flask containing 50 ml malt extract broth (MEB; 30 g malt extract, 5 g mycological peptone/L; pH 5.4 ± 0.2). The flasks were incubated at 24°C in a shaking incubator at 200 rpm for 72 h for the fast-growing strains and up to 7 days for the slow-growing strains. The mycelial mass was harvested by filtering the growth culture through two layers of miracloth followed by drying of the mycelia by thoroughly squeezing out the liquid media through additional layers of miracloth. The mycelia were then ground to powder in liquid nitrogen. Genomic DNA was extracted from the ground mycelia using DNeasy PowerSoil Kit (Qiagen, Germany) following the manufacturers’ instructions. The extracted DNA was quantified using NanoDrop spectrophotometer (Thermo Fisher Scientific, Waltham, MA, United States). Amplification of the internal transcribed spacer 2 (ITS2) region in rDNA gene was carried out using ITS86F (5′-GTGAATCATCGAATCTTTGAA-3′) as the forward primer and ITS4 (5′-TCCTCCGCTTATTGATATGC-3′) as the reverse primer (White et al., 1990; Turenne et al., 1999). The final PCR reaction mixture of 20 μl contained 1 μl gDNA, 1 μl of each of the primers at 10 μM, 2 μl of 2 mM premixed dNTPs, 2 μl of green buffer (Thermo Fisher Scientific, Vilnius, Lithuania), 12.8 μl autoclaved Milli-Q water, and 0.2 μl Dream Taq polymerase (Thermo Fisher Scientific, Vilnius, Lithuania). For the negative control, DNA was replaced with Milli-Q water. The PCR reactions were performed on a Veriti thermal cycler (Applied Biosystems, CA, United States) with the following conditions: initial denaturation at 95°C for 5 min, 40 cycles at 95°C for 30 s, annealing at 60°C for 30 s initial extension at 72°C for 30 s, and a final extension at 72°C for 10 min. The PCR products were electrophoresed on an agarose gels (1× TAE buffer, 1% agarose gel) stained with SYBR safe DNA gel stain (Thermo Fisher Scientific, Carlsbad, CA, United States). Successful PCR amplification was confirmed by visualization of the gel on a ChemiDoc™ MP Imaging System (Bio-Rad, Hercules, CA, United States). Amplified PCR products were purified using MEGAquick-spin total fragment DNA purification kit (iNtRON Biotechnology, South Korea) according to the manufacturer’s instructions. The amplicons were sent for bi-directional sequencing at first BASE DNA sequencing services^1^ using the aforementioned primer pair.

### Phylogenetic Analysis

Generation of accurate consensus sequences for the ITS2 region from the forward (5′–3′) and reverse (3′–5′) sequence raw data for each fungal strain was performed using Benchling alignment editor program.^2^ The alignments were trimmed, overhangs removed, and gaps corrected. The resultant nucleotide sequences were uploaded onto the National Center for Biotechnology Information (NCBI) Basic Local Alignment Search Tool (BLAST; https://blast.ncbi.nlm.nih.gov/Blast.cgi) for the analysis of sequences similarity. The identity of each strain was assigned on the basis of maximum identity score. Phylogenetic analyses were performed using MEGA 7 software with DNA sequences aligned using ClustaIW program embedded within the software (Kumar et al., 2016). The evolutionary relationships between the strains were computed using the Neighbor-Joining and Maximum Likelihood methods. The resultant phylogenetic results were used for taxonomic analyses as well as selection of fungal strains that would undergo fermentation for metabolites extraction.

### Fermentation and Extraction of Fungal Crude Extracts

Fermentation for extracts generation was done as follows: First, all the 146 fungal strains selected for extracts generation following phylogenetic analysis were grown in 10 ml cultures of two in-house liquid media (CF02LB and CF18LB) that have been formulated and optimized by the Natural Product Library group at SIFBI for fungal secondary metabolites production. Thereafter, two approaches, namely chemical elicitation and variation of fermentation media, were evaluated for their potential in enhancing the production of bioactive secondary metabolites from fungal endophytes. To this end, the 146 fungal strains were divided into two groups with the first group of 45 strains grown in the same two liquid media (CF02LB and CF18LB) in the presence of 50 μM 5-azacytidine (Sigma, Canada), a DNA methyltransferase inhibitor and 100 μM of suberoylanilide hydroxamic acid (SAHA, Cayman Chemical Company, United States), a histone deacetylases inhibitor. The second group of 101 fungal isolates were grown in three additional media; one liquid media (CF07LB) and two solid media (CF25ST and CF28ST) in a setup modelled around the one strain-many compounds (OSMAC) approach (Bode et al., 2002; Supplementary Figure S1). Table 1 shows the composition of the five media that were used in this study. The cultures were incubated for 14 days in a shaking incubator at 24°C and 200 rpm for the liquid media cultures and 21 days at 24°C in static growth for the solid media cultures. Subsequently, the cultures were frozen overnight at −80°C and freeze-dried in a vacuum freeze-dryer for 4 days to expel all moisture. Dried cultures were resuspended in methanol and kept in a shaking incubator overnight at 24°C and 200 rpm to obtain the extractables. This was followed by filtration using filter paper (Whatman No. 4) to separate the dissolved metabolites from the insoluble media components and mycelial residues. The extracts were then dried using a centrifugal vacuum concentrator and weighed. Extracts were resuspended in appropriate amount of MeOH, dispensed in 96-well microplate and micronic storage tubes, dried and stored at 4°C until when required for bioassays and chemical analyses.

**Table 1:** Composition of the five media used in the study.

Components	Media (per L)				
	CF02LB	CF07LB	CF18LB	CF25ST	CF28ST
Glucose	20 g	15 g	20 g	–	–
Maltose	10 g	–	–	–	–
Cane molasses	–	20 g	–	–	–
Soluble starch	–	40 g	–	–	–
Pharmamedia	–	25 g	–	–	–
Peptone	–	–	5 g	–	–
Yeast extract	4 g	–	5 g	1 g	0.2 g
KH_2_PO_4_	–	–	0.5 g	0.5 g	0.1 g
MgSO_4_. 7H_2_O	–	–	1 g	–	0.1 g
FeSO_4_. 7H_2_O	–	–	–	–	0.01 g
FeCl_3_	–	–	10 mg	–	–
ZnSO_4_. 7H_2_O	–	–	1.78 mg	–	0.01 g
CaCl_2_	–	–	73.5 mg	–	–
CaCO_3_	–	8 g	–	–	–
Sodium tartrate	–	–	–	0.5 g	0.1 g
Oatmeal	20 g	–	–	–	–
Brown rice	–	–	–	20 g/40 ml	–
Cracked corn	–	–	–	–	5 g/10 ml
pH	7.5	Natural	5.5	Natural	Natural

### Antimicrobial Activity

Four microbial pathogens, bacteria *Staphylococcus aureus* (ATCC 25923) and *Klebsiella aerogenes* (ATCC 13048) and fungi *Candida albicans* (ATCC 10231) and *Aspergillus fumigatus* (ATCC 46645), were used to assess the antimicrobial activities of fungal extracts. Screening of the crude extracts for antimicrobial activity was carried out in a single concentration of 200 μg/ml in duplicate in a 384-well assay in which 45 μl of 5.0 × 10^5^ and 2.5 × 10^3^ CFU/ml of the bacteria, and *C. albicans* cells, respectively, were seeded in each well with the same volume of 2.5 × 10^4^ spore/ml used for *A. fumigatus*. Gentamicin was used as a standard inhibitor in bacterial screens with a 16-point, 2-fold serial dilution prepared with a starting final assay concentration of 25 μg/ml. Amphotericin B was used as the standard inhibitor in fungal screens with a 16-point, 2-fold serial dilution prepared with a starting final assay concentration of 20 μg/ml. Dose response testing of crude extracts was similarly done with a starting concentration of 200 μg/ml in an eight-point serial dilution for samples that had revealed average % inhibition ≥50 in the primary screens. Growth inhibitory activity of fungal extracts against the test pathogens was evaluated by reading the plate’s OD at 600 nm using a plate reader (Tecan Infinite M1000 Pro reader, Switzerland) after 24, 48, and 72 h of incubation for the bacteria, *C. albicans* and *A. fumigatus*, respectively.

### Cytotoxic Activity

Cytotoxic activity was evaluated against three cancer cell lines; the human pancreatic cancer cells PANC-1 and MIA PaCa-2, and the human lung cancer cells A549. Cell stocks stored in liquid nitrogen (−196°C) were subcultured in Dulbecco’s Modified Eagle Medium (DMEM, Gibco, Life Technologies, Bleiswijk, the Netherlands) enriched with 10% fetal bovine serum (FBS, Gibco, Life Technologies, Paisley, United Kingdom) and 1% penicillin–streptomycin solution. The cells were grown for at least two passages in order to allow them to recover and achieve optimal growth conditions necessary for the cytotoxicity screening assays. Primary screening was done at a single concentration of 200 μg/ml in duplicate in a 384-well assay. Once the growing cells had attained confluence, they were washed twice with phosphate-buffered saline (Gibco, Life Technologies, Paisley, United Kingdom), harvested by trypsinization and viable cell density determined by trypan blue exclusion method using a haemocytometer. Cells were seeded in a 384-well black clear bottom plates (Greiner Bio-One, Austria) with 45 μl added into each well at a density of 3.3 × 10^4^ cells/mL for MIA PaCa-2 and A549 and 5.5 × 10^4^ cells/ml for PANC-1. The cells were incubated at 37°C for 24 h in 5% CO_2_ and 95% relative humidity after which 5 μl of extract was added into each well. Puromycin, a protein synthesis inhibitor, was used as the standard inhibitor in which a 16-point, 3-fold serial dilution was prepared with a starting final assay concentration of 184 μM. The plates were incubated for 72 h after which 5 μl of PrestoBlue reagent (Invitrogen, Eugene, Oregon, United States) was added in each well, and the plates incubated for 2 h for A549 and MIA PaCa-2 and 4 h for PANC-1. This was followed by reading of plate’s fluorescence at an excitation wavelength of 560 nm and emission wavelength of 590 nm. Dose response testing of crude extracts was similarly done with a starting concentration of 200 μg/ml in an eight-point serial dilution for samples that had revealed average % inhibition ≥50 in the primary screens.

For the isolated compounds, dose–response testing for both antimicrobial and cytotoxic screens was carried out in triplicate in an eight-point, 2-fold serial dilution assay with a starting concentration of 100 μg/ml. The rest of the assay conditions and controls were performed in a similar manner as in the primary screening.

### Natural Product Extract Dereplication, Compound Isolation, and Structure Elucidation

Active extracts were analyzed according to a dereplication procedures described in the literature (Butler et al., 2012). Three fungal strains, F6932, F10154, and F5912 whose extracts contained active constituents of interest, were fermented in large-scale to obtain sufficient crude extracts for compound isolation. The fungal strains were grown in 50 ml media in 250 ml glass conical flasks by inoculating three mycelial discs (5 mm in diameter) into each of the media flask. F6932 was grown in CF02LB media with 50 μM of 5-azacytidine, while F10154 and F5912 were grown in CF28ST media. The fermentation conditions were similar to those used for small-scale fermentation. At the end of incubation period, the culture flasks were frozen overnight at −80°C and then, freeze-dried in a vacuum freeze-dryer for 7 days to expel all the moisture. About 50 ml of methanol was added to each flask and the flasks kept in a shaking incubator overnight at 24°C and 200 rpm to extract the metabolites. This was followed by filtration through Whatman filter paper (No. 4) to separate the dissolved metabolites from the media and mycelial residues. For each fungal strain, the filtrate from all the flasks was combined into a single large flask, dried using a rotary evaporator and the weight of the dry extracts determined. The dried extracts obtained from strains F6932, F10154, and F5912 were soaked and resuspended with 20 ml MeOH, sonicated for 5 min, and centrifuged to separate the insoluble from the soluble. The supernatants were transferred to 50 ml round bottom flasks and dried using Buchi rotary evaporator. The dried enriched samples were dissolved in 2.5 ml MeOH, centrifuged, and the supernatants were then subjected to C_18_ reversed-phase preparative HPLC purification. Enriched sample obtained from *Lophiotrema* sp. F6932 was separated by the following condition (solvent A: H_2_O + 0.1% HCOOH, solvent B: MeCN + 0.1% HCOOH; flow rate: 30–52 ml/min, gradient conditions: 85:15 isocratic for 5 min, 30 ml/min; followed by 85:15 isocratic for 5 min, 52 ml/min; 15%–55% of solvent B over 30 min, 55%–100% of solvent B over 22 min, and finally isocratic at 100% of solvent B for 10 min) to give 2.2 mg of palmarumycin CP_30_ (**1**) and 3.4 mg of palmarumycin C_8_ (**2**). Enriched sample obtained from *Muyocopron laterale* F5912 was separated by the following condition (solvent A: H_2_O + 0.1% HCOOH, solvent B: MeCN + 0.1% HCOOH; flow rate: 30–52 ml/min, gradient conditions: 75:25 isocratic for 5 min, 30 ml/min; followed by 75:25 isocratic for 5 min, 52 ml/min; 25%–65% of solvent B over 50 min, 65%–100% of solvent B over 10 min, and finally isocratic at 100% of solvent B for 17 min) to give 2.2 mg of muyocopronol A (**3**), 5.5 mg of muyocopronol B (**4**), and 1.6 mg of muyocopronol C (**5**). Enriched sample obtained from *Colletotrichum tropicicola* F10154 was separated by the following condition (solvent A: H_2_O + 0.1% HCOOH, solvent B: MeCN + 0.1% HCOOH; flow rate: 30–52 ml/min, gradient conditions: 80:20 isocratic for 5 min, 30 ml/min; followed by 80:20 isocratic for 5 min, 52 ml/min; 20%–50% of solvent B over 5 min, 50%–100% of solvent B over 47 min, and finally isocratic at 100% of solvent B for 10 min) to give 8.3 mg tropicicolide (**6**).

### General Analytical Chemistry Procedures

Specific rotations were measured using JASCO P-2000 polarimeter. NMR spectra were obtained from Bruker DRX-400 NMR spectrometer equipped with Cryoprobe. The NMR spectrometer used a 5-mm BBI (^1^H, G-COSY, multiplicity-edited G-HSQC, and G-HMBC spectra) or BBO (^13^C spectra) probe heads equipped with z-gradients. Agilent 1260 Infinity Preparative-Scale LC/MS Purification System equipped with Agilent 6130B single quadrupole mass spectrometer detector was used to conduct preparative HPLC analyses. Agilent 5 Prep C_18_ column (10 mm × 30 mm) was used for HPLC runs. HPLC-MS experiment was performed on an Agilent UHPLC 1290 Infinity coupled with a diode array detector (DAD), and an Agilent 6540 accurate-mass quadrupole time-of-flight (QTOF) mass spectrometer equipped with a splitter and an ESI source. The analyses were conducted with Acquity UPLC BEH C18 column (2.1 mm × 50 mm, 1.7 μm) under standard gradient condition of 98% (0.1% formic acid) to 100% MeCN (0.1% formic acid) over 8.6 min, at a flow rate of 0.5 ml/min. The operating parameters for QTOF were the same as in (Sirota et al., 2018).

## Results

### Characterization and Diversity of Endophytic Fungi

A total of 222 fungal strains were successfully revived and identified *via* molecular sequencing of the ITS2 region of rDNA gene (Supplementary Table S1). The sequences generated in this study were deposited to the National Center for Biotechnology Information (NCBI) database GenBank with accession numbers OM791857-OM792078. Of the identified fungal strains, 23 had been isolated from the three water catchment areas of Upper Seletar Reservoir Park, Upper Pierce Reservoir, and MacRitchie Reservoir (*Habitat* 1), 89 strains were from Cluny road along Singapore botanic garden (*Habitat* 2), 39 strains were isolated from Pulau Ubin and St. John’s islands (representing *Habitat* 3), while 71 strains were isolated from Bukit Timah Nature Reserve and Kent Ridge Park designated as *Habitat* 4 (Figure 1). With regard to the plant tissue/substrate from which the fungal strains were isolated, majority (65%; 145 strains) of the strains had been isolated from the leaves followed by (19%; 41 strains) the stems. The fungal strains that were isolated from the flowers and fruits were 17 (8%) and 5 (2%), respectively. The remaining fungi were isolated from specialized plant organs such as the frond of palm trees and ferns. Among the identified fungal isolates, 21 strains (an equivalent of 9% of all the studied fungal strains) had <97% sequence similarity in the ITS2 rDNA gene region when compared with the closest relative in the NCBI (Supplementary Table S1). From the 222 identified fungal strains, 146 fungal strains were selected on the basis of phylogenetic analysis to undergo fermentation for extracts generation.

**Figure 2:**
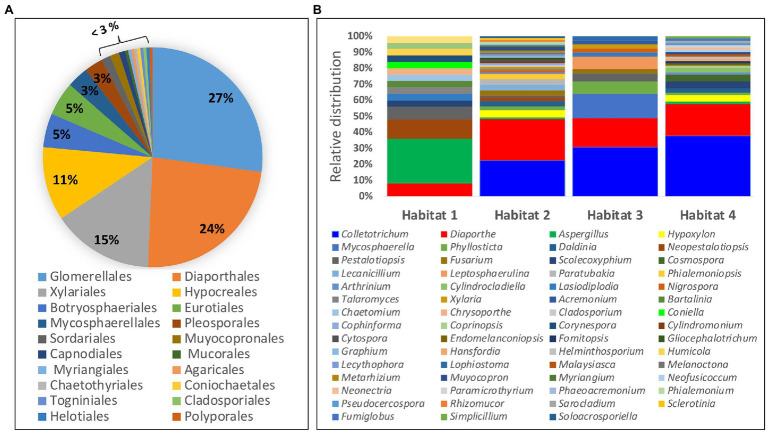
Diversity and distribution of identified endophytic fungi: **(A)** Percentage of fungal strains belonging to each of the 20 identified orders. **(B)** Relative distribution of fungal endophytes from different genera within the four study habitats.

Among the 222 endophytic fungal strains investigated in this study, 219 strains belong to the phylum *Ascomycota*, while two strains belonged to the phylum *Basidiomycota* and only one strain was from *Mucoromycota* phylum. Class *Sordariomycetes* accounted for 79% of all the identified ascomycete strains followed by *Dothideomycetes* which accounted for 14% of all the fungal strains belonging to this phylum. The identified fungal strains were distributed into 20 orders with *Glomerellales* and *Diaporthales* accounting for the highest number of strains (Figure 2A). Two fungal isolates belonging to the class *Dothideomycetes* lack a placement at the level of order and are designated as *incertae sedis* (Supplementary Table S1). At the genus level, the fungal strains were distributed into 59 genera of which the dominating genera were *Colletotrichum* (27%), followed by *Diaporthe* and anamorph *Phomopsis* constituting 24% of the identified fungal strains. Others include *Aspergillus* (4%) and *Hypoxylon*, *Mycosphaerella*, and *Phyllosticta* each accounting for 3% of all the fungal strains (Figure 2B). Furthermore, the majority of the identified genera (63%; 37 genera) were represented by a single fungal strain with the bulk of the identified fungal genera (73%; 43 genera) occurring in only one of any of the four studied habitats. Morphological diversity of selected fungal endophytes from each of the four studied habitats is shown in Supplementary Figure S2.

### Effects of Chemical Elicitors and Growth Media on Extractable Yields

The growth of fungal endophytes in media supplemented with the two chemical elicitors resulted in markedly increase in the weight of crude extracts. Generally, it was observed that crude extracts were in some cases one to three orders of magnitude higher in cultures grown in the presence of the two chemical elicitors when compared with extracts derived from the same strains grown in the absence of the two chemical elicitors. For example, of the 45 fungal strains that were grown in two liquid media in the presence and absence of the chemical elicitors, nine out of the 10 highest extractable yields were obtained from strains grown in the presence of the chemical elicitors. Furthermore, nine out of 10 lowest yields were obtained from fungal strains grown in the absence of the chemical elicitors. With regard to the fungal strains that were grown in five different media, it was observed that the weight of crude extracts was generally higher for cultures that were grown in solid media in comparison with those derived from liquid growth media (Supplementary Figure S3).

### Antimicrobial and Cytotoxic Activity of Fungal Extracts

Fungal endophytes are renowned for their capacity to synthesize bioactive secondary metabolites. In this study, phylogenetic analysis of 222 fungal strains resulted in 146 fungal strains being selected for fermentation in two liquid media (CF02LB and CF18LB) for the assessment of bioactive metabolites production. Furthermore, in order to assess the effect of chemical epigenetic modifiers and variation of growth media on the biosynthesis of bioactive secondary metabolites, the 146 strains were further divided into two groups. The first group of 45 strains was grown in the same two liquid media (CF02LB and CF18LB) in the presence of two chemical elicitors; 50 μM of 5-azacytidine and 100 μM of SAHA. The second group of 101 strains was grown in three additional media (CF07LB, CF25ST, and CF28ST).

**Figure 3:**
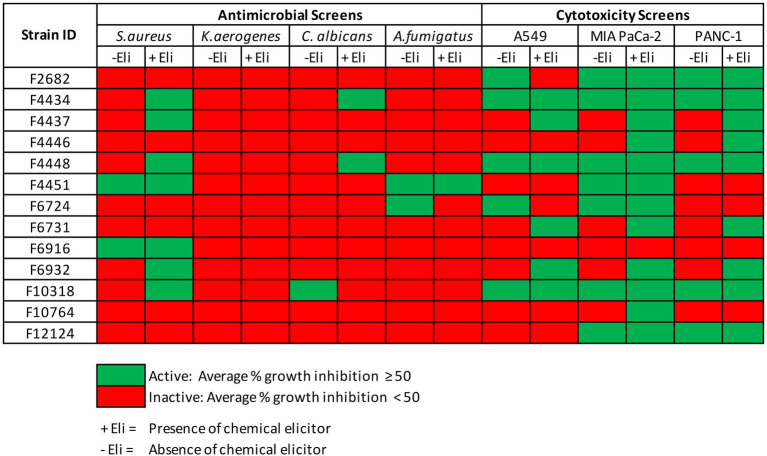
Antimicrobial and cytotoxic screens hits from 45 fungal strains grown in CF02LB and CF18LB media in the presence and absence chemical elicitors.

For the 45 fungal strains grown in two liquid media in the presence and absence of the two chemical elicitors, a total of eight strains revealed antimicrobial activity against at least one of the four studied microbial pathogens (average % growth inhibition ≥50). Significantly, four of these strains (F4434, F4437, F4448, and F6932), an equivalent of 50% of the active strains, revealed antimicrobial activity only when grown in the presence of chemical elicitors (Figure 3). For cytotoxic activity, out of the 45 strains grown in the presence and absence of chemical elicitors, 12 fungal strains exhibited cytotoxic activity against at least one of the three tested cell lines. Importantly, chemical elicitation results revealed that five of these fungal strains (F4437, F4446, F6731, F6932, and F10764) presented cytotoxic activities when grown exclusively in the presence of chemical elicitors (Figure 3). Elicitor-derived extracts from F4437, F6731, and F6932 exhibited cytotoxic activities against all three cell lines evaluated, while extracts from F4446 displayed cytotoxic activity against MIA PaCa-2 and PANC-1 cell lines, and extracts from F10764 exhibited inhibitory activity against MIA PaCa-2 cells only (Figure 3). The overall distribution of antimicrobial and cytotoxic hits from 45 fungal strains grown in the presence of two chemical elicitors and in the absence of chemical elicitation is shown in Supplementary Figure S4.

**Figure 4:**
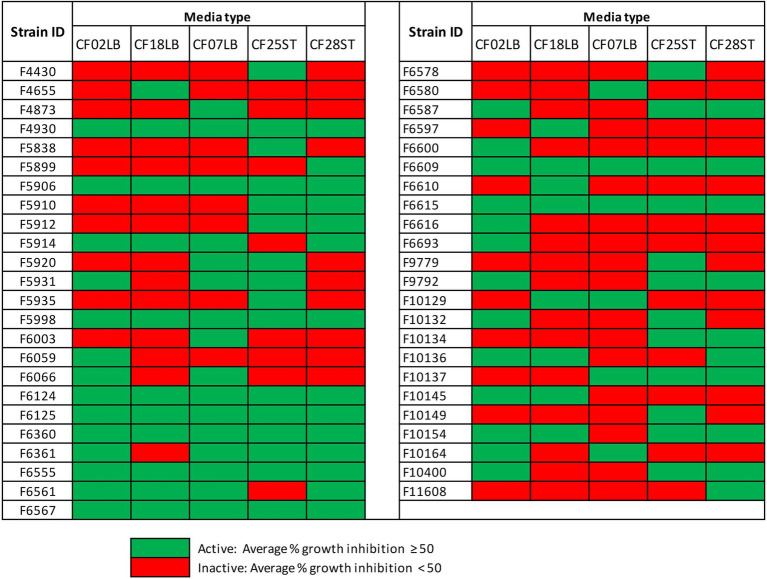
Hits from 101 fungal strains grown two initial media (CF02LB, CF18LB) and three additional media (CF07LB, CF25ST, and CF28ST).

For the 101 fungal strains that were grown in five different media, two initial media (CF02LB and CF18LB) and three additional media (CF07LB, CF25ST, and CF28ST), there were a total of 47 strains that exhibited biological activity against at least one of the tested panel of pathogens and cancer cell lines (Figure 4). Notably, 16 of these fungal strains, an equivalent of 34% of all the active strains, exhibited biological activity only when grown in the three additional media. In other words, compared with the use of two initial growth media, the growth of fungal strains in three additional media resulted in 16 additional hit strains (Figure 4). Among the three additional fermentation media, CF25ST had the highest number of active strains (28 strains) followed by CF28ST and CF07LB with 24 and 22 active strains, respectively (Figure 4). Supplementary Figure S5 shows the overall, distribution of antimicrobial and cytotoxic hits from the 101 fungal strains that were grown in five different media.

**Figure 5:**
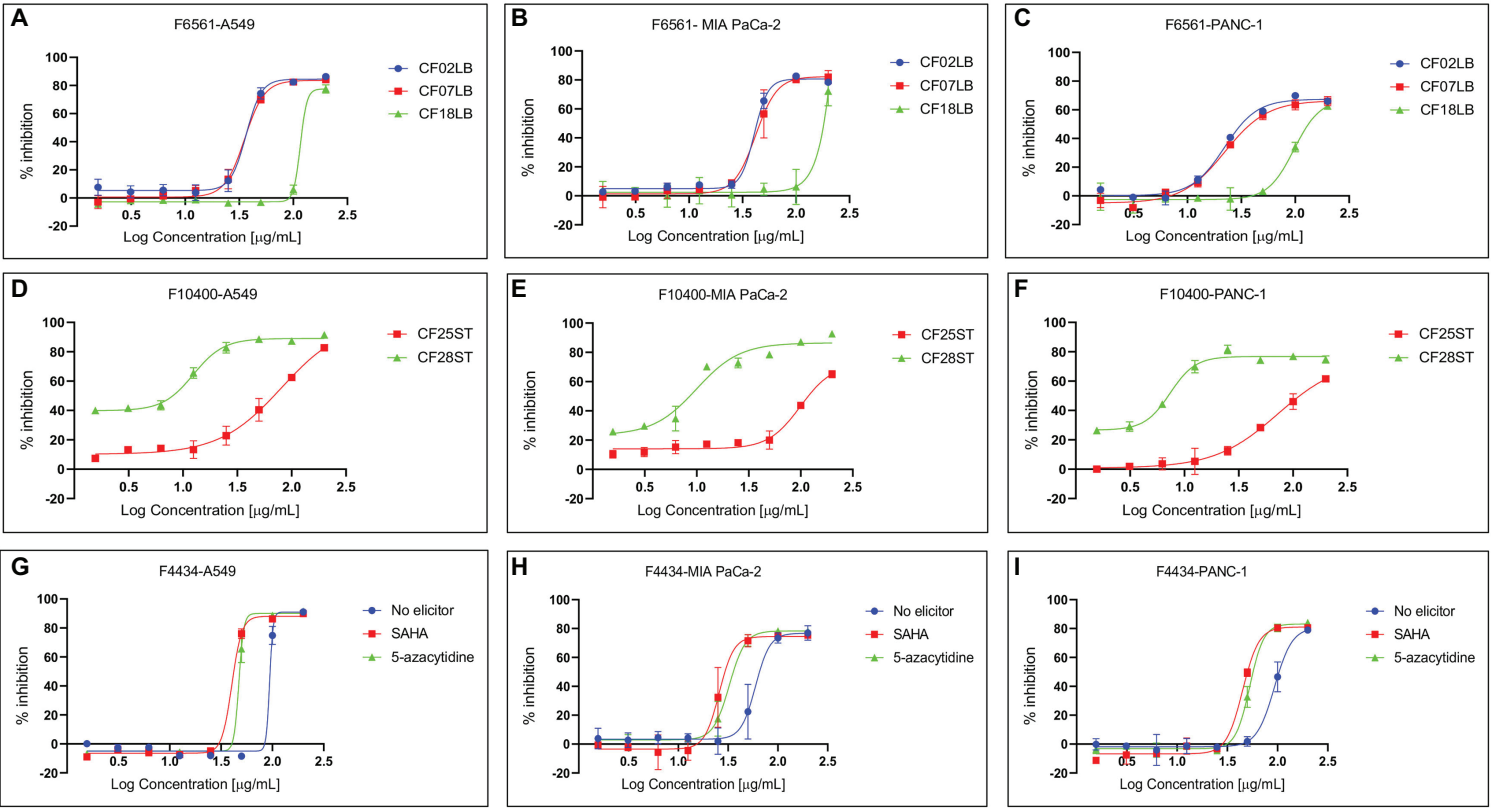
Differential expression of cytotoxic activity of extracts derived from three fungal endophytes against three cancer cell lines—A549, MIA PaCa-2, and PANC-1. **(A–C)** Show the activity of extracts from F6561 grown in three different liquid media CF02LB, CF07LB, and CF18LB, **(D–F)** show activity of extracts derived from F10400 grown in two solid media CF25ST and CF28ST, while **(G–I)** show the activity of extracts from strain F4434 grown in CF02LB media in the absence of a chemical elicitor (control) and in the presence of 50 μM 5-azacytidine and 100 μM SAHA.

In a number of instances, there were observable differences in the expression of biological activities by crude extracts derived from the same fungal strains when grown in the presence or absence of chemical elicitor or in different growth media. Figure 5 shows differential expression of cytotoxic activity of extracts generated from three selected fungal strains (F4434, F6561, and F10400) when subjected to varied fermentation regimes.

In summary, antimicrobial and cytotoxic screening results revealed that 63 fungal endophytes, representing 43% of all the fungal strains screened, possessed biological activity against at least one of the tested microbial pathogens or cancer cell lines. Overall, 34 strains had cytotoxic activity, 10 strains demonstrated antimicrobial activity, while 19 strains exhibited both antimicrobial and cytotoxic activity against at least one of the tested panel of pathogens and cancer cells lines, respectively. The active fungal strains were distributed into 11 orders with *Diaporthales* accounting for the highest number of active strains (33%; 21 strains) followed by *Hypocreales* and *Glomerellales* which accounted for 17 and 14% of all the active fungal strains, respectively (Supplementary Figure S6). Chemical elicitation resulted in induced antimicrobial and/or cytotoxic activity in seven fungal strains while growth of fungal strains in three additional media resulted in induced biological activity in 21 fungal strains. Among these are strains F6932 and F5912 whose biological activities were only uncovered as a result of chemical elicitation and variation of fermentation media, respectively. F6932 exhibited antibacterial activity only when grown in CF02LB media in the presence of an elicitor (Figure 3), while F5912 exhibited antimicrobial activity only when grown in two (CF25ST and CF28ST) out the five fermentation media (Figure 4). The two strains together with strain F10154 that had shown biological activity when grown in four out of the five tested media were progressed for further studies following the identification of active constituents of interest.

### Chemical Dereplication of Active Extracts From Selected Fungal Endophytes

Among the active samples, extracts from 28 fungal strains grown under different conditions and exhibiting promising biological activities were subjected to chemical dereplication. The extracts were analyzed by high-resolution mass spectroscopy for the identification of molecules by matching their accurate mass with the updated Dictionary of Natural Products (DNP) database for fungal natural products.^3^ This resulted in the putative identification of numerous known compounds based on their accurate mass. Among the known compounds identified from the three *Aspergillus* strains are aspergiterpenoid A, asnovolin G, secalonic acid A and G, and neoxaline. In the case of *Phomopsis*/*Diaporthe* sp., epicoccamide A, epicoccamide D, antibiotic M 6124, and 6-epicerevisterol were detected. Mycotoxins such as fumonisin B1 and fumonisin A1 in addition to one other known metabolite: glisoprenin E were dereplicated from two *Fusarium* strains (Supplementary Table S2). The exact identification of several of these compounds was performed by HR-LCMS/MS comparison with our in-house authentic standard compounds library or with MS/MS spectra reported in the literature (Tamura et al., 2014; Grigoletto et al., 2020).

### Isolation and Structural Elucidation of Bioactive Compounds From Three Prioritized Strains: F6932, F5912, and F10154

Based on the chemical dereplication studies, we did not find any known molecules that could explain the activities observed in three different fungal cultures, *Lophiotrema* sp. F6932 fermented in CF02LB supplemented with 5-azacytidine, *M. laterale* F5912 and *C. tropicicola* F10154 both grown in CF28ST media. Therefore, these strains were selected for further investigation to identify the molecules that were responsible for the observed antimicrobial activities. Chemical analysis of the extracts from the three aforementioned strains led to the identification of a new compound palmarumycin CP_30_ (**1**) and a known compound palmarumycin C_8_ (**2**) from *Lophiotrema* sp. F6932, three novel polyketides, muyocopronols A (**3**), B (**4**), and C (**5**) from *M. laterale* F5912, and a novel 26-membered macrolide designated as tropicicolide (**6**) from *C. tropicicola* F10154.

**Figure 6:**
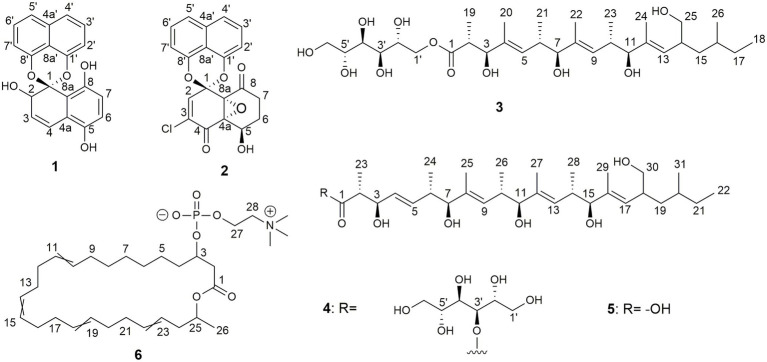
Chemical structures of palmarumycin CP_30_ (**1**) and palmarumycin C_8_ (**2**), muyocopronol A (**3**), B (**4**), and C (**5**), and tropicicolide (**6**) that were isolated from strains, *Lophiotrema* sp. F6932, *Muyocopron laterale* F5912, and *Colletotrichum tropicicola* F10154.

**Figure 7:**
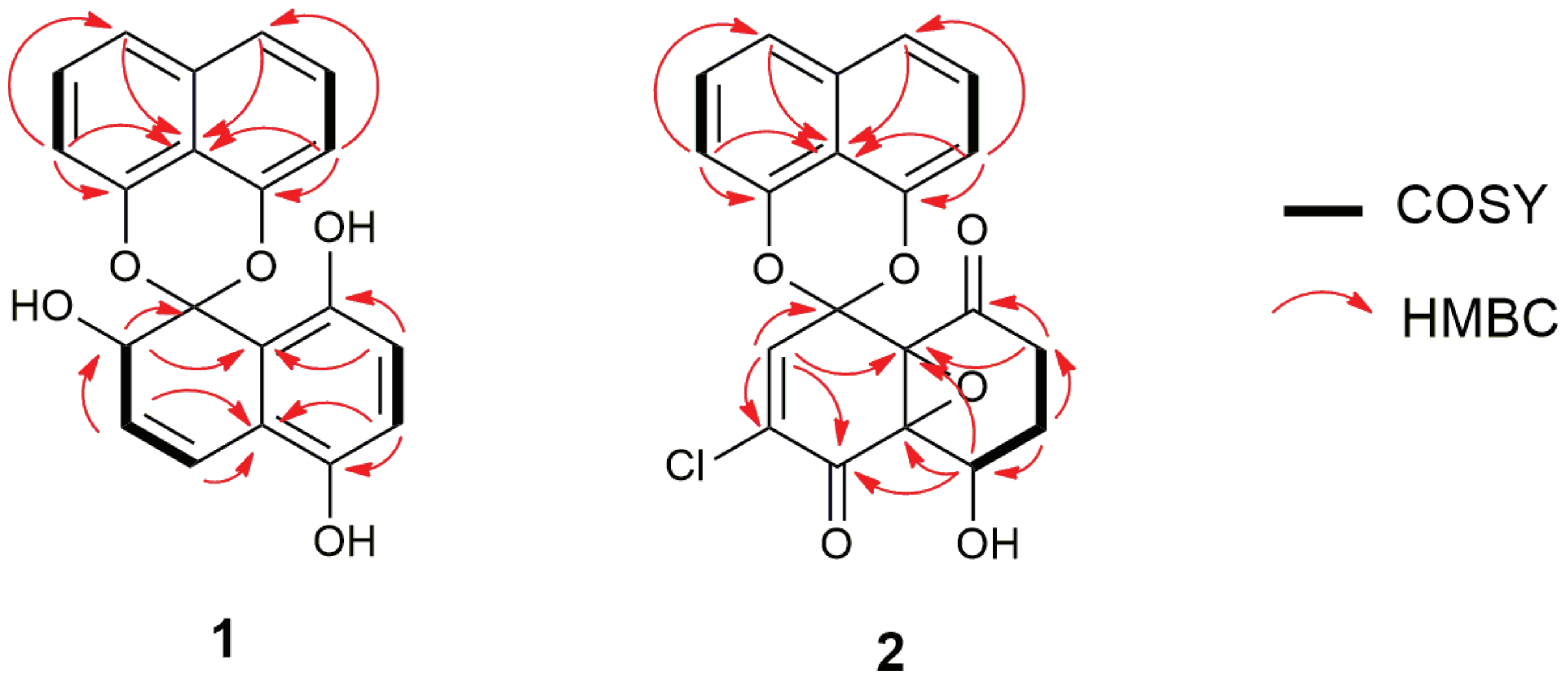
Selected COSY and HMBC correlations of palmarumycin CP_30_ (**1**) and palmarumycin C_8_ (**2**).

Compound **1** (Figure 6) was isolated as a brownish amorphous powder with [α]_D_ + 130 (c 0.6, MeOH). HR-ESIMS measurement established the molecular formula C_20_H_14_O_5_. As shown in Table 2 and Supplementary Figure S7, the ^1^H NMR data revealed the presence of six naphthalene proton multiplets at *δ*_H_ 6.87–7.60. One multiplet at *δ*_H_ 4.20 was assigned to a methine proton of secondary hydroxy group. The assignment of the olefin group at C-3 and C-4 was supported by the observation of vinyl carbons at *δ*_C_ 124.9 and 124.6, as well as olefinic protons at *δ*_H_ 5.89 and 7.02 in the ^1^H and ^13^C NMR spectra (Supplementary Figures S7, S8), respectively. The 1D and 2D-NMR data also revealed the presence of two adjacent aromatic protons, i.e., *δ*_H_ 6.84 and 6.71, found in a 1,4-methoxybenzene ring (Figures 6, 7). Based on these data and the observed COSY and HMBC correlations (Figure 7), the structure of **1** was established as a new member of the spirobisnaphthalene palmarumycin family and named palmarumycin CP_30_ (**1**). The stereochemical determination at C-2 was unfeasible using spectroscopic method and Mosher’s ester analysis. Hence, the stereochemistry in **1** remained undetermined.

**Table 2:** NMR spectral data of palmarumycin CP_30_ (**1**) and palmarumycin C_8_ (**2**).

Position	1*^a^*		2*^b^*	
	^13^C, type	^1^H, mult. (*J* = Hz)	^13^C, type	^1^H, mult. (*J* = Hz)
1	105.5, C		95.6, C	
2	64.5, CH	4.20, d (5.8)	135.4, CH	6.77, s
3	124.9, CH	5.89, dd (5.8, 9.9)	131.4, C	
4	124.8, CH	7.02, d (9.9)	185.4, C	
4a	121.8, C		63.0, C	
5	151.4, C		61.5, CH	5.12, t (3.0)
6	120.1, CH	6.84, d (8.9)	23.3, CH_2_	2.09, m
7	119.7, CH	6.71, d (8.9)	32.8, CH_2_	2.51, m, 2.79, m
8	148.0, C		194.3, C	
8a	116.2, C		61.3, C	
1′	147.8, C		144.6, C	
2′	111.3, CH	7.16, dd (0.7, 7.5)	110.4, CH	7.10, d (7.5)
3′	128.7, CH	7.51, m	127.7, CH	7.49, t (7.7)
4′	122.6, CH	7.60, dd (0.6, 8.4)	121.6, CH	7.58, d (8.8)
4a′	135.5, C		134.2, C	
5′	121.6, CH	7.53, dd (0.6, 8.1)	121.6, CH	7.56, d (8.7)
6′	128.5, CH	7.44, m	127.4, CH	7.43, t (7.9)
7′	110.2, CH	6.87, dd (0.6, 7.5)	109.5, CH	6.93, d (7.5)
8′	149.0, C		145.2, C	
8a′	114.9, C		112.4, C	

a ^1^H (400 MHz) and ^13^C (100 MHz) in methanol-*d*_4_.

b ^1^H (400 MHz) and ^13^C (100 MHz) in chloroform-*d*.

Assignments based on COSY, HSQC, and HMBC and comparison with the literature compounds. Chemical shifts (δ) in ppm. s, singlet; d, doublet; dd, doublet of doublet; t, triplet; and m, multiplet. One proton unless otherwise stated.

Compound **2** (Figure 6) was isolated as a brownish amorphous solid with [α]_D_ + 54 (c 1.2, MeOH) and established as C_20_H_13_ClO_6_ on the basis of HR-ESI-MS measurement. As shown in Table 2 and Supplementary Figure S13, the ^13^C NMR data showed two oxy-quaternary carbons, positions C-4a and C-8a at δ 63.0 and 61.3, respectively. The resonance at *δ*_C_ 185.4 and 194.3 indicated two carbonyl carbons at C-4 and C-8 (Supplementary Figure S13), respectively. The presence of a 4-hydroxycyclohexanone moiety is supported by the COSY correlations from H-5 to H_2_-7 and HMBC correlation from H_2_-7 to carbonyl C-8. The carbonyl carbon atom at *δ*_C_ 185.4 was established at C-4 based on the HMBC correlation from H-5 to C-4 (Figure 7). Singlet for vinyl proton at *δ*_H_ 6.77 (Supplementary Figure S12) was established at C-2, while the signal of a chloro-substituted carbon atom at *δ*_C_ 131.4 in the ^13^C NMR spectrum (Supplementary Figure S13) was established at C-3 based on the HMBC cross-peaks from H-2 to oxy-quaternary carbon C-1, vinyl carbon C-3, and carbonyl C-4 (Figure 7). In addition, the ^1^H and ^13^C NMR data of **2** were consistent with those reported for palmarumycin C_8_. The *cis* configuration of the epoxide oxygens and the stereochemistry of the proton at C-5 were assigned in agreement with Krohn et al. (1994). Hence, **2** was identified as the known spirobisnaphthalene, palmarumycin C_8_.

**Figure 8:**
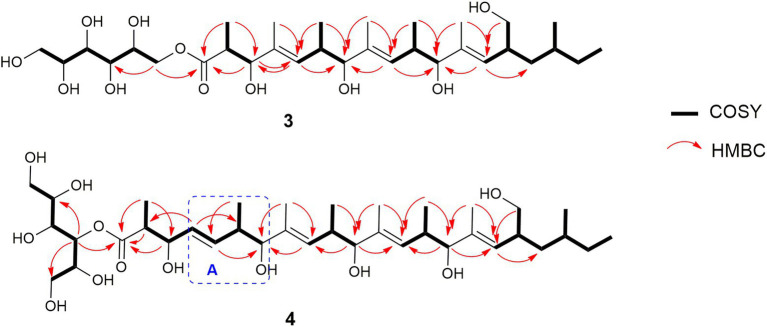
Selected COSY and HMBC correlations of muyocopronol A (**3**) and muyocopronol B (**4**).

Compound **3** (Figure 6) was obtained as brownish amorphous powders with [α]_D_ + 68 (c 0.2, MeOH) and a molecular formula of C_32_H_58_O_11_ as suggested by (−)-HRESIMS data. The ^1^H, ^13^C, and HSQC NMR data revealed the presence of eight methyl, five methylene, 15 methine, and four non-protonated carbons (Table 3; Supplementary Figures S17, S18, S20). Following the literature search, the NMR features of **3** were found to be similar to those of bionectriol C (Wang et al., 2014), except for the presence of an additional oxygenated methylened group in **3**, and that the hexopyranose, one methylene, and two methyl moieties in bionectriol C were missing in **3**. The ^13^C NMR chemical shifts at *δ*_C_ 65.2, 68.0, 70.0, 70.1, 70.4, and 73.0 indicated the presence of a mannitol unit in **3**, which was also supported by COSY correlations from H_2_-1′ along the chain to H_2_-5′ (Figure 8). The mannitol unit was connected to the ester carbonyl C-1 based on HMBC correlation from H_2_-1′ to C-1. Further, COSY and HMBC correlations shown in Figure 8 which established the planar structure of **3**.

**Table 3:** NMR spectral data of muyocopronols A (**3**), B (**4**), and C (**5**).

Position	**3** *^a^*		**4** *^a^*		**5** *^a^*	
	^13^C, type	^1^H, mult. (*J* in Hz)	^13^C, type	^1^H, mult. (*J* in Hz)	^13^C, type	^1^H, mult. (*J* in Hz)
1	177.8, C		177.2, C		182.7, C	
2	44.7, CH	2.65, m	47.6, CH	2.55, m	48.4, CH	2.33, m
3	82.2, CH	4.08, d (10.1)	76.9, CH	4.13, dd (8.2, 8.2)	76.8, CH	4.05, dd (7.5, 7.5)
4	136.0, C		131.5, CH	5.44, dd (15.2, 8.2)	132.8, CH	5.49, dd (15.3, 7.5)
5	135.3, CH	5.32, d (8.4)	139.6, CH	5.71, dd (15.2, 8.2)	137.2, CH	5.70, dd (15.3, 7.5)
6	36.61, CH	2.65, m	41.1, CH	2.33, m	41.1, CH	2.33, m
7	84.4, CH	3.70, m	84.0, CH	3.67, m	83.9, CH	3.66, m
8	137.6, C		137.3, C		137.6, C	
9	133.8, CH	5.29, d (9.6)	134.0, CH	5.25, d (9.4)	133.60, CH	5.26, d (8.2)
10	36.65, CH	2.65, m	36.3, CH	2.64, m	36.65, CH	2.65, m
11	84.6, CH	3.70, m	84.8, CH	3.67, m	88.4, CH	3.69, d (8.9)
12	138.8, C		137.6, C		137.7, C	
13	132.6, CH	5.09, d (10.1)	133.9, CH	5.25, d (9.4)	133.64, CH	5.28, d (8.2)
14	44.7, CH	2.65, m	36.4, CH	2.64, m	36.70, CH	2.65, m
15	39.7, CH_2_	1.22, m	84.7, CH	3.67, m	88.6, CH	3.69, d (8.9)
16	33.4, CH	1.32, m	138.9, C		138.8, C	
17	31.8, CH_2_	1.33, m	132.8, CH	5.07, d (9.8)	132.4, CH	5.09, d (9.1)
18	11.5, CH_3_	0.87, m	39.7, CH	2.64, m	39.7, CH	2.65, m
19	14.7, CH_3_	0.98, d (7.0)	39.6, CH_2_	1.20, m	39.8, CH_2_	1.20, m
20	10.7, CH_3_	1.64, d (1.1)	33.4, CH	1.32, m	33.4, CH	1.33, m
21	17.7, CH_3_	0.79, d (6.7)	31.8, CH_2_	1.21, m	31.8, CH_2_	1.29, m
22	11.5, CH_3_	1.68, d (0.9)	11.8, CH_3_	0.86, m	11.8, CH_3_	0.86, m
23	17.6, CH_3_	0.79, d (6.7)	14.5, CH_3_	1.09, d (7.0)	15.2, CH_3_	1.10, d (7.2)
24	11.8, CH_3_	1.70, d (1.0)	17.7, CH_3_	0.84, m	17.58, CH_3_	0.84, m
25	67.5, CH_2_	3.37, m 3.45, m	11.4, CH_3_	1.64, d (0.9)	11.3, CH_3_	1.65, d (1.0)
26	19.3, CH_3_	0.87, m	17.57, CH_3_	0.78, d (4.8)	17.73, CH_3_	0.78, d (5.7)
27			11.2, CH_3_	1.69, d (1.0)	11.4, CH_3_	1.68, d (1.0)
28			17.56, CH_3_	0.76, d (4.8)	17.67, CH_3_	0.79, d (5.7)
29			11.1, CH_3_	1.67, d (0.9)	11.6, CH_3_	1.69, d (1.0)
30			67.6, CH_2_	3.33, m 3.47, m	67.5, CH_3_	3.37, m 3.44, m
31			19.3, CH_3_	0.86, m	19.3, CH_3_	0.86, m
1′	68.0, CH_2_	4.20, dd (11.4, 6.2) 4.47, dd (11.4, 2.7)	64.5, CH_2_	3.54, m 3.64, m		
2′	70.4, CH	3.91, m	71.7, CH	3.93, m		
3′	70.1, CH	3.81, m	73.9, CH	5.20, dd (7.9, 1.4)		
4′	70.0, CH	3.81, m	71.0, CH	3.90, m		
5′	73.0, CH	3.70, m	72.0, CH	3.53, m		
6′	65.2, CH_2_	3.65, m 3.82, m	65.0, CH_2_	3.64, m 3.81, m		

a ^1^H (400 MHz) and ^13^C (100 MHz) in methanol-*d*_4_.

Assignments based on COSY, HSQC, and HMBC and comparison with the literature compounds. Chemical shifts (δ) in ppm. d, doublet; dd, doublet of doublet; t, triplet; and m, multiplet. One proton unless otherwise stated.

**Figure 9:**
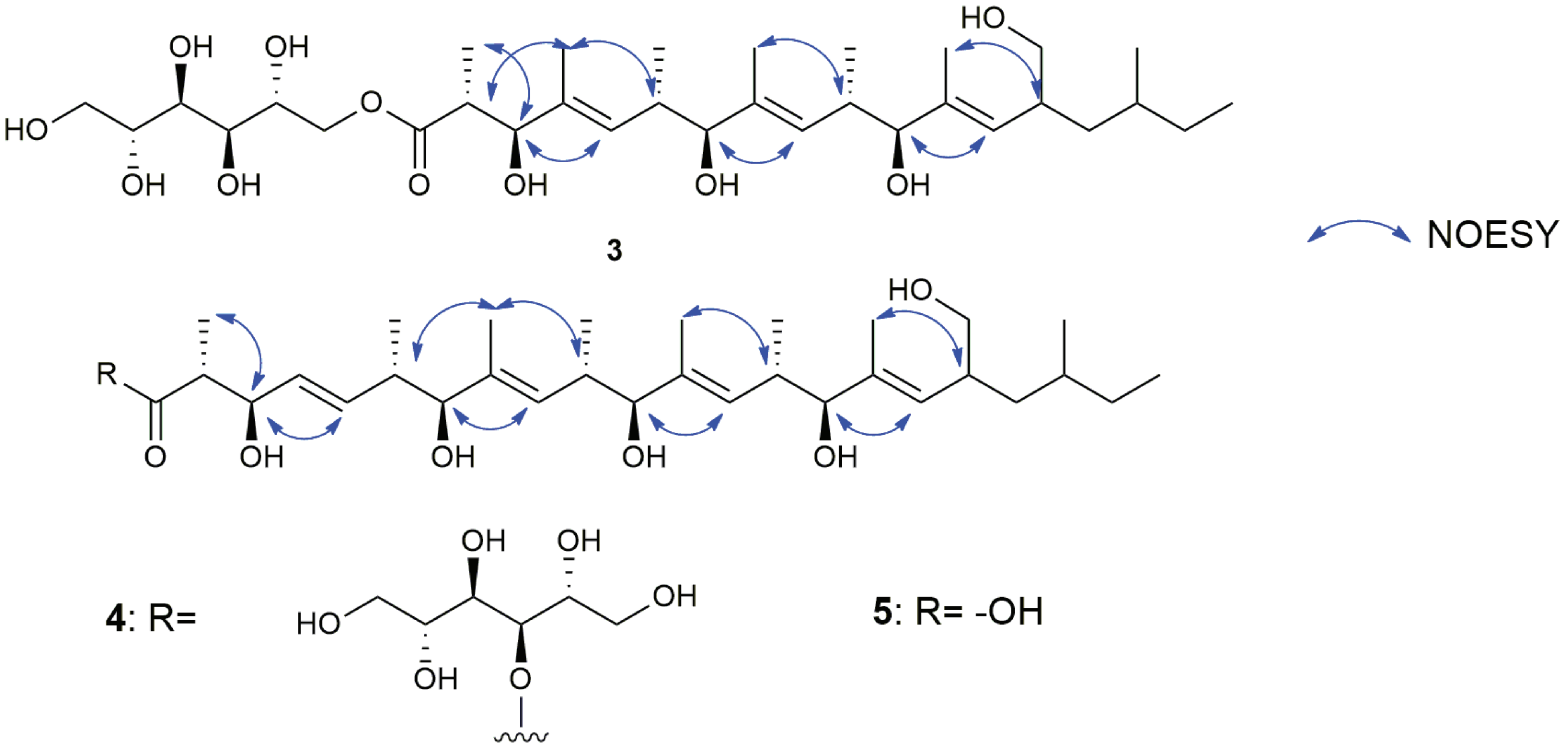
Selected NOESY correlations of muyocopronols A (**3**), B (**4**), and C (**5**) to illustrate the stereochemistry of the olefin groups in the muyocopronol analogs.

The *E*-configurations of the three olefins in **3** were determined by NOESY correlations (Figure 9) between H_3_-20 and H-6, H_3_-22, and H-10, as well as H_3_-24 and H-14. The relative configuration of the mannitol in **3** was deemed to be the same to those of the corresponding part of bionectriol C, since the ^1^H and ^13^C NMR data and ^1^H-^1^H couplings for the mannitol unit in **3** and bionectriol C were identical (Wang et al., 2014). The large ^1^H-^1^H coupling constant (10.1 Hz) between H-2 and H-3 suggested an *erythro* configuration, which was also supported by NOESY correlation between H-3 and H_3_-19 (Kasai et al., 2005). Based on these data, the structure of **3** was established as a new polyketide and named muyocopronol A (**3**).

Compound **4** (Figure 6) was isolated as yellowish white powders with [α]_D_ + 33 (c 0.8, MeOH) and assigned the molecular formula C_37_H_66_O_12_ by (−)-HRESIMS analysis. Comparison of 1D and 2D NMR data of **4** with those of **3** revealed the presence of additional of one methyl, two methine (one of which was oxygenated), and two olefinic methines. COSY correlations between H-4 and H-5, H-5 and H-6, and H-6 and H_3_-24 and between H-6 and H-7 constructed an additional C_5_-substructure in **4**, as shown in Figure 8 (fragment A). The position of the extra C_5_-fragment was suggested by HMBC correlations from H-4 to C-2 and from H_3_-25 to C-7. The ^1^H-^1^H coupling constant of H-4/H-5 indicated a *trans*-configured double bond. The remaining part of the polyketide structural unit was assigned by detailed analysis of COSY and HMBC data (Figure 8). The ester moiety in **4** connected C-3´ of the mannitol unit and the polyketide portion based on HMBC cross-peaks from H-3′ to the ester carbonyl C-1 (Ju et al., 2007). NOESY correlations between H_3_-25 and H-10, H_3_-27 and H-14, as well as between H_3_-29 and H-18 indicated *E*-configured olefins in **4**. The *erythro* configuration between H-2 and H-3 was deduced from their ^1^H-^1^H coupling constant (8.2 Hz). Therefore, the structure of muyocopronol B (**4**) was assigned.

Muyocopronol C (**5**) was isolated as yellowish white powders with [α]_D_ + 37 (c 0.2, MeOH) and assigned the molecular formula C_31_H_54_O_7_ following (−)-HRESIMS data analysis. Comparison of NMR and MS data between **4** and **5** suggested that the latter natural product was missing a mannitol unit. HMBC correlation from H-2 to a carbonyl carbon at δ_C_ 182.7, that had not been detected in the ^13^C NMR spectrum, confirmed the presence of a carboxylic acid moiety in **5** (Supplementary Figures S30, S33). Analysis of 2D NMR data further supported the polyketide structural part in **5**. The same relative configuration previously determined for **4** was also assigned for **5** following analysis of ^1^H − ^1^H coupling constants and the NOESY spectrum (Figure 9).

**Figure 10:**
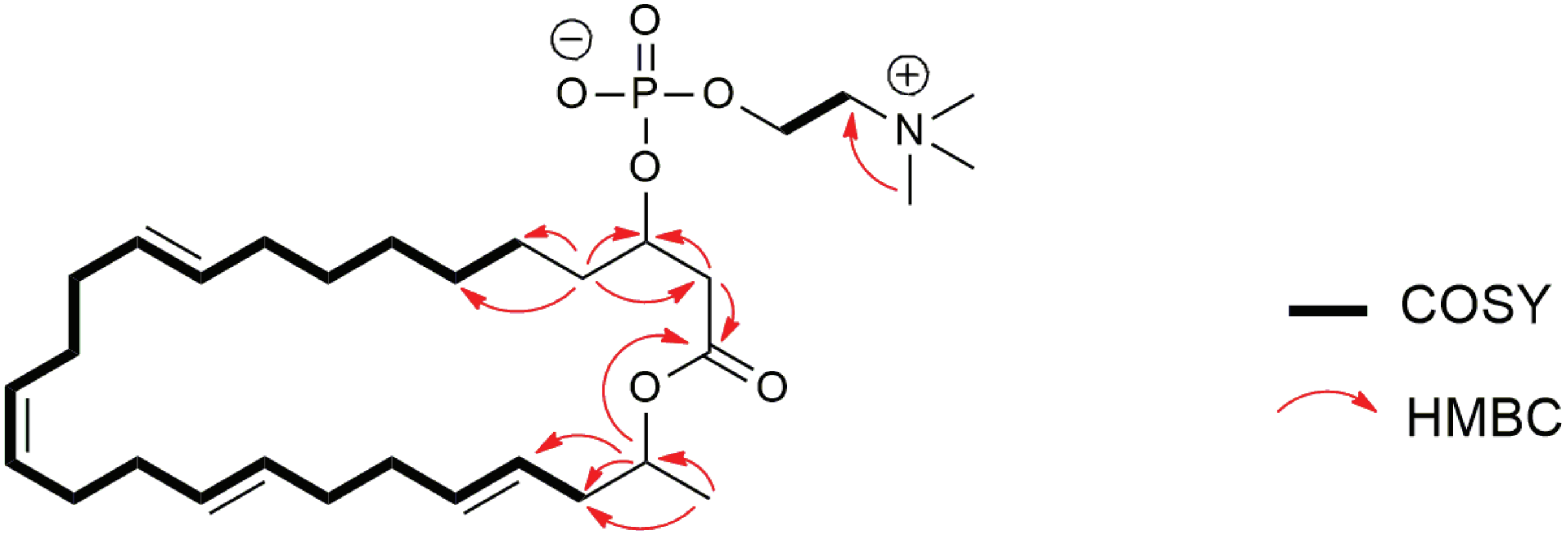
Selected COSY and HMBC correlations of tropicicolide (**6**).

Compound **6** (Figure 6) was isolated as pale-yellow sticky oil with [α]_D_ + 1.8 (c 1.2, MeOH) and assigned the molecular formula C_31_H_54_NO_6_P, following analysis of HRESIMS and NMR data. The ^1^H NMR spectrum of **6** (Table 4; Supplementary Figure S35) was similar to that reported for eushearilide, suggesting their structural resemblance (Hosoe et al., 2006; Tonoi et al., 2019). Comparison of ^13^C NMR (Supplementary Figure S36) and MS data of **6** to those of eushearilide suggested that **6** was a 26-membered macrolide containing four non-conjugated diene and a choline phosphate ester group. The presence of the choline phosphate group was readily indicated by the characteristic ^13^C/^1^H resonances at δ_C_/δ_H_ 54.7/3.22, 67.6/3.63, and 60.3/4.27. The positions of non-conjugated four double bonds in **6** were assigned based on ^13^C NMR chemical shift and HMBC data (Supplementary Figures S36, S39). Detailed analysis of 2D NMR data further supported the remaining structural assignment in **6** (Figure 10). However, attempts to address the configuration of the double bonds of **6** proved unsuccessful due to the overlapping ^1^H-NMR resonances of the olefins. Moreover, the stereochemical determination at C-3 and C-25 was unfeasible using spectroscopic method due to the rotatable nature of the methyl and the choline phosphate moieties. Thus, the planar structure of **6** was assigned and named tropicicolide (**6**).

**Table 4:** NMR spectral data of tropicicolide (**6**).

Position	6*^a^*	
	^13^C, mult. (*J* = Hz), type	^1^H, mult. (*J* = Hz)
1	171.9, C	
2	42.1, d (3.5), CH_2_	2.52, m, 2.81, m
3	74.1, d (5.9), CH	4.54, m
4	36.3, d, (5.4), CH_2_	1.65, m
5	25.4, CH_2_	1.40, m, 1.48, m
6	30.7, CH_2_	1.30, m, 1.39, m
7	29.5, CH_2_	1.36, m
8	29.6, CH_2_	1.36, m
9	33.1, CH_2_	1.99, m
10	132.0, CH	5.39, m
11	130.7, CH	5.39, m
12	33.3, CH_2_	1.99, m
13	33.4, CH_2_	1.99, m
14	131.9, CH	5.39, m
15	131.0, CH	5.39, m
16	33.5, CH_2_	2.06, m
17	33.5, CH_2_	2.06, m
18	131.9, CH	5.35, m
19	131.2, CH	5.37, m
20	33.6, CH_2_	2.06, m
21	33.8, CH_2_	2.06, m
22	134.5, CH	5.51, m
23	126.6, CH	5.39, m
24	39.9, CH_2_	2.25, m
25	72.4, CH	4.89, m
26	19.9, CH_3_	1.21, d (6.2)
27	60.3, d (5.1), CH_2_	4.27, m
28	67.6, CH_2_	3.63, m
N-(CH_3_)_3_	54.7, t (3.7), CH_3_	3.22, s

a ^1^H (400 MHz) and ^13^C (100 MHz) in methanol-*d*_4_.

Assignments based on COSY, HSQC, and HMBC and comparison with literature compound, eushearilide. Chemical shifts (δ) in ppm. s, singlet; d, doublet; t, triplet; and m, multiplet. One proton unless otherwise stated.

### Chemical Structural Data

The UV spectra, HRESIMS spectra, 1D and 2D NMR spectra of palmarumycin CP_30_ (**1**) and palmarumycin C_8_ (**2**), muyocopronols A (**3**), B (**4**), and C (**5**), and tropicicolide (**6**) are provided (Supplementary Figures S7–S41).

#### Palmarumycin CP_30_ (1)

Brownish amorphous solid; [α]_D_ + 130 (c 0.6, MeOH); UV (MeCN/H_2_O) *λ*_max_ (%) 225 (100%), 300 (15%) nm; (+)-HRESIMS: *m/z* 317.0816 [M + H − H_2_O]^+^ (calcd for C_20_H_13_O_4_, 317.0808); and ^1^H and ^13^C NMR data, see Table 2.

#### Palmarumycin C_8_ (2)

Brownish amorphous solid; [α]_D_ + 54 (c 1.2, MeOH); UV (MeCN/H_2_O) *λ*_max_ (%) 225 (100%), 300 (15%) nm; (+)-HRESIMS *m/z* 385.0473 [M + H]^+^ (calcd for C_20_H_14_ClO_6_, 385.0479); and ^1^H and ^13^C NMR data, see Table 2.

#### Muyocopronol A (3)

Brownish amorphous solid; [α]_D_ + 68 (c 0.2, MeOH); UV (MeCN/H_2_O) *λ*_max_ (%) 200 (100%), 225 (20%) nm; (−)-HRESIMS m/z 617.3915 [M − H]^−^ (calcd for C_32_H_57_O_11_, 617.3901); and ^1^H and ^13^C NMR data, see Table 3.

#### Muyocopronol B (4)

Yellowish white solid; [α]_D_ + 33 (c 0.8, MeOH); UV (MeCN/H_2_O) *λ*_max_ (%) 200 (100%), 225 (20%) nm; (−)-HRESIMS m/z 701.4475 [M − H]^−^ (calcd for C_37_H_65_O_12_, 701.4476); and ^1^H and ^13^C NMR data, see Table 3.

#### Muyocopronol C (5)

Yellowish white solid; [α]_D_ + 37 (c 0.2, MeOH); UV (MeCN/H_2_O) *λ*_max_ (%) 200 (100%), 225 (20%) nm; (−)-HRESIMS m/z 537.3811 [M − H]^−^ (calcd for C_31_H_53_O_7_, 537.3792); and ^1^H and ^13^C NMR data, see Table 3.

#### Tropicicolide (6)

Yellow sticky oil; [α]_D_ + 1.8 (c 1.2, MeOH); UV (MeCN/H_2_O) *λ*_max_ (%) 195 (100%), 225 (25%) nm; (+)-HRESIMS m/z 568.3764 [M + H]^+^ (calcd for C_31_H_55_NO_6_P, 568.3767); and ^1^H and ^13^C NMR data, see Table 4.

### Antimicrobial and Cytotoxic Activities of Six Compounds Isolated From the Three Fungal Strains

The newly discovered palmarumycin CP_30_ (**1**) and its known analogue, palmarumycin C_8_ (**2**) isolated from strain *Lophiotrema* sp. F6932, three new compounds, muyocopronol A-C (**3–5**) isolated from *M. laterale* F5912 and a novel compound, tropicicolide (**6**) discovered from *C. tropicicola* F10154 were subjected to antimicrobial and cytotoxic testing against a panel of three microbial pathogens; *S. aureus*, *C. albicans*, and *A. fumigatus* and three cancer cell lines; A549, MIA PaCa-2 and PANC-1. Table 5 gives a comparison of the bioactivity of the six isolated compounds and that of the reference standards. Supplementary Figure S42 shows the dose–response curves of the six compounds and their IC_90_ values for the bacterial pathogen and IC_50_ for the fungal pathogens and cancer cell lines. The inhibitory effects of standard antimicrobial agents, gentamicin and amphotericin B and cytotoxic agent puromycin against the tested microbial pathogens and cancer cell lines, respectively, are shown in Supplementary Figure S43. For the two fungal pathogens, tropicicolide (**6**) was the most potent compound with an IC_50_ of 1.8 μg/ml against *A. fumigatus* but with a lesser activity of an IC_50_ value of 7.1 μg/ml against the yeast pathogen *C. albicans*. Muyocopronol B (**4**) equally revealed promising antifungal activity with IC_50_ values of 3.4 and 4.5 μg/ml against the *C. albicans* and *A. fumigatus*, respectively. Palmarumycin C_8_ (**2**) revealed the best inhibitory activity against *S. aureus* with an IC_90_ value of 18 μg/ml, while the rest of the compounds had IC_90_ > 100 μg/ml against the bacterial pathogen. In addition, palmarumycin C_8_ (**2**) also possessed the best antiproliferative activities with IC_50_ values of 1.1 and 2.1 μg/ml against MIA PaCa-2 and PANC-1 cell lines, respectively (Table 5).

**Table 5:** A comparison of the bioactivity of the six isolated compounds with the reference standard compounds used in the study.

Compound	*S. aureus*	*C. albicans*	*A. fumigatus*	A549	MIA PaCa-2	PANC-1
	IC_90_ (μg/ml)	IC_50_ (μg/ml)				
Palmarumycin CP_30_	>100	>100	35	>100	20.1	31.7
Palmarumycin C_8_	18	58	7	10.7	1.1	2.1
Muyocopronol A	>100	99	26.3	97.4	96.4	97.6
Muyocopronol B	>100	4.5	3.4	44.5	40	77.1
Muyocopronol C	>100	9.2	>100	>100	>100	>100
Tropicicolide	>100	7.1	1.8	>100	>100	>100
Gentamicin	0.15	–		–	–	–
Amphotericin B	–	0.29	0.26	–	–	
Puromycin	–	–	–	0.31	0.18	0.18

## Discussion

Despite the emerging importance of fungal endophytes as sources of bioactive molecules, only a small fraction of the estimated 1 million fungal endophytes have been described (Strobel and Daisy, 2003). An even smaller number of fungal endophytes have been investigated for their secondary metabolite biosynthetic potential (Rajamanikyam et al., 2017). There is a great prospect that plants from the less studied and distinctive geographies may harbor unique fungal endophytes capable of producing molecules with interesting chemistry and of great therapeutic value. The current study aimed at investigating the diversity and bioactivity potential of fungal endophytes from the A*STAR Natural Product Library (NPL), originally isolated from various locations corresponding to four distinct habitats in Singapore. Fungal isolates were identified on the basis of sequence analysis of the internal transcribed spacer region (ITS2) of the rDNA sequence. Sequencing of the ITS region allows for rapid species-level molecular identification of fungi (Raja et al., 2017). As the most widely used fungal barcode marker, ITS is easily amplifiable *via* PCR and provides the highest prospect for accurate characterization of a wide range of fungal groupings in comparison with other rDNA region or protein genes (Chi et al., 2019). In the current study, ITS86F and ITS4 primer set was used for the amplification of the ITS2 of the rDNA gene. This primer set has been found to outperform a number of other primer pairs in a number of essential qualities in fungal identification such as high fungal specificity for numerous fungal clades, *in silico* primer efficiency, PCR specificity, and amplification efficiency among other traits, making it a primer pair of choice for fungal diversity studies (Vancov and Keen, 2009; De Beeck et al., 2014).

With the exception of the three fungal isolates, *Coprinopsis cinerea* F10390 and *Fomitopsis feei* F11601 (*Basidiomycota*) and *Rhizomucor variabilis* F10137 (*Mucoromycota*), all the identified fungal reads belong to the phylum *Ascomycota*. Numerous studies have reported that taxonomically, *Ascomycetes* are the most abundant representative of endophytic fungal communities with occasional isolation of *Basidiomycetes* and *Zygomycetes* (Rosa et al., 2011; Sun and Guo, 2012; Hamzah et al., 2018). Within the *Ascomycetes*, *Sordariomycetes*, and *Dothideomycetes* were the most dominant classes with four orders belonging to the former alone accounting for more than 72% of all the identified fungal strains. *Sordariomycetes* and *Dothideomycetes* have likewise been cited as among the most dominant classes of fungal endophytes in studies carried from a wide range of natural ecosystems (Rodriguez et al., 2009; Vaz et al., 2014; Wu et al., 2020; Khalmuratova et al., 2021). Moreover, even for studies focusing on the isolation of fungal endophytes from specific host plants, such as *Acanthus ilicifolius*, *Zanthoxylum bungeanum*, and *Rhizophora mucronata*, the two classes have been reported to constitute the highest number of recovered fungal isolates (Li et al., 2016; Hamzah et al., 2018; Chi et al., 2019).

The bulk of the identified fungal genera (73%) was found in only one of any of the four study habitats; an indication of high taxonomic variation of endophytic fungal community between the four studied habitats. The observed variations in fungal community composition may be a function of the ecological differences as well as a reflection of the environmental structuring of Singapore’s landscape and vegetation. These aspects have been found to have an influence on the distribution and diversity of the host plants and subsequently fungal endophyte composition (Zimmerman and Vitousek, 2012; Fang et al., 2019). The most abundant genus was *Colletotrichum*, which accounted for 27% of all the identified fungal reads. Members of *Colletotrichum* were found in Habitat 2, 3, and 4 but none from Habitat 1. Some of the most commonly occurring members of this genus belong to the *Colletotrichum gloeosporioides* species complex and include *C. asianum*, *C. fructicola*, *C. horii*, and *C. gloeosporioide*. Other identified strains from this genus *Colletotrichum* were, *C. magnisporum*, *C. karstic*, *C. simmondsii*, *C. dracaenophilum*, *C. scovillei*, *C. tropicicola*, and *C. siamense*. The genus *Colletotrichum* is widely distributed in nature and comprise of strains with diverse and variable lifestyles, including phytopathogens, endophytes, saprophytes hemibiotroph, and occasionally entomopathogens (Dean et al., 2012; da Silva et al., 2020). The genus comprises of more than 700 species and has been cited as among the most extensively distributed endophytic fungal Genera (Rabha et al., 2016). Furthermore, many members of *Colletotrichum* genus are known producers of a wide range of secondary metabolites and numerous classes of bioactive molecules such as sterols, pyrones, phenolics, terpenes, and fatty acids with diverse bioactivities have been isolated from members of this genus (Kim and Shim, 2019; Moraga et al., 2019). In the current study, among the active fungal strains, nine strains corresponding to 14% of all the active strains belong to the genus *Colletotrichum*.

Fungal endophytes are prolific producers of bioactive molecules including structurally uncommon molecules and those with remarkable biological activities (Nisa et al., 2015; Gupta and Shukla, 2020; Ortega et al., 2021). However, several limiting factors such as low yields of targeted compounds and weak expression of many of their biosynthetic gene clusters under standard laboratory growth conditions limits the realization of full biosynthetic potential of these microbial factories (González-Menéndez et al., 2018; Motoyama et al., 2021). To overcome such challenges, numerous strategies, such as co-culture, chemical elicitation, variation of culture growth conditions, and genetic engineering of strains of interest among other strategies have been employed (Baral et al., 2018; Kjærbølling et al., 2019; Gakuubi et al., 2021). In the current study, chemical elicitation using 5-azacytidine and SAHA and variation of fermentation media were assessed for their potential in enhancing secondary metabolite biosynthesis and bioactivity of crude fungal extracts. A general trend was observed whereby crude extractable yields were higher for fungal strains that were grown in the presence of the epigenetic modifiers in comparison with those obtained from strains grown without the chemical elicitors. Similar findings have been reported previously; in one study, it was revealed that addition of SAHA to the cultures of an endophytic fungi, *Botryosphaeria mamane* at the seed and production stage and at the production stage only resulted in an increase in the yield of crude extracts by 50 and 32%, respectively (Triastuti et al., 2019). Furthermore, growth of *B. mamane* in the presence of SAHA resulted in an induced biosynthesis of eight new metabolites that were not detected in the control cultures (Triastuti et al., 2019). The use of epigenetic modifiers, mainly histone deacetylase and DNA methyltransferases inhibitors, to perturb fungal biosynthetic machinery leading to enhanced bioactivity of the resultant extracts in addition to induced biosynthesis of new compounds, has been observed in a number of other studies (Magotra et al., 2017; Dwibedi et al., 2019; González-Menéndez et al., 2019). In the current study, enhanced antimicrobial and cytotoxic activities were observed in numerous elicitor-derived extracts. For example, crude extracts from strain F6932 when grown in CF02LB media in the presence of 5-azacytidine revealed strong antibacterial activity against *S. aureus*. Extracts from the same fungal strain grown in the same media in the absence of chemical elicitors and in the presence of SAHA did not show similar antibacterial activity. Furthermore, extracts of the same strain grown in CF18LB in the presence and absence of the two chemical elicitors similarly did not show any antibacterial activity. Since not all elicitor-derived extracts exhibited enhanced biological activities, the observed increase in bioactivity of extracts from certain fungal strains grown in the presence of chemical elicitors can only be attributed to differential expression of gene clusters responsible for biosynthesis of secondary metabolites as a result of chemical elicitation. For fungal strains grown in different fermentation media, generally, the weights of crude extracts were found to be higher for fungal strains grown in the solid media in comparison with those derived from liquid media cultures. Fungal strains grown in solid media have been found to produce crude extracts with masses that are one to two orders of magnitude higher than those derived from the same strains grown in liquid media (VanderMolen et al., 2013). Variation of media type and composition falls within the framework of the one strain many-compounds (OSMAC) approach (Bode et al., 2002). This method entails systematic manipulation of various growth conditions with an aim of identifying the growth regimes that enhances optimum quality, diversity, and novelty of bioactive secondary metabolites (Bills et al., 2008; VanderMolen et al., 2013). Thus, variation of media type and composition can be employed to enhance the yield of a target bioactive compound (García-Kirchner et al., 2005) or as an approach to maximize the chemical diversity from an individual strain or few selected strains (Elias et al., 2006; VanderMolen et al., 2013). The biological activity of F6932 and F5912, two of the three fungal strains that were progressed to compound isolation, were only uncovered following the use of chemical elicitation and growth of fungal strains in additional media, respectively. This means that neither the bioactivity of the two strains nor the four novel compounds isolated from them would have been identified had this study been limited to the used of two initial liquid media (CF02LB and CF18LB). These finding therefore demonstrated the usefulness of chemical elicitation and media diversification as avenues for enhancing the discovery of novel fungal secondary metabolites.

From the dereplication studies, extracts obtained from *Lophiotrema* sp. F6932, *M. laterale* F5912, and *C. tropicicola* F10154 were selected for further investigation because no known molecules were found that could explain the activity observed in three extracts. Chemical investigation of the extracts led to the identification of a number of new compounds, palmarumycin CP_30_ (**1**) and a known compound palmarumycin C_8_ (**2**) from *Lophiotrema* sp. F6932, three new polyketides, muyocopronols A (**3**), B (**4**), and C (**5**) from *M. laterale* F5912, and a new compound designated as tropicicolide (**6**) discovered from *C. tropicicola* F10154 (Figure 6). Palmarumycins belong to the spirobisnaphthalenes family of secondary metabolites characterized by the presence of a 1,8-dihydroxynaphthalene-derived spiroketal unit connected to a second oxidized naphthalene unit (Mou et al., 2015). Palmarumycins which have been shown to possess a broad range of biological activities including antibacterial, antifungal, and antitumor activities are produced mainly by filamentous fungi (Kanehara et al., 2021). These compounds have been isolated from filamentous fungi from a wide range of Genera. For example, nine palmarumycins, B_1_ − B_9_ were isolated from endophytic fungi *Berkleasmium* sp. with two more new analogues, palmarumycin C_12_ and C_13_ isolated from the same fungi (Shan et al., 2014; Mou et al., 2015). Palmarumycins CP_4_ and CP_5_ were isolated from *Phaeoseptum* sp. (Kanehara et al., 2021), while palmarumycin CE_4_, palmarumycin CP_4_, and palmarumycin CP_1_ were discovered from and endophytic fungus *Anteaglonium* sp. (Mafezoli et al., 2018). Muyocopronols A–C (**3**–**5**) are closely related to bionectriols, which are fungal secondary metabolite of polyketide origin and assign to a class of glycosylated, polyunsaturated polyols (Freinkman et al., 2009). The first of this type of molecules, designated bionectriol A, was isolated from *Bionectria* sp., associated with the fungus-growing ant *Apterostigma dentigerum* (Freinkman et al., 2009). Three related compounds (bionectriols B–D) were isolated from *Bionectria ochroleuca* and all exhibited strong anti-biofilm activity against *C. albicans* in addition to synergistic antifungal activities when used in combination with amphotericin B (Wang et al., 2014). Significantly, only these two references exist reporting the isolation of bionectriols from fungi and in both cases, these compounds were isolated from members of the *Bionectria* sp. which are within the class *Sordariomycetes*. This makes the discovery of these three new compounds from *M. laterale*, which belong to a different fungal class, i.e., *Dothideomycetes* an interesting finding. From *C. tropicicola*, tropicicolide (**6**) was discovered. The structure of tropicicolide (**6**) shares similarity with that of eushearilide. Eushearilide is a 20-membered macrolide with a non-conjugated diene and a choline phosphate ester moiety and was for the first time isolated from cultures of *Eupenicillium shearii* and exhibited antifungal activity against a wide range of human pathogens including *A. fumigatus, Aspergillus niger*, and *C. albicans* (Hosoe et al., 2006). In the current study, with the exception of palmarumycin C_8_ which presented an IC_90_ of 18 μg/ml, the rest of the isolated compounds were inactive against *S. aureus* (IC_90_ > 100 μg/ml). The antibacterial activity of palmarumycin C_8_, however, was very weak compared to that of the standard antibacterial compound gentamicin, which presented an IC_90_ valued of 0.15 μg/ml against *S. aureus* (Table 5). The newly discovered tropicicolide (**6**) had the most potent activity against *A. fumigatus* among all the isolated bioactive compounds with an IC_50_ of 1.8 μg/ml. However, this activity is weaker than that exhibited by the standard antifungal agent amphotericin B which produced an IC_50_ of 0.26 and 0.29 μg/ml against *A. fumigatus and C. albicans*, respectively (Table 5). In summary, none of the six isolated compounds exhibited better antimicrobial activities than the commercial compounds used as standards in the study. Since the discovery of eushearilide some years back from the *E. shearii*, only total synthesis of the compound and its various stereoisomers and/or related compounds have been reported (Tonoi et al., 2016, 2019). This, therefore, is the second time that this class of compounds is being reported from nature. Palmarumycin C_8_ exhibited the best antiproliferative activities with IC_50_ values of 1.1, 2.1, and 10.7 μg/ml against MIA PaCa-2, PANC-1, and A549 cells, respectively. However, these compounds were less cytotoxic compared to the standard compound, puromycin whose IC_50_ values against the three cell lines was 0.18, 0.18, and 0.31 μg/ml, respectively.

## Conclusion

In this study, the diversity, antimicrobial, and cytotoxic activities of fungal endophytes isolated from diverse habitats of Singapore were investigated. Our results reveal that Singapore harbors a diverse community of fungal endophytes including potentially new and undescribed strains that warrant further investigation. We observed the dominant presence of *Ascomycetes* with members of *Sordariomycetes* and *Dothideomycetes* classes accounting for more than 90% of all the fungal strains belonging to this phylum. A total of 63 fungal strains exhibiting antimicrobial and/or cytotoxic activity against at least one of the tested panel of pathogens and cancer cells lines were identified, with member of the order *Diaporthales* accounting for the highest number of active fungal strains. Three fungal strains were progressed to large-scale fermentation for compound isolation and structural elucidation resulting in the discovery of five novel compounds and one known compound. Significantly, antimicrobial hits from the crude extracts derived from two of the three prioritized fungal strains, *Lophiotrema* sp. F6932 and *M. laterale* F5912, were uncovered following their growth in the presence of 5-azacytidine and three additional media, respectively. Consequently, had our investigation on the discovery of bioactive secondary metabolites from fungal endophytes been limited to the use of two initial liquid media, four of the five newly discovered compounds associated with the two aforementioned strains would not have been discovered. This study therefore demonstrates how diversification of growth media and the use of selected chemical epigenetic modifiers can facilitate the discovery of new bioactive microbial natural products.

## Data Availability Statement

The data presented in the study are deposited in the National Center for Biotechnology Information (NCBI) database GenBank, accession number OM791857-OM792078.

## Author Contributions

SN and MG conceptualized and planned the study. MG performed the biological studies. MM and Z-XL assisted with troubleshooting. KC, MW, and YK performed the chemical analysis of bioactive extracts, compound isolation, and structure elucidation. All authors contributed to the article and approved the submitted version.

## Funding

This research was funded by the Singapore Institute of Food and Biotechnology Innovation, Agency for Science, Technology and Research (A*STAR), Singapore.

## Conflict of Interest

The authors declare that the research was conducted in the absence of any commercial or financial relationships that could be construed as a potential conflict of interest.

## Publisher’s Note

All claims expressed in this article are solely those of the authors and do not necessarily represent those of their affiliated organizations, or those of the publisher, the editors and the reviewers. Any product that may be evaluated in this article, or claim that may be made by its manufacturer, is not guaranteed or endorsed by the publisher.

## Footnotes

^1^ www.base-asia.com

^2^ https://benchling.com

^3^ http://dnp.chemnetbase.com
