# Supplementary material for: Enhancing the Discovery of Bioactive Secondary Metabolites From Fungal Endophytes Using Chemical Elicitation and Variation of Fermentation Media
Source: Front Microbiol. 2022 Jun 6;13:898976. doi: 10.3389/fmicb.2022.898976 (PMC9207341; doi:10.3389/fmicb.2022.898976)
Supplement: Supplementary file 1 [file Data_Sheet_1.docx]

Supplementary Material

**Table S1.** Identified endophytic fungal isolates from the four habitats in Singapore: Habitat 1 (Upper Seletar, Upper Pierce and MacRitchie reservoirs), Habitat 2 (Cluny Road), Habitat 3 (Pulau Ubin and St. John’s islands), Habitat 4 (Bukit Timah Nature Reserve and Kent Ridge park).

| **#** | **Fungal isolate*** | **Habitat** | **Substrate/ plant tissue** | **Closest Relative in NCBI**  **(Accession No.)** | **Percent Identity** | **Accession Number** | **Phylum, Class, Order** |
| --- | --- | --- | --- | --- | --- | --- | --- |
| 1 | F2681 | Habitat 1 | Flowers | *Lasiodiplodia theobromae* (MT644474.1) | 100.0 | OM791857 | *Ascomycota, Dothideomycetes, Botryosphaeriales* |
| 2 | F4434 | Habitat 1 | Leaves | *Aspergillus assiutensis* (MT640286.1) | 99.7 | OM791858 | *Ascomycota, Eurotiomycetes, Eurotiales* |
| 3 | F4440 | Habitat 1 | Leaves | *Neopestalotiopsis saprophytica* (MT576586.1) | 99.7 | OM791859 | *Ascomycota, Sordariomycetes, Xylariales* |
| 4 | F2682 | Habitat 1 | Flowers | *Aspergillus flavus* (MT497451.1) | 99.7 | OM791860 | *Ascomycota, Eurotiomycetes, Eurotiales* |
| 5 | F4448 | Habitat 1 | Leaves | *Aspergillus assiutensis* (MT640286.1) | 99.7 | OM791861 | *Ascomycota, Eurotiomycetes, Eurotiales* |
| 6 | F4451 | Habitat 1 | Leaves | *Phomopsis* sp. (DQ780437.1) | 99.7 | OM791862 | *Ascomycota, Sordariomycetes, Diaporthales* |
| 7 | F4452 | Habitat 1 | Leaves | *Trichoderma* sp. (MT557551.1) | 100.0 | OM791863 | *Ascomycota, Sordariomycetes, Hypocreales* |
| 8 | F4437 | Habitat 1 | Leaves | *Coniella heterospora* (LT800501.1) | 96.8 | OM791864 | *Ascomycota, Sordariomycetes, Diaporthales* |
| 9 | F10318 | Habitat 1 | Leaves | *Bartalinia* sp. (MH482848.1) | 100.0 | OM791865 | *Ascomycota, Sordariomycetes, Xylariales* |
| 10 | F10339 | Habitat 1 | Leaves | *Chaetomium* sp. (MF495440.1) | 99.6 | OM791866 | *Ascomycota, Sordariomycetes, Sordariales* |
| 11 | F12134 | Habitat 1 | Plant samples | *Aspergillus assiutensis* (MT640286.1) | 99.7 | OM791867 | *Ascomycota, Eurotiomycetes, Eurotiales* |
| 12 | F10670 | Habitat 1 | Leaves | *Chrysoporthe* sp. (CP064907.1) | 99.7 | OM791868 | *Ascomycota, Sordariomycetes, Diaporthales* |
| 13 | F4447 | Habitat 1 | Leaves | *Neopestalotiopsis saprophytica* (MT576586.1) | 99.7 | OM791869 | *Ascomycota, Sordariomycetes, Xylariales* |
| 14 | F4445 | Habitat 1 | Leaves | *Phomopsis* sp. (GU066685.1) | 100.0 | OM791870 | *Ascomycota, Sordariomycetes, Diaporthales* |
| 15 | F4442 | Habitat 1 | Leaves | *Pestalotiopsis* sp. (MT075872.1) | 99.3 | OM791871 | *Ascomycota, Sordariomycetes, Xylariales* |
| 16 | F10429 | Habitat 1 | Leaves | *Aspergillus assiutensis* (MT640286.1) | 99.7 | OM791872 | *Ascomycota, Eurotiomycetes, Eurotiales* |
| 17 | F4446 | Habitat 1 | Leaves | *Pestalotiopsis* sp. (KX960814.1) | 100.0 | OM791873 | *Ascomycota, Sordariomycetes, Xylariales* |
| 18 | F12124 | Habitat 1 | Plant samples | *Humicola fuscoatra* (MT557193.1) | 99.7 | OM791874 | *Ascomycota, Sordariomycetes, Sordariales* |
| 19 | F4431 | Habitat 1 | Leaves | *Neopestalotiopsis clavispora* (MG386209.1) | 100.0 | OM791875 | *Ascomycota, Sordariomycetes, Xylariales* |
| 20 | F10338 | Habitat 1 | Leaves | *Aspergillus flavus* (MT626059.1) | 99.3 | OM791876 | *Ascomycota, Eurotiomycetes, Eurotiales* |
| 21 | F11599 | Habitat 1 | Basidiomycetes | *Aspergillus assiutensis* (MT640286.1) | 99.0 | OM791877 | *Ascomycota, Eurotiomycetes, Eurotiales* |
| 22 | F3084 | Habitat 1 | Stems | *Talaromyces* sp. (MN543991.1) | 100.0 | OM791878 | *Ascomycota, Eurotiomycetes, Eurotiales* |
| 23 | F10764 | Habitat 1 | Leaves | *Corynespora cassiicola* (MN393243.1) | 100.0 | OM791879 | *Ascomycota, Dothideomycetes, Pleosporales* |
| 24 | F10132 | Habitat 2 | Flowers | *Neopestalotiopsis clavispora* (MN519192.1) | 100.0 | OM791914 | *Ascomycota, Sordariomycetes, Xylariales* |
| 25 | F10133 | Habitat 2 | Flowers | *Diaporthe phaseolorum* (MT043765.1) | 100.0 | OM791915 | *Ascomycota, Sordariomycetes, Diaporthales* |
| 26 | F10134 | Habitat 2 | Flowers | *Fusarium petroliphilum* (KY436196.1) | 99.3 | OM791916 | *Ascomycota, Sordariomycetes, Hypocreales* |
| 27 | F10136 | Habitat 2 | Flowers | *Fusarium proliferatum* (MT476359.1) | 100.0 | OM791917 | *Ascomycota, Sordariomycetes, Hypocreales* |
| 28 | F10137 | Habitat 2 | Flowers | *Rhizomucor variabilis* (HM639971.1) | 99.4 | OM791918 | *Mucoromycota, Zygomycetes, Mucorales* |
| 29 | F10144 | Habitat 2 | Flowers | *Colletotrichum asianum* (MT350261.1) | 100.0 | OM791919 | *Ascomycota, Sordariomycetes, Glomerellales* |
| 30 | F10145 | Habitat 2 | Leaves | *Phaeoacremonium* sp. (MK120896.1) | 99.3 | OM791920 | *Ascomycota, Sordariomycetes, Xylariales* |
| 31 | F10146 | Habitat 2 | Flowers | *Colletotrichum* sp. (KM510463.1) | 100.0 | OM791921 | *Ascomycota, Sordariomycetes, Glomerellales* |
| 32 | F10148 | Habitat 2 | Flowers | *Gliocephalotrichum bulbilium* (MN326476.1) | 100.0 | OM791922 | *Ascomycota, Sordariomycetes, Hypocreales* |
| 33 | F10149 | Habitat 2 | Leaves | *Cytospora rhizophorae* (JN083839.1) | [99.3](https://www.ncbi.nlm.nih.gov/nucleotide/JN083838.1?report=genbank&log$=nucltop&blast_rank=3&RID=JBTBE69701N) | OM791923 | *Ascomycota, Sordariomycetes, Diaporthales* |
| 34 | F10153 | Habitat 2 | Leaves | *Colletotrichum gloeosporioides* (MT043801.1) | 100.0 | OM791924 | *Ascomycota, Sordariomycetes, Glomerellales* |
| 35 | F10154 | Habitat 2 | Leaves | *Colletotrichum tropicicola* (MN826680.1) | 99.7 | OM791925 | *Ascomycota, Sordariomycetes, Glomerellales* |
| 36 | F10156 | Habitat 2 | Leaves | *Colletotrichum* sp. (MT577046.1) | 100.0 | OM791926 | *Ascomycota, Sordariomycetes, Glomerellales* |
| 37 | F10163 | Habitat 2 | Leaves | *Phialemoniopsis curvata* (MN511348.1) | 100.0 | OM791927 | *Ascomycota, Sordariomycetes, Xylariales* |
| 38 | F5912 | Habitat 2 | Leaves | *Muyocopron laterale* (NR_164055.1) | 99.7 | OM791928 | *Ascomycota, Dothideomycetes, Muyocopronales* |
| 39 | F5924 | Habitat 2 | Leaves | *Daldinia eschscholtzii* (MT065673.1) | 100.0 | OM791929 | *Ascomycota, Sordariomycetes, Xylariales* |
| 40 | F5990 | Habitat 2 | Leaves | *Colletotrichum fructicola* (MT424894.1) | 99.3 | OM791930 | *Ascomycota, Sordariomycetes, Glomerellales* |
| 41 | F6133 | Habitat 2 | Stems | [*Phomopsis* sp. (MK587755.1)](https://www.ncbi.nlm.nih.gov/nucleotide/MK587755.1?report=genbank&log$=nucltop&blast_rank=1&RID=JBTBE69701N) | 100.0 | OM791931 | *Ascomycota, Sordariomycetes, Diaporthales* |
| 42 | F5920 | Habitat 2 | Leaves | *Hypoxylon fendleri* (KY173350.1) | 99.3 | OM791933 | *Ascomycota, Sordariomycetes, Xylariales* |
| 43 | F4873 | Habitat 2 | Leaves | *Arthrinium hydei* (NR_121557.1) | 100.0 | OM791934 | *Ascomycota, Sordariomycetes, Xylariales* |
| 44 | F5838 | Habitat 2 | Leaves | *Phomopsis* sp. (MK587755.1) | 100.0 | OM791935 | *Ascomycota, Sordariomycetes, Diaporthales* |
| 45 | F4872 | Habitat 2 | Leaves | *Nigrospora sphaerica* (KX778679.1) | 99.7 | OM791936 | *Ascomycota, Sordariomycetes, Xylariales* |
| 46 | F6006 | Habitat 2 | Leaves | *Colletotrichum karsti* (MN273072.1) | 99.3 | OM791937 | *Ascomycota, Sordariomycetes, Glomerellales* |
| 47 | F5914 | Habitat 2 | Leaves | *Diaporthe phaseolorum* (MH930433.1) | 94.1 | OM791938 | *Ascomycota, Sordariomycetes, Diaporthales* |
| 48 | F6062 | Habitat 2 | Stems | *Phomopsis* sp. (AB505410.1) | 99.3 | OM791939 | *Ascomycota, Sordariomycetes, Diaporthales* |
| 49 | F6068 | Habitat 2 | Leaves | *Diaporthe* sp. (MT355680.1) | 100.0 | OM791940 | *Ascomycota, Sordariomycetes, Diaporthales* |
| 50 | F6069 | Habitat 2 | Leaves | *Phomopsis* sp. (MK587755.1) | 100.0 | OM791941 | *Ascomycota, Sordariomycetes, Diaporthales* |
| 51 | F6125 | Habitat 2 | Leaves | *Diaporthe searlei* (NR_168241.1) | 100.0 | OM791942 | *Ascomycota, Sordariomycetes, Diaporthales* |
| 52 | F10032 | Habitat 2 | Leaves | *Graphium* sp. (FJ946489.1) | 100.0 | OM791943 | *Ascomycota, Sordariomycetes, Hypocreales* |
| 53 | F9801 | Habitat 2 | Stems | *Talaromyces wortmannii* (KF984828.1) | 99.4 | OM791944 | *Ascomycota, Eurotiomycetes, Eurotiales* |
| 54 | F9798 | Habitat 2 | Fruits | *Melanoctona tectonae* (MK952330.1) | 91.7 | OM791945 | *Ascomycota, Eurotiomycetes, Chaetothyriales* |
| 55 | F9803 | Habitat 2 | Stems | *Colletotrichum dracaenophilum* (DQ286208.1) | 99.7 | OM791946 | *Ascomycota, Sordariomycetes, Glomerellales* |
| 56 | F9805 | Habitat 2 | Fruits | *Colletotrichum simmondsii* (MK541034.1) | 93.8 | OM791947 | *Ascomycota, Sordariomycetes, Glomerellales* |
| 57 | F9916 | Habitat 2 | Leaves | *Cladosporium dominicanum* (KY827344.1) | 99.7 | OM791948 | *Ascomycota, Sordariomycetes, Glomerellales* |
| 58 | F10164 | Habitat 2 | Leaves | *Muyocopron laterale* (NR_164055.1) | 97.6 | OM791949 | *Ascomycota, Dothideomycetes, Muyocopronales* |
| 59 | F9779 | Habitat 2 | Stems | *Phialemonium curvatum* (GU219470.1) | 99.7 | OM791950 | *Ascomycota, Sordariomycetes, Xylariales* |
| 60 | F9792 | Habitat 2 | Fruits | *Cylindromonium eugeniicola* (NR_166338.1) | 93.1 | OM791951 | *Ascomycota, Sordariomycetes, Hypocreales* |
| 61 | F6122 | Habitat 2 | Leaves | *Daldinia eschscholtzii* (MT065673.1) | 100.0 | OM791953 | *Ascomycota, Sordariomycetes, Xylariales* |
| 62 | F5919 | Habitat 2 | Leaves | *Xylaria* sp. (MN701035.1) | 100.0 | OM791954 | *Ascomycota, Sordariomycetes, Xylariales* |
| 63 | F6361 | Habitat 2 | Leaves | *Metarhizium anisopliae* (MK955481.1) | 100.0 | OM791956 | *Ascomycota, Sordariomycetes, Hypocreales* |
| 64 | F10130 | Habitat 2 | Flowers | *Sclerotinia trifoliorum* (KT970795.1) | 97.8 | OM791958 | *Ascomycota, Leotiomycetes, Helotiales* |
| 65 | F9917 | Habitat 2 | Leaves | *Lecanicillium testudineum* (MT512660.1) | 100.0 | OM791959 | *Ascomycota, Sordariomycetes, Hypocreales* |
| 66 | F10161 | Habitat 2 | Leaves | *Myriangium* sp. (EF464585.1) | 90.2 | OM791960 | *Ascomycota, Dothideomycetes, Myriangiales* |
| 67 | F5906 | Habitat 2 | Leaves | *Colletotrichum asianum* (MT350261.1) | 96.5 | OM791961 | *Ascomycota, Sordariomycetes, Glomerellales* |
| 68 | F5899 | Habitat 2 | Leaves | *Phomopsis* sp. (MN856266.1) | 98.3 | OM791962 | *Ascomycota, Sordariomycetes, Diaporthales* |
| 69 | F5857 | Habitat 2 | Leaves | *Hypoxylon monticulosum* (MN427954.1) | 99.7 | OM791963 | *Ascomycota, Sordariomycetes, Xylariales* |
| 70 | F5861 | Habitat 2 | Leaves | *Daldinia eschscholtzii* (MT065673.1) | 100.0 | OM791964 | *Ascomycota, Sordariomycetes, Xylariales* |
| 71 | F4655 | Habitat 2 | Leaves | *Colletotrichum gloeosporioides* (MT043801.1) | 100.0 | OM791965 | *Ascomycota, Sordariomycetes, Glomerellales* |
| 72 | F5845 | Habitat 2 | Leaves | *Pestalotiopsis microspora* (MT075872.1) | 100.0 | OM791966 | *Ascomycota, Sordariomycetes, Xylariales* |
| 73 | F5852 | Habitat 2 | Leaves | *Colletotrichum siamense* (KY646464.1) | 100.0 | OM791967 | *Ascomycota, Sordariomycetes, Glomerellales* |
| 74 | F5915 | Habitat 2 | Leaves | *Colletotrichum fructicola* (MT424894.1) | 100.0 | OM791968 | *Ascomycota, Sordariomycetes, Glomerellales* |
| 75 | F5935 | Habitat 2 | Leaves | *Colletotrichum gloeosporioides* (KC010546.1) | 99.3 | OM791969 | *Ascomycota, Sordariomycetes, Glomerellales* |
| 76 | F6142 | Habitat 2 | Leaves | *Colletotrichum gloeosporioides* (MT416209.1) | 99.3 | OM791970 | *Ascomycota, Sordariomycetes, Glomerellales* |
| 77 | F6358 | Habitat 2 | Leaves | *Colletotrichum asianum* (MT350261.1) | 100.0 | OM791971 | *Ascomycota, Sordariomycetes, Glomerellales* |
| 78 | F4657 | Habitat 2 | Leaves | *Aspergillus nomius* (MK192017.1) | 98.7 | OM791974 | *Ascomycota, Eurotiomycetes, Eurotiales* |
| 79 | F4876 | Habitat 2 | Leaves | *Neopestalotiopsis saprophytica* (MT576586.1) | 100.0 | OM791975 | *Ascomycota, Sordariomycetes, Xylariales* |
| 80 | F6139 | Habitat 2 | Leaves | *Paratubakia subglobosa* (NR_161043.2) | 91.7 | OM791976 | *Ascomycota, Sordariomycetes, Diaporthales* |
| 81 | F9794 | Habitat 2 | Fruits | *Soloacrosporiella acaciae* (NR_137986.1) | 95.0 | OM791977 | *Ascomycota, Dothideomycetes, incertae sedis* |
| 82 | F5910 | Habitat 2 | Leaves | *Diaporth*e sp. (MT495430.1) | 100.0 | OM791978 | *Ascomycota, Sordariomycetes, Diaporthales* |
| 83 | F5850 | Habitat 2 | Leaves | *Colletotrichum asianum* (MT350261.1) | 100.0 | OM791979 | *Ascomycota, Sordariomycetes, Glomerellales* |
| 84 | F4659 | Habitat 2 | Leaves | *Lecanicillium testudineum* (MT512660.1) | 100.0 | OM791980 | *Ascomycota, Sordariomycetes, Hypocreales* |
| 85 | F5864 | Habitat 2 | Leaves | *Lecanicillium testudineum* (MT512660.1) | 100.0 | OM791981 | *Ascomycota, Sordariomycetes, Hypocreales* |
| 86 | F5926 | Habitat 2 | Stems | *Cophinforma atrovirens* (MF436146.1) | 99.0 | OM791982 | *Ascomycota, Dothideomycetes, Botryosphaeriales* |
| 87 | F5930 | Habitat 2 | Stems | *Hypoxylon* sp. (KU683912.1) | 97.9 | OM791983 | *Ascomycota, Sordariomycetes, Xylariales* |
| 88 | F5931 | Habitat 2 | Stems | *Fusarium verticillioides* (MT598827.1) | 100.0 | OM791984 | *Ascomycota, Sordariomycetes, Hypocreales* |
| 89 | F5998 | Habitat 2 | Leaves | *Diaporthe phaseolorum* (MT043770.1) | 100.0 | OM791985 | *Ascomycota, Sordariomycetes, Diaporthales* |
| 90 | F6003 | Habitat 2 | Stems | *Diaporthe phaseolorum* (MT043765.1) | 100.0 | OM791986 | *Ascomycota, Sordariomycetes, Diaporthales* |
| 91 | F6053 | Habitat 2 | Leaves | *Phyllosticta capitalensis* (MK243489.1) | 94.2 | OM791987 | *Ascomycota, Dothideomycetes, Botryosphaeriales* |
| 92 | F6055 | Habitat 2 | Stems | *Phialemoniopsis ocularis* (MH045585.1) | 100.0 | OM791988 | *Ascomycota, Sordariomycetes, Xylariales* |
| 93 | F6059 | Habitat 2 | Stems | *Phomopsis* sp. (KX655604.1) | 100.0 | OM791989 | *Ascomycota, Sordariomycetes, Diaporthales* |
| 94 | F6066 | Habitat 2 | Leaves | *Phomopsis* sp. (DQ780437.1) | 99.7 | OM791990 | *Ascomycota, Sordariomycetes, Diaporthales* |
| 95 | F6071 | Habitat 2 | Stems | *Hypoxylon investiens* (KY828945.1) | 99.3 | OM791991 | *Ascomycota, Sordariomycetes, Xylariales* |
| 96 | F6076 | Habitat 2 | Leaves | *Phialemoniopsis ocularis* (MH045585.1) | 100.0 | OM791992 | *Ascomycota, Sordariomycetes, Xylariales* |
| 97 | F6120 | Habitat 2 | Leaves | *Colletotrichum asianum* (MT350261.1) | 100.0 | OM791993 | *Ascomycota, Sordariomycetes, Glomerellales* |
| 98 | F6121 | Habitat 2 | Leaves | *Colletotrichum asianum* (MT350261.1) | 100.0 | OM791994 | *Ascomycota, Sordariomycetes, Glomerellales* |
| 99 | F6123 | Habitat 2 | Leaves | *Phomopsis phyllanthicola* (MH930424.1) | 99.7 | OM791995 | *Ascomycota, Sordariomycetes, Diaporthales* |
| 100 | F6124 | Habitat 2 | Leaves | *Phomopsis phyllanthicola* (MH930424.1) | 99.0 | OM791996 | *Ascomycota, Sordariomycetes, Diaporthales* |
| 101 | F6127 | Habitat 2 | Leaves | *Lecythophora* sp. (MG250451.1) | 90.7 | OM791997 | *Ascomycota, Sordariomycetes, Coniochaetales* |
| 102 | F6129 | Habitat 2 | Stems | *Phomopsis* sp. (AB505410.1) | 99.7 | OM791998 | *Ascomycota, Sordariomycetes, Diaporthales* |
| 103 | F6131 | Habitat 2 | Leaves | *Phomopsis* sp. (AB505410.1) | 99.3 | OM791999 | *Ascomycota, Sordariomycetes, Diaporthales* |
| 104 | F6132 | Habitat 2 | Leaves | *Diaporthe* sp. (MT355680.1) | 100.0 | OM792000 | *Ascomycota, Sordariomycetes, Diaporthales* |
| 105 | F6136 | Habitat 2 | Leaves | *Phyllosticta* sp. (MH393348.1) | 99.3 | OM792001 | *Ascomycota, Dothideomycetes, Botryosphaeriales* |
| 106 | F6140 | Habitat 2 | Leaves | *Paratubakia subglobosa* (NR_161043.1) | 93.9 | OM792002 | *Ascomycota, Sordariomycetes, Diaporthales* |
| 107 | F6141 | Habitat 2 | Leaves | *Paratubakia subglobosa* (NR_161043.1) | 93.9 | OM792003 | *Ascomycota, Sordariomycetes, Diaporthales* |
| 108 | F6143 | Habitat 2 | Leaves | *Colletotrichum asianum* (MT350261.1) | 100.0 | OM792004 | *Ascomycota, Sordariomycetes, Glomerellales* |
| 109 | F6144 | Habitat 2 | Stems | *Phomopsis* sp. (MK587755.1) | 100.0 | OM792005 | *Ascomycota, Sordariomycetes, Diaporthales* |
| 110 | F6360 | Habitat 2 | Stems | *Diaporthe searlei* (NR_168241.1) | 100.0 | OM792006 | *Ascomycota, Sordariomycetes, Diaporthales* |
| 111 | F6341 | Habitat 2 | Leaves | *Phomopsis* sp. (AB505410.1) | 99.3 | OM792007 | *Ascomycota, Sordariomycetes, Diaporthales* |
| 112 | F10129 | Habitat 2 | Flowers | *Phomopsis asparagi* (MT459267.1) | 97.6 | OM791913 | *Ascomycota, Sordariomycetes, Diaporthales* |
| 113 | F6702 | Habitat 3 | Stems | *Colletotrichum fructicola* (MT424894.1) | 99.0 | OM791880 | *Ascomycota, Sordariomycetes, Glomerellales* |
| 114 | F6703 | Habitat 3 | Stems | *Colletotrichum asianum* (MT350261.1) | 100.0 | OM791881 | *Ascomycota, Sordariomycetes, Glomerellales* |
| 115 | F6704 | Habitat 3 | Fruits | *Colletotrichum fructicola* (MT424894.1) | 99.0 | OM791882 | *Ascomycota, Sordariomycetes, Glomerellales* |
| 116 | F6709 | Habitat 3 | Leaves | *Colletotrichum* sp. (MT577046.1) | 100.0 | OM791883 | *Ascomycota, Sordariomycetes, Glomerellales* |
| 117 | F6712 | Habitat 3 | Stems | *Colletotrichum fructicola* (MT424894.1) | 99.3 | OM791884 | *Ascomycota, Sordariomycetes, Glomerellales* |
| 118 | F6713 | Habitat 3 | Stems | *Phomopsis* sp. (MN856266.1) | 98.3 | OM791885 | *Ascomycota, Sordariomycetes, Diaporthales* |
| 119 | F6714 | Habitat 3 | Leaves | *Phomopsis* sp. (DQ780437.1) | 99.3 | OM791886 | *Ascomycota, Sordariomycetes, Diaporthales* |
| 120 | F6715 | Habitat 3 | Leaves | *Pestalotiopsis cocculi* (MT000051.1) | 99.7 | OM791887 | *Ascomycota, Sordariomycetes, Xylariales* |
| 121 | F6716 | Habitat 3 | Leaves | *Phomopsi*s sp. (MN099444.1) | 100.0 | OM791888 | *Ascomycota, Sordariomycetes, Diaporthales* |
| 122 | F6720 | Habitat 3 | Leaves | *Colletotrichum asianum* (MT350261.1) | 99.3 | OM791889 | *Ascomycota, Sordariomycetes, Glomerellales* |
| 123 | F6728 | Habitat 3 | Stems | *Phomopsis* sp. (MH472614.1) | 99.6 | OM791890 | *Ascomycota, Sordariomycetes, Diaporthales* |
| 124 | F6730 | Habitat 3 | Stems | *Diaporthe* sp. (MT495430.1) | 99.7 | OM791891 | *Ascomycota, Sordariomycetes, Diaporthales* |
| 125 | F6731 | Habitat 3 | Stems | *Phomopsis* sp. (KX655604.1) | 99.6 | OM791892 | *Ascomycota, Sordariomycetes, Diaporthales* |
| 126 | F6733 | Habitat 3 | Leaves | *Mycosphaerella* sp. (KX258811.1) | 99.3 | OM791893 | *Ascomycota, Dothideomycetes, Mycosphaerellales* |
| 127 | F6734 | Habitat 3 | Leaves | *Colletotrichum* sp. (MT570096.1) | 99.0 | OM791894 | *Ascomycota, Sordariomycetes, Glomerellales* |
| 128 | F6738 | Habitat 3 | Stems | *Colletotrichum fructicola* (KC845282.1) | 98.7 | OM791895 | *Ascomycota, Sordariomycetes, Glomerellales* |
| 129 | F6743 | Habitat 3 | Stems | *Diaporthe searlei* (NR_168241.1) | 99.7 | OM791896 | *Ascomycota, Sordariomycetes, Diaporthales* |
| 130 | F6910 | Habitat 3 | Leaves | *Mycosphaerella* sp. (KX258811.1) | 99.3 | OM791897 | *Ascomycota, Dothideomycetes, Mycosphaerellales* |
| 131 | F6914 | Habitat 3 | Leaves | *Mycosphaerella* sp. (KX258811.1) | 99.3 | OM791898 | *Ascomycota, Dothideomycetes, Mycosphaerellales* |
| 132 | F6923 | Habitat 3 | Stems | *Colletotrichum* sp. (MT570096.1) | 99.0 | OM791899 | *Ascomycota, Sordariomycetes, Glomerellales* |
| 133 | F6929 | Habitat 3 | Stems | *Fusarium solani* (MN637841.1) | 100.0 | OM791900 | *Ascomycota, Sordariomycetes, Hypocreales* |
| 134 | F6930 | Habitat 3 | Leaves | *Mycosphaerella* sp. (KX258811.1) | 99.3 | OM791901 | *Ascomycota, Dothideomycetes, Mycosphaerellales* |
| 135 | F6931 | Habitat 3 | Leaves | *Mycosphaerella* sp. (KX258811.1) | 100.0 | OM791902 | *Ascomycota, Dothideomycetes, Mycosphaerellales* |
| 136 | F6928 | Habitat 3 | Leaves | *Xylaria* sp. (LC424444.1) | 97.2 | OM791903 | *Ascomycota, Sordariomycetes, Xylariales* |
| 137 | F6932 | Habitat 3 | Stems | *Lophiotrema* sp. (MK587671.1) | 99.0 | OM791904 | *Ascomycota, Dothideomycetes, Pleosporales* |
| 138 | F6732 | Habitat 3 | Leaves | *Leptosphaerulina* sp. (MF405203.1) | 96.6 | OM791905 | *Ascomycota, Dothideomycetes, Pleosporales* |
| 139 | F6913 | Habitat 3 | Stems | *Leptosphaerulina* sp. (KY827352.1) | 99.7 | OM791906 | *Ascomycota, Dothideomycetes, Pleosporales* |
| 140 | F6915 | Habitat 3 | Leaves | *Mycosphaerella* sp. (KX258811.1) | 99.6 | OM791907 | *Ascomycota, Dothideomycetes, Mycosphaerellales* |
| 141 | F6916 | Habitat 3 | Leaves | *Acremonium hennebertii* (MN637805.1) | 98.2 | OM791908 | *Ascomycota, Sordariomycetes, Hypocreales* |
| 142 | F6917 | Habitat 3 | Leaves | *Leptosphaerulina* sp. (JN850998.1) | 96.3 | OM791909 | *Ascomycota, Dothideomycetes, Pleosporales* |
| 143 | F6717 | Habitat 3 | Leaves | *Phyllosticta capitalensis* (MT568601.1) | 99.3 | OM791910 | *Ascomycota, Dothideomycetes, Botryosphaeriales* |
| 144 | F6705 | Habitat 3 | Leaves | *Phyllosticta* sp. (MG545069.1) | 99.3 | OM791911 | *Ascomycota, Dothideomycetes, Botryosphaeriales* |
| 145 | F6724 | Habitat 3 | Leaves | *Phyllosticta* sp. (MK120855.1) | 98.7 | OM791912 | *Ascomycota, Dothideomycetes, Botryosphaeriales* |
| 146 | F4327 | Habitat 3 | Leaves | *Lasiodiplodia theobromae* (MT644474.1) | 99.3 | OM791932 | *Ascomycota, Dothideomycetes, Botryosphaeriales* |
| 147 | F4331 | Habitat 3 | Leaves | *Colletotrichum gloeosporioides* (MT043801.1) | 100.0 | OM791952 | *Ascomycota, Sordariomycetes, Glomerellales* |
| 148 | F4428 | Habitat 3 | Stems | *Pestalotiopsis* sp. (MT102590.1) | 100.0 | OM791955 | *Ascomycota, Sordariomycetes, Xylariales* |
| 149 | F4430 | Habitat 3 | Leaves | *Colletotrichum scovillei* (LC488852.1) | 100.0 | OM791957 | *Ascomycota, Sordariomycetes, Glomerellales* |
| 150 | F4320 | Habitat 3 | Leaves | *Colletotrichum* sp. (MT570096.1) | 99.3 | OM791972 | *Ascomycota, Sordariomycetes, Glomerellales* |
| 151 | F4324 | Habitat 3 | Leaves | *Nigrospora* sp. (MT645657.1) | 100.0 | OM791973 | *Ascomycota, Sordariomycetes, Xylariales* |
| 152 | F4925 | Habitat 4 | Leaves | *Trichoderma longibrachiatum* (MT316380.1) | 100.0 | OM792008 | *Ascomycota, Sordariomycetes, Hypocreales* |
| 153 | F4926 | Habitat 4 | Frond from fish tail palm | *Arthrinium garethjonesii* (MW481715.1) | 98.5 | OM792009 | *Ascomycota, Sordariomycetes, Xylariales* |
| 154 | F4930 | Habitat 4 | Frond from fish tail palm | *Trichoderma longibrachiatum* (MT316380.1) | 99.0 | OM792010 | *Ascomycota, Sordariomycetes, Hypocreales* |
| 155 | F6550 | Habitat 4 | Leaves | *Colletotrichum asianum* (MT350261.1) | 100.0 | OM792011 | *Ascomycota, Sordariomycetes, Glomerellales* |
| 156 | F6552 | Habitat 4 | Leaves | *Colletotrichum* sp. (MT577046.1) | 99.3 | OM792012 | *Ascomycota, Sordariomycetes, Glomerellales* |
| 157 | F6554 | Habitat 4 | Leaves | *Colletotrichum* sp. (MT577046.1) | 99.3 | OM792013 | *Ascomycota, Sordariomycetes, Glomerellales* |
| 158 | F6559 | Habitat 4 | Stems | *Phomopsis* sp. (MK587755.1) | 100.0 | OM792014 | *Ascomycota, Sordariomycetes, Diaporthales* |
| 159 | F6586 | Habitat 4 | Leaves | *Colletotrichum gloeosporioides* (MT416209.1) | 99.3 | OM792015 | *Ascomycota, Sordariomycetes, Glomerellales* |
| 160 | F6595 | Habitat 4 | Leaves | *Colletotrichum* sp. (MT577046.1) | 100.0 | OM792016 | *Ascomycota, Sordariomycetes, Glomerellales* |
| 161 | F6600 | Habitat 4 | Leaves | *Colletotrichum magnisporum* (MH151055.1) | 97.9 | OM792017 | *Ascomycota, Sordariomycetes, Glomerellales* |
| 162 | F6601 | Habitat 4 | Leaves | *Phyllosticta capitalensis* (MT568601.1) | 99.3 | OM792018 | *Ascomycota, Dothideomycetes, Botryosphaeriales* |
| 163 | F6603 | Habitat 4 | Leaves | *Colletotrichum asianum* (MT350261.1) | 100.0 | OM792019 | *Ascomycota, Sordariomycetes, Glomerellales* |
| 164 | F6608 | Habitat 4 | Stems | *Phomopsis* sp. (MK120615.1) | 100.0 | OM792020 | *Ascomycota, Sordariomycetes, Diaporthales* |
| 165 | F6613 | Habitat 4 | Leaves | *Colletotrichum* sp. (MT577046.1) | 99.3 | OM792021 | *Ascomycota, Sordariomycetes, Glomerellales* |
| 166 | F6614 | Habitat 4 | Leaves | *Colletotrichum siamense* (MT219898.1) | 100.0 | OM792022 | *Ascomycota, Sordariomycetes, Glomerellales* |
| 167 | F6690 | Habitat 4 | Leaves | *Colletotrichum* sp. (MT577046.1) | 99.3 | OM792023 | *Ascomycota, Sordariomycetes, Glomerellales* |
| 168 | F6691 | Habitat 4 | Leaves | *Hypoxylon* sp. (KU683912.1) | 98.3 | OM792024 | *Ascomycota, Sordariomycetes, Xylariales* |
| 169 | F6545 | Habitat 4 | Flowers | *Cosmospora vilior* (JN541223.1) | 100.0 | OM792025 | *Ascomycota, Sordariomycetes, Hypocreales* |
| 170 | F6547 | Habitat 4 | Flowers | *Diaporthe searlei* (NR_168241.1) | 100.0 | OM792026 | *Ascomycota, Sordariomycetes, Diaporthales* |
| 171 | F6553 | Habitat 4 | Leaves | *Phomopsis* sp. (GU066672.1) | 100.0 | OM792027 | *Ascomycota, Sordariomycetes, Diaporthales* |
| 172 | F6555 | Habitat 4 | Stems | *Diaporthe* sp. (KY790594.1) | 97.9 | OM792028 | *Ascomycota, Sordariomycetes, Diaporthales* |
| 173 | F6557 | Habitat 4 | Stems | *Phomopsis* sp. (MK587755.1) | 100.0 | OM792029 | *Ascomycota, Sordariomycetes, Diaporthales* |
| 174 | F6561 | Habitat 4 | Leaves | *Aspergillus flavus* (MT497451.1) | 99.0 | OM792030 | *Ascomycota, Eurotiomycetes, Eurotiales* |
| 175 | F6567 | Habitat 4 | Leaves | *Endomelanconiopsis* sp. (MF579571.1) | 100.0 | OM792031 | *Ascomycota, Dothideomycetes, Botryosphaeriales* |
| 176 | F6569 | Habitat 4 | Leaves | *Neofusicoccum parvum* (MT093349.1) | 99.7 | OM792032 | *Ascomycota, Dothideomycetes, Botryosphaeriales* |
| 177 | F6575 | Habitat 4 | Flowers | *Phomopsis* sp. (KX655604.1) | 100.0 | OM792033 | *Ascomycota, Sordariomycetes, Diaporthales* |
| 178 | F6578 | Habitat 4 | Stems | *Hypoxylon* sp. (KU683912.1) | 98.6 | OM792034 | *Ascomycota, Sordariomycetes, Xylariales* |
| 179 | F6580 | Habitat 4 | Stems | *Diaporthe ambigua* (KU935706.1) | 95.2 | OM792035 | *Ascomycota, Sordariomycetes, Diaporthales* |
| 180 | F6581 | Habitat 4 | Leaves | *Colletotrichum asianum* (MT350261.1) | 100.0 | OM792036 | *Ascomycota, Sordariomycetes, Glomerellales* |
| 181 | F6582 | Habitat 4 | Leaves | *Diaporthe longicolla* (MK119224.1) | 99.7 | OM792037 | *Ascomycota, Sordariomycetes, Diaporthales* |
| 182 | F6584 | Habitat 4 | Leaves | *Colletotrichum* sp. (MT577046.1) | 99.3 | OM792038 | *Ascomycota, Sordariomycetes, Glomerellales* |
| 183 | F6585 | Habitat 4 | Leaves | *Colletotrichum siamense* (MW186174.1) | 100.0 | OM792039 | *Ascomycota, Sordariomycetes, Glomerellales* |
| 184 | F6686 | Habitat 4 | Leaves | *Helminthosporium genistae* (NR_155195.1) | 94.8 | OM792040 | *Ascomycota, Dothideomycetes, Pleosporales* |
| 185 | F6587 | Habitat 4 | Leaves | *Colletotrichum horii* (KR995727.1) | 100.0 | OM792041 | *Ascomycota, Sordariomycetes, Glomerellales* |
| 186 | F6591 | Habitat 4 | Stems | *Colletotrichum asianum* (MT350261.1) | 100.0 | OM792042 | *Ascomycota, Sordariomycetes, Glomerellales* |
| 187 | F6592 | Habitat 4 | Stems | *Phomopsis* sp. (MK311340.1) | 99.7 | OM792043 | *Ascomycota, Sordariomycetes, Diaporthales* |
| 188 | F6548 | Habitat 4 | Flowers | *Cylindrocladiella* sp. (JX243750.1) | 100.0 | OM792044 | *Ascomycota, Sordariomycetes, Hypocreales* |
| 189 | F6549 | Habitat 4 | Flowers | *Cylindrocladiella* sp. (JX243750.1) | 100.0 | OM792045 | *Ascomycota, Sordariomycetes, Hypocreales* |
| 190 | F6597 | Habitat 4 | Leaves | *Colletotrichum* sp. (MT577046.1) | 100.0 | OM792046 | *Ascomycota, Sordariomycetes, Glomerellales* |
| 191 | F6598 | Habitat 4 | Leaves | *Colletotrichum gloeosporioides* (KJ617392.1) | 99.7 | OM792047 | *Ascomycota, Sordariomycetes, Glomerellales* |
| 192 | F6599 | Habitat 4 | Leaves | *Colletotrichum siamense* (MW186174.1) | 100.0 | OM792048 | *Ascomycota, Sordariomycetes, Glomerellales* |
| 193 | F6602 | Habitat 4 | Leaves | *Colletotrichum* sp. (MT577046.1) | 100.0 | OM792049 | *Ascomycota, Sordariomycetes, Glomerellales* |
| 194 | F6606 | Habitat 4 | Stems | *Colletotrichum magnisporum* (MH151055.1) | 99.0 | OM792050 | *Ascomycota, Sordariomycetes, Glomerellales* |
| 195 | F6609 | Habitat 4 | Stems | *Phomopsis phyllanthicola* (MH930424.1) | 99.3 | OM792051 | *Ascomycota, Sordariomycetes, Diaporthales* |
| 196 | F6611 | Habitat 4 | Leaves | *Colletotrichum fructicola* (MT424894.1) | 99.3 | OM792052 | *Ascomycota, Sordariomycetes, Glomerellales* |
| 197 | F6615 | Habitat 4 | Leaves | *Diaporthe* sp. (MT568516.1) | 100.0 | OM792053 | *Ascomycota, Sordariomycetes, Diaporthales* |
| 198 | F6617 | Habitat 4 | Leaves | *Simplicillium* sp. (AB378536.1) | 100.0 | OM792054 | *Ascomycota, Sordariomycetes, Hypocreales* |
| 199 | F6688 | Habitat 4 | Leaves | *Colletotrichum asianum* (MT350261.1) | 100.0 | OM792055 | *Ascomycota, Sordariomycetes, Glomerellales* |
| 200 | F6693 | Habitat 4 | Leaves | *Cosmospora vilior* (JN541223.1) | 100.0 | OM792056 | *Ascomycota, Sordariomycetes, Hypocreales* |
| 201 | F10400 | Habitat 4 | Leaves | *Sarocladium* sp. (MN128515.1) | 98.3 | OM792057 | *Ascomycota, Sordariomycetes, Hypocreales* |
| 202 | F10390 | Habitat 4 | Leaves | *Coprinopsis cinerea* (MH443753.1) | 100.0 | OM792058 | *Basidiomycota, Agaricomycetes, Agaricales* |
| 203 | F6610 | Habitat 4 | Stems | *Phomopsis asparagi* (MT459267.1) | 97.6 | OM792059 | *Ascomycota, Sordariomycetes, Diaporthales* |
| 204 | F6616 | Habitat 4 | Leaves | *Cosmospora vilior* (JN541223.1) | 100.0 | OM792060 | *Ascomycota, Sordariomycetes, Hypocreales* |
| 205 | F11602 | Habitat 4 | Basidiome | *Neonectria* sp. (KY413710.1) | 100.0 | OM792061 | *Ascomycota, Sordariomycetes, Hypocreales* |
| 206 | F6620 | Habitat 4 | Leaves | *Colletotrichum* sp. (MT577046.1) | 100.0 | OM792062 | *Ascomycota, Sordariomycetes, Glomerellales* |
| 207 | F11652 | Habitat 4 | Fern | *Pseudocercospora humuli* (GU214676.1) | 98.9 | OM792063 | *Ascomycota, Dothideomycetes, Mycosphaerellales* |
| 208 | F11610 | Habitat 4 | Fern/Frond | *Colletotrichum asianum* (MT350261.1) | 100.0 | OM792064 | *Ascomycota, Sordariomycetes, Glomerellales* |
| 209 | F11616 | Habitat 4 | Leaves | *Diaporthe* sp. (MK229151.1) | 98.0 | OM792065 | *Ascomycota, Sordariomycetes, Diaporthales* |
| 210 | F11608 | Habitat 4 | Fern | *Colletotrichum gloeosporioides* (MT557555.1) | 99.3 | OM792066 | *Ascomycota, Sordariomycetes, Glomerellales* |
| 211 | F6612 | Habitat 4 | Leaves | *Colletotrichum fructicola* (MT424894.1) | 100.0 | OM792067 | *Ascomycota, Sordariomycetes, Glomerellales* |
| 212 | F4923 | Habitat 4 | Leaves | *Daldinia eschscholtzii* (MT065673.1) | 100.0 | OM792068 | *Ascomycota, Sordariomycetes, Xylariales* |
| 213 | F6692 | Habitat 4 | Leaves | *Colletotrichum* sp. (MT577046.1) | 100.0 | OM792069 | *Ascomycota, Sordariomycetes, Glomerellales* |
| 214 | F6697 | Habitat 4 | Leaves | *Daldinia eschscholtzii* (KY440188.1) | 100.0 | OM792070 | *Ascomycota, Sordariomycetes, Xylariales* |
| 215 | F11601 | Habitat 4 | Basidiome | *Fomitopsis feei* (MG437308.1) | 100.0 | OM792071 | *Basidiomycota, Agaricomycetes, Polyporales* |
| 216 | F11607 | Habitat 4 | Fern/frond | *Hypoxylon polyporoideum* (JQ009311.1) | 99.7 | OM792072 | *Ascomycota, Sordariomycetes, Xylariales* |
| 217 | F11609 | Habitat 4 | Fern | *Malaysiasca phaii* (NR_154193.1) | 95.0 | OM792073 | *Ascomycota, Sordariomycetes, Glomerellales* |
| 218 | F11615 | Habitat 4 | Leaves | *Hansfordia* sp. (MW133921.1) | 98.9 | OM792074 | *Ascomycota, Sordariomycetes, Xylariales* |
| 219 | F11618 | Habitat 4 | Leaves | *Fumiglobus pieridicola* (NR_153985.1) | 93.3 | OM792075 | *Ascomycota, Dothideomycetes, Capnodiales* |
| 220 | F11651 | Habitat 4 | Fern | *Muyocopron chromolaenicola* (NR_168858.1) | 95.3 | OM792076 | *Ascomycota, Dothideomycetes, Muyocopronales* |
| 221 | F11653 | Habitat 4 | Fern | *Paramicrothyrium* sp. (KU747847.1) | 100.0 | OM792077 | *Ascomycota, Dothideomycetes, incertae sedis* |
| 222 | F11663 | Habitat 4 | Leaves | *Scolecoxyphium blechni* (NR_168838.1) | 96.8 | OM792078 | *Ascomycota, Dothideomycetes, Capnodiales* |

*Natural Product Library ID

**Table S2**. Categorization of active fungal isolates subjected to chemical dereplication according to target pathogens SA (*Staphylococcus aureus*), KA (*Klebsiella aerogenes*), CA (*Candida albicans*), AF (*Aspergillus fumigatus*) and cancer cell lines A5 (A549), MP (MIA PaCa-2), PC (PANC-1). Compounds tentatively identified by Dictionary of Natural Products database, authentic standard, and/or MS/MS literature data comparison.

| Strain ID | Taxonomy | Primary Screening | | | | | | | |  | Antimicrobial activity | | Cytotoxic activity | | | | |  |  |  |  |  |
| --- | --- | --- | --- | --- | --- | --- | --- | --- | --- | --- | --- | --- | --- | --- | --- | --- | --- | --- | --- | --- | --- | --- |
|  |  | Antimicrobial activity | | | |  | Cytotoxic activity | | |  |  |  |  |  |  |  |  |  |  |  |  |  |
|  |  | SA | KA | CA | AF |  | A5 | MP | PC |  | LC-MS Dereplication (Putative compounds) | | LC-MS Dereplication (Putative compounds) | | | | |  |  |  |  |  |
| F2682 | *Aspergillus flavus* |  |  |  |  |  |  |  |  |  |  | | No activity was observed in any fraction | | | | |  |  |  |  |  |
| F4434 | *Aspergillus assiutensis* |  |  |  |  |  |  |  |  |  | Asnovolin G, Secalonic acids A* and G | | Alterporriols F and G | | | | |  |  |  |  |  |
| F4437 | *Coniella heterospora* |  |  |  |  |  |  |  |  |  |  | | Chaetomugilin T, , Cylindrocladin B, Macrophorin A, Laccaridione B | | | | |  |  |  |  |  |
| F4448 | *Aspergillus assiutensis* |  |  |  |  |  |  |  |  |  | Aspergiterpenoid A, neoxaline and secalonic acid A* and G | | Alterporriols F and G | | | | |  |  |  |  |  |
| F4930 | *Trichoderma longibrachiatum* |  |  |  |  |  |  |  |  |  | Paracelsin H, I and D, Trichocellin AII and AIV and Trilongin BIII* | | Paracelsin H, I and D, Trichocellin AII and AIV and Trilongin BIII* | | | | |  |  |  |  |  |
| F5906 | *Colletotrichum asianum* |  |  |  |  |  |  |  |  |  |  | | Cytochalasin C and D* | | | | |  |  |  |  |  |
| F5910 | *Diaporthe* sp. |  |  |  |  |  |  |  |  |  |  | | PM 181110 | | | | |  |  |  |  |  |
| F5912 | *Muyocopron laterale* |  |  |  |  |  |  |  |  |  | No known molecule found | |  | | | | |  |  |  |  |  |
| F5914 | *Diaporthe phaseolorum* |  |  |  |  |  |  |  |  |  |  | | Phomopsichalasin D and Cytochalasin W | | | | |  |  |  |  |  |
| F5931 | *Fusarium proliferatum* |  |  |  |  |  |  |  |  |  | Glisoprenin E | |  | | | | |  |  |  |  |  |
| F6361 | *Metarhizium anisopliae* |  |  |  |  |  |  |  |  |  | Amauromine, conocandin, and coriolide | |  | | | | |  |  |  |  |  |
| F6578 | *Hypoxylon* sp. |  |  |  |  |  |  |  |  |  | No activity was observed in any fraction | |  | | | | |  |  |  |  |  |
| F6580 | *Diaporthe ambigua* |  |  |  |  |  |  |  |  |  | No activity was observed in any fraction | |  | | | | |  |  |  |  |  |
| F6609 | *Phomopsis phyllanthicola* |  |  |  |  |  |  |  |  |  |  | | Phomopsichalasin G and Cytochalasin O and H* | | | | |  |  |  |  |  |
| F6610 | *Phomopsis asparagi* |  |  |  |  |  |  |  |  |  | Epicoccamide A*, Epicoccamide D* | |  | | | | |  |  |  |  |  |
| F6615 | *Diaporthe phaseolorum* |  |  |  |  |  |  |  |  |  |  | | Phomopsichalasin G and Cytochalasin O and H* | | | | |  |  |  |  |  |
| F6616 | *Cosmospora vilior* |  |  |  |  |  |  |  |  |  |  | | No activity was observed in any fraction | | | | |  |  |  |  |  |
| F6916 | *Acremonium hennebertii* |  |  |  |  |  |  |  |  |  | Ergosta-6,22-dien-3-ol, 5,8-epidioxy-, 3-formate, (3β,5α,8α,22E) | |  | | | | |  |  |  |  |  |
| F6932 | *Lophiotrema* sp. |  |  |  |  |  |  |  |  |  | No known molecule found | | Palmarumycin C15, Palmarumycin C2* and Guignardin E | | | | |  |  |  |  |  |
| F10129 | *Phomopsis asparagi* |  |  |  |  |  |  |  |  |  | 6-epicerevisterol and antibiotic M 6124 | |  | | | | |  |  |  |  |  |
| F10134 | *Fusarium petroliphilum* |  |  |  |  |  |  |  |  |  | No activity was observed in any fraction | |  | | | | |  |  |  |  |  |
| F10136 | *Fusarium proliferatum* |  |  |  |  |  |  |  |  |  | Fumonisin B1*, Fumonisin A1* | |  | | | | |  |  |  |  |  |
| F10145 | *Phaeoacremonium rubrigenum* |  |  |  |  |  |  |  |  |  | No activity was observed in any fraction | |  | | | | |  |  |  |  |  |
| F10154 | *Colletotrichum tropicicola* |  |  |  |  |  |  |  |  |  | No known molecule found | |  | | | | |  |  |  |  |  |
| F10163 | *Phialemoniopsis curvata* |  |  |  |  |  |  |  |  |  | Gabusectin | |  | | | | |  |  |  |  |  |
| F10164 | *Muyocopron laterale* |  |  |  |  |  |  |  |  |  | Lactariolide | |  | | | | |  |  |  |  |  |
| F10318 | *Bartalinia* sp. |  |  |  |  |  |  |  |  |  | Secalonic acid A* and G | | Secalonic acid A* | | | | |  |  |  |  |  |
| F10400 | *Sarocladium* sp. |  |  |  |  |  |  |  |  |  |  | | Vaccinol G, H and O, apiosporamide | | | | |  |  |  |  |  |
| **Total** | | **14** | **1** | **7** | **8** |  | **15** | **22** | **14** |  |  |  | |  |  |  |  | |  |  |  |  |
|  |  | Key |  |  |  |  |  |  |  |  |  |  | |  |  |  |  | |  |  |  |  |
|  |  |  | Active | |  |  |  |  |  |  |  |  | |  |  |  |  | |  |  |  |  |
|  |  |  | Inactive | |  |  |  |  |  |  |  |  | |  |  |  |  | |  |  |  |  |

*Validated by LCMS authentic standard and MS/MS literature data

Active = Average % growth inhibition ≥ 50

Inactive = Average % growth inhibition <50

**
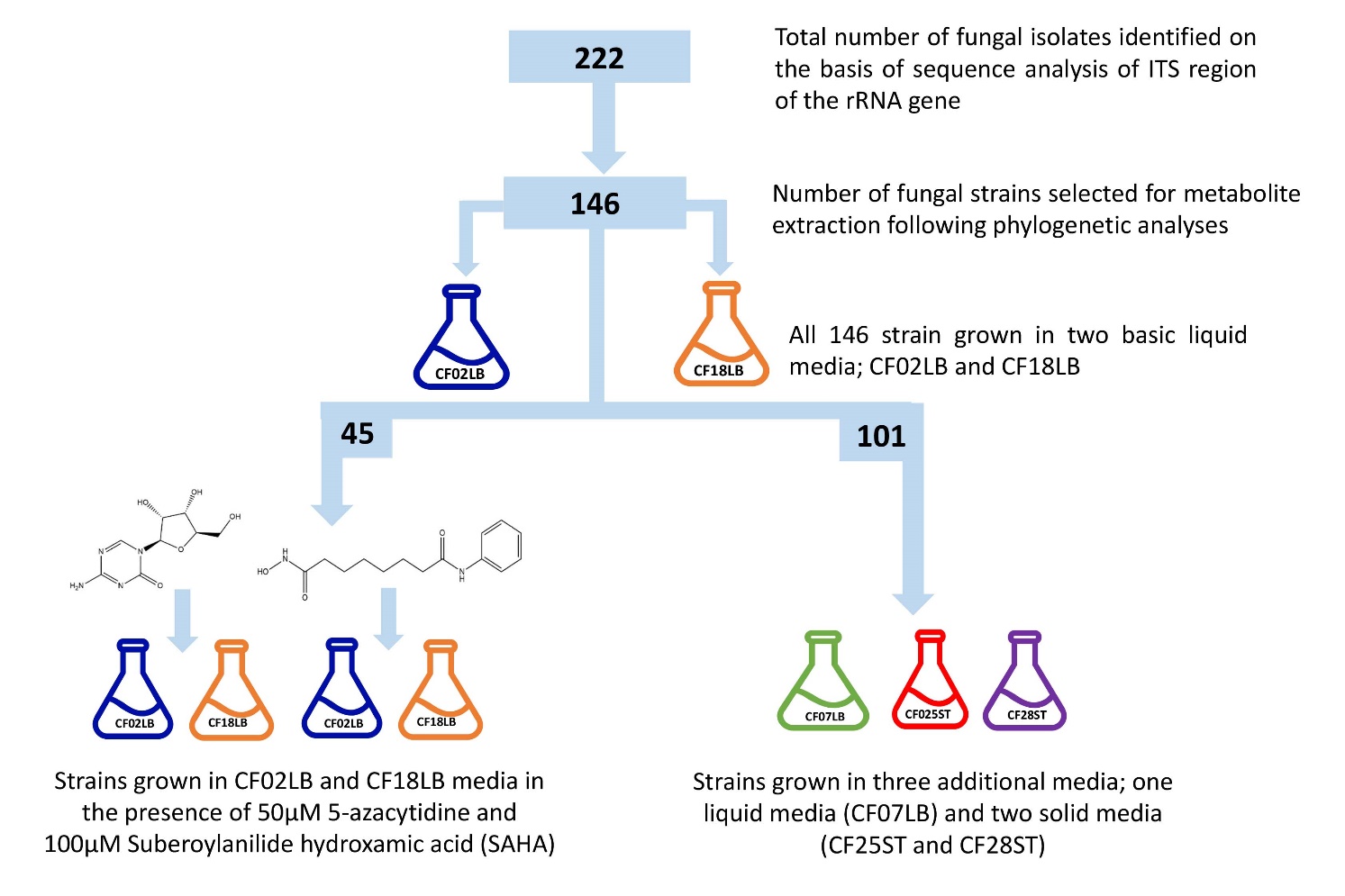
**

**Figure S1**. Experimental design for extracts generation to evaluate the effects of chemical epigenetic modifiers and variation of growth media on the biological activity of fungal extracts.

**
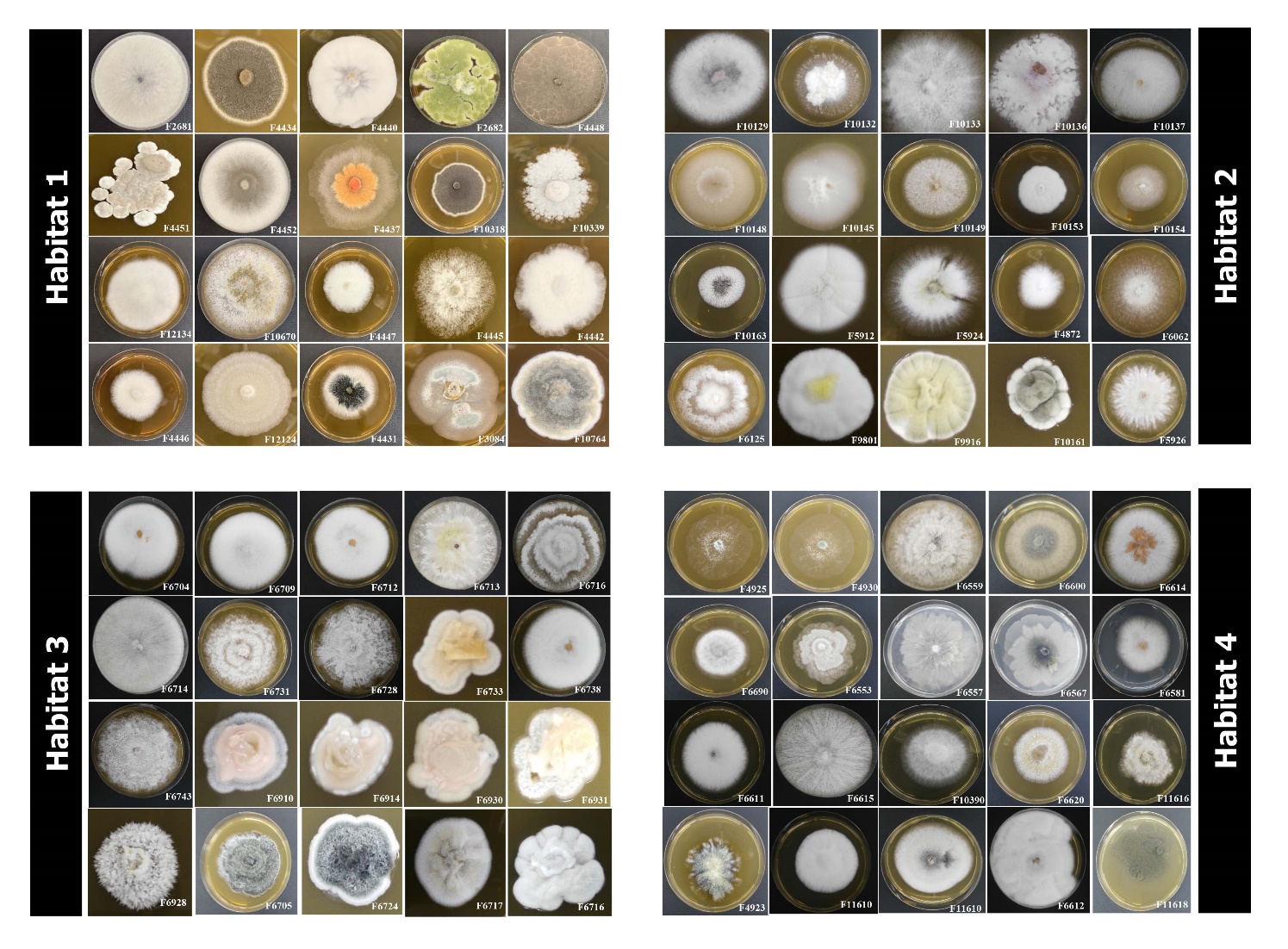
**

**Figure S2.** Morphological diversity of fungal endophytes. Shown here are representative plate pictures of fungal isolates from each of the four habitats in Singapore. Habitat 1 (Upper Seletar Reservoir Park, Upper Pierce Reservoir and MacRitchie Reservoir), Habitat 2 (Cluny road along Singapore botanic garden), Habitat 3 (Pulau Ubin and St. John’s islands) and Habitat 4 (Bukit Timah Nature Reserve and Kent Ridge Park.

**
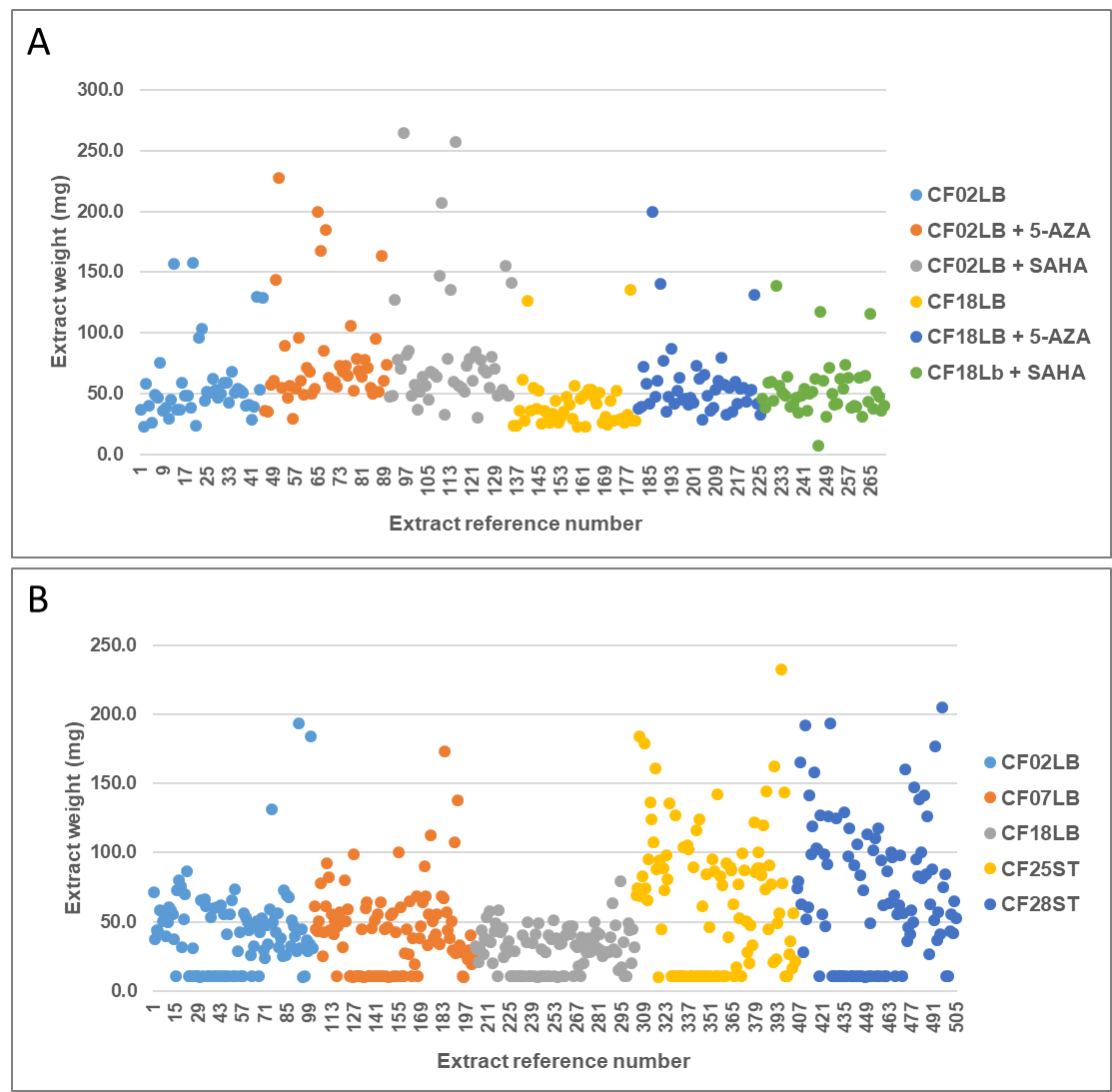
**

**Figure S3.** A comparison of mass of crude extracts from: (A) Fungal strains grown in the two liquid media in the presence and absence of the two chemical elicitors and (B) strains grown in three liquid media (CF02LB, CF18LB and CF07LB) and two solid media (CF25ST and CF28ST).


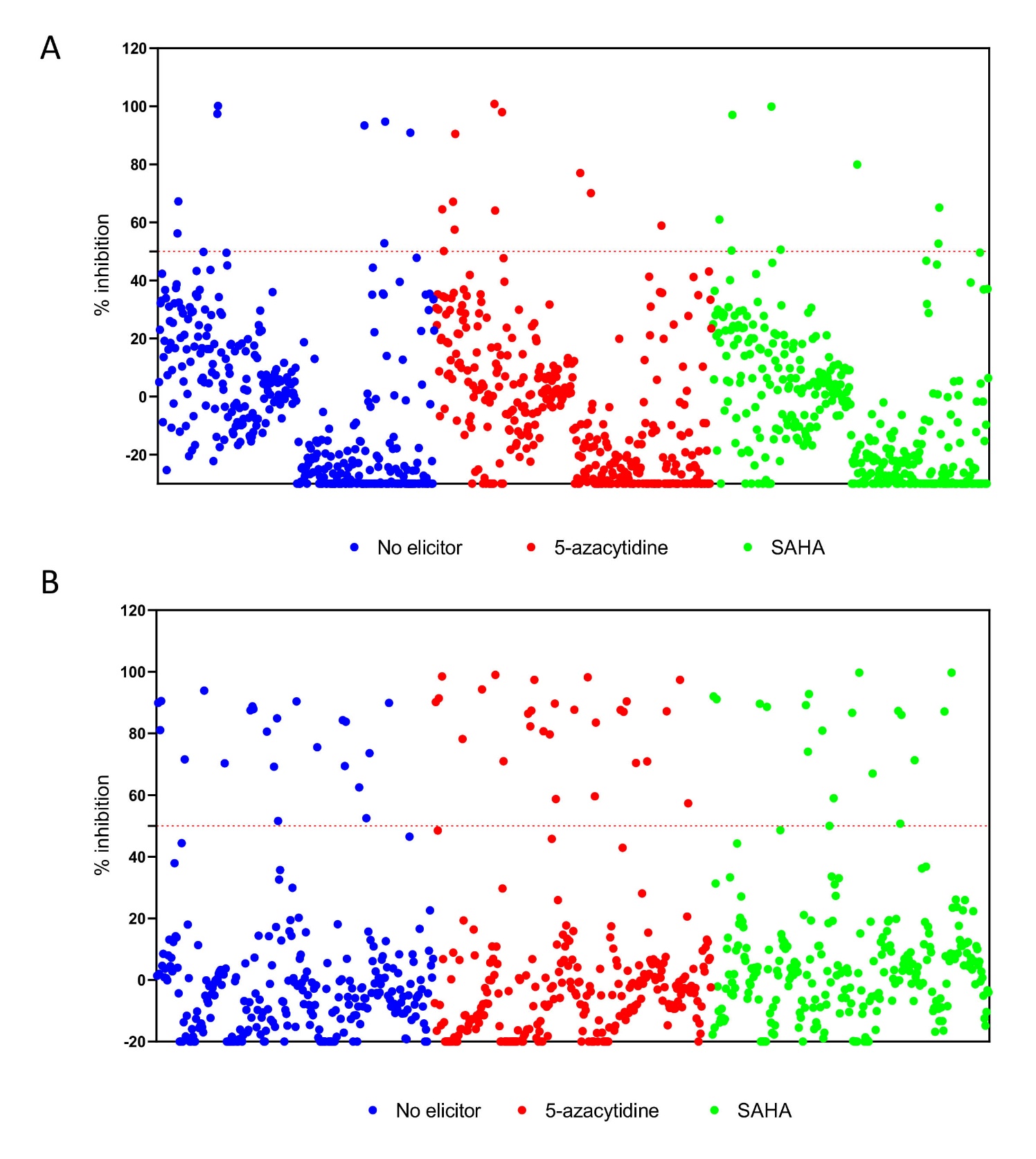


**Figure S4.** Distribution of antimicrobial hits (A) and cytotoxic hits (B) from 45 fungal strains grown in the presence of two chemical elicitors; 5-azacytidine and suberoylanilide hydroxamic acid (SAHA) and in the absence of chemical elicitation. Hits above the red dotted line represent average % inhibition ≥ 50.

**
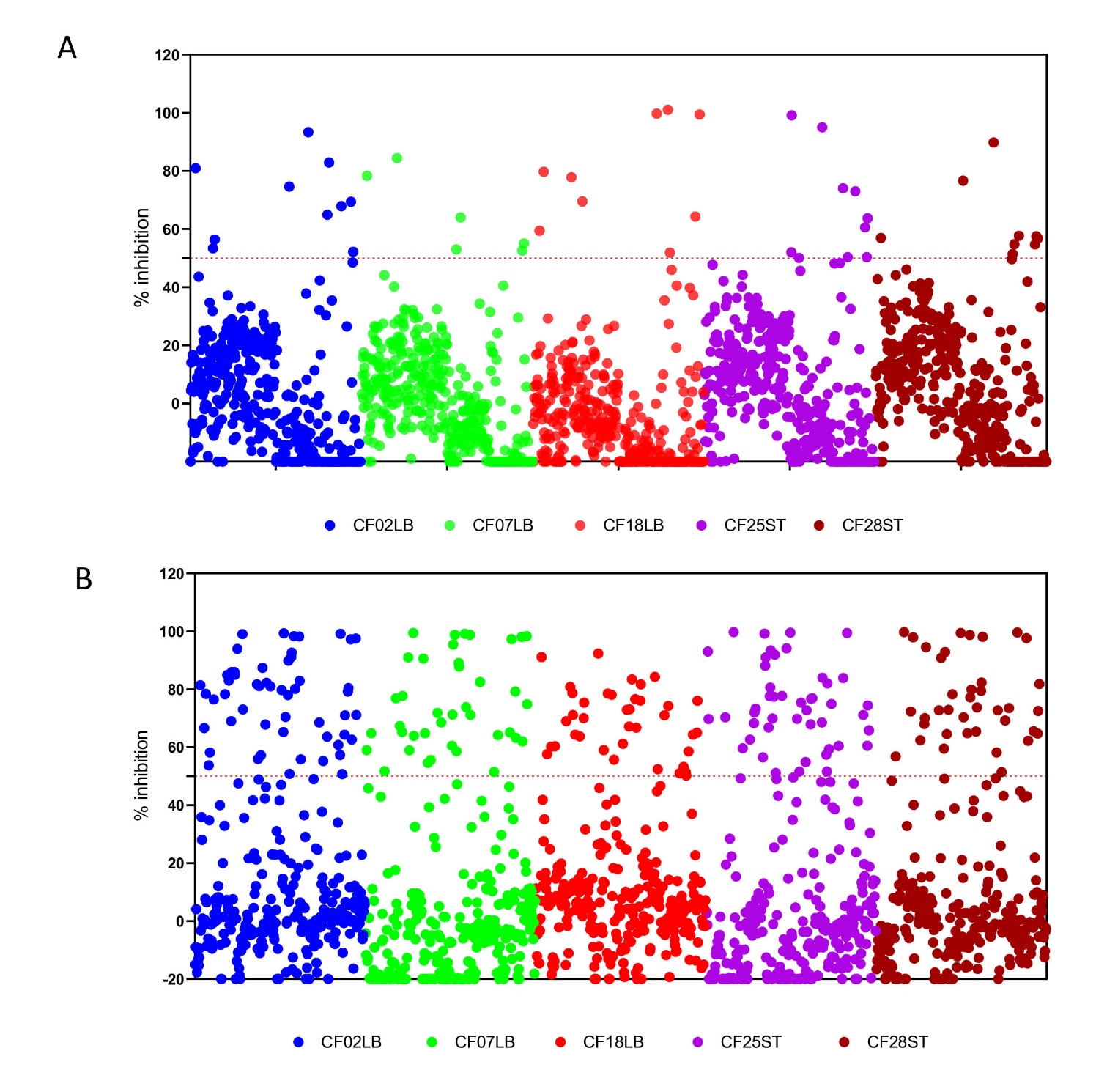
**

**Figure S5.** Distribution of antimicrobial hits (A) and cytotoxic hits (B) from 101 fungal strains grown in five different media; three liquid (CF02LB, CF18LB and CF07LB) and two solid media (CF05ST, CF25ST). Hits above the red dotted line represent average % inhibition ≥ 50.


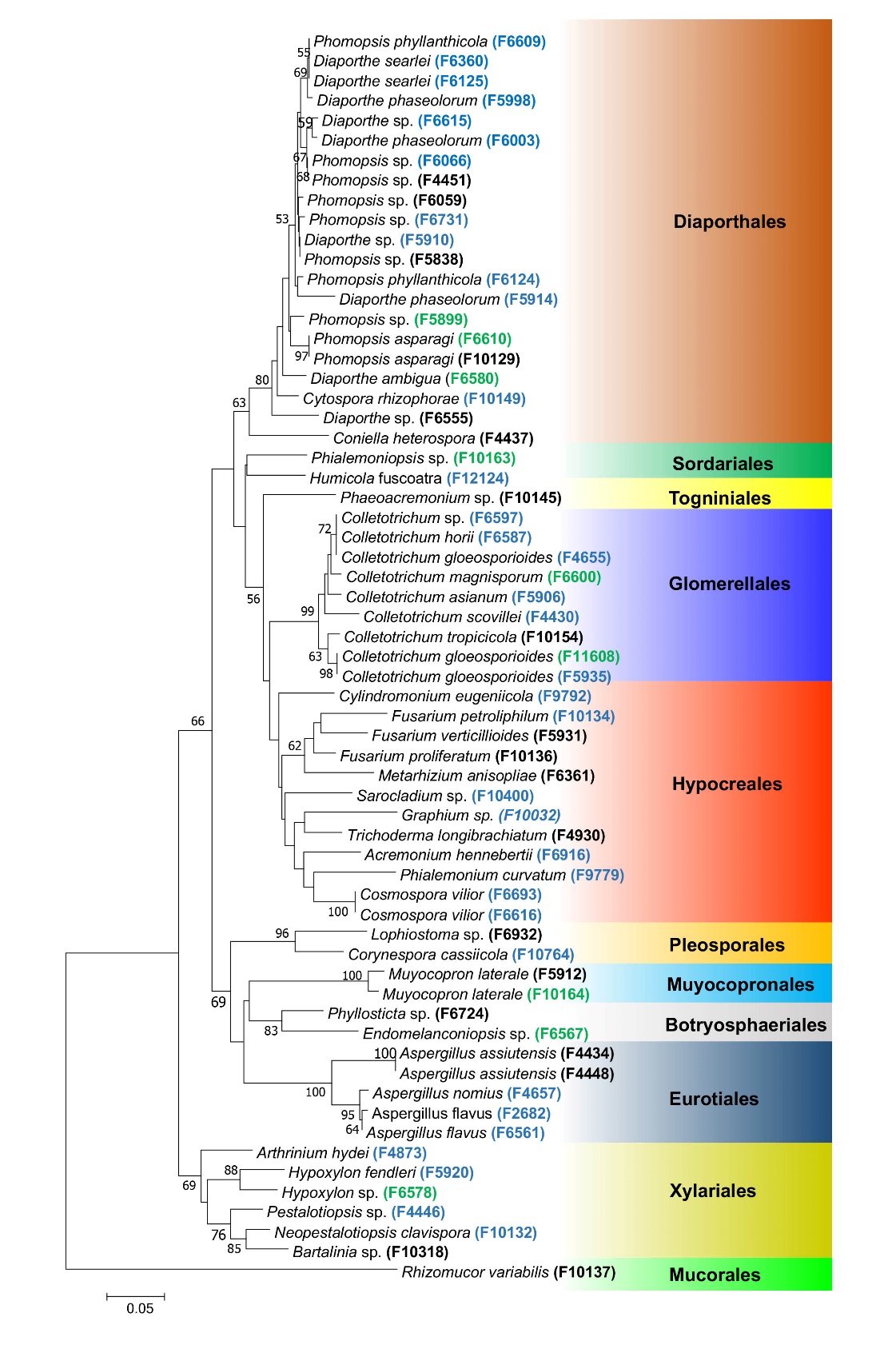


**Figure S6.** Phylogenetic tree showing 63 active fungal strains. The tree is based on internal transcribed spacer (ITS2) rDNA gene sequences and was constructed using the neighbor-joining method with MEGA 7 software (Bootstrap value = 500; values below 50% are not shown). Strains whose ID codes are shown in green color exhibited antimicrobial activity, those shown in blue revealed cytotoxic activity while those shown in black revealed both antimicrobial and cytotoxic activity.


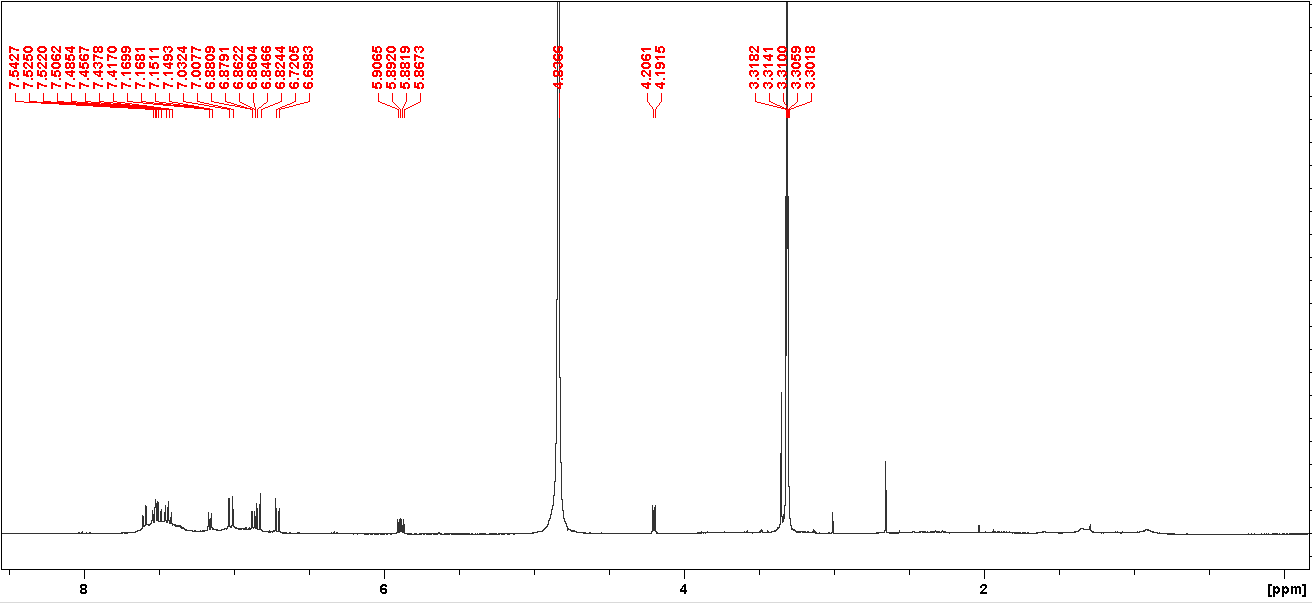


**Figure S7.** ^1^H NMR spectrum (CD_3_OD, 400 MHz) of palmarumycin CP_30_ (**1**).


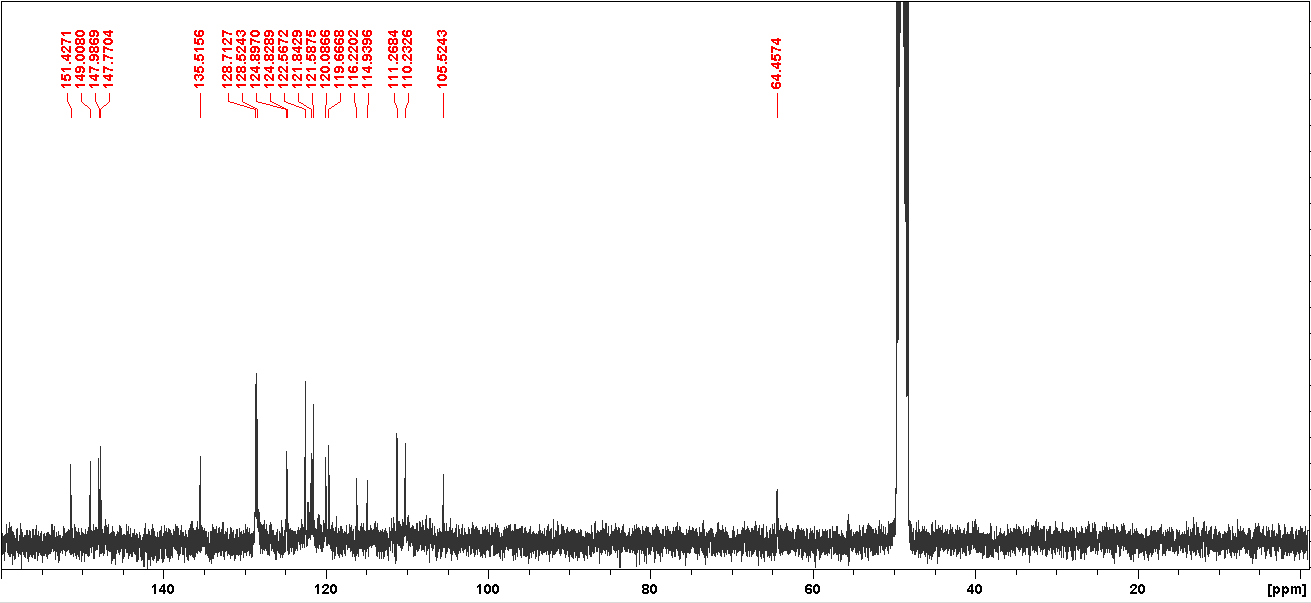


**Figure S8.** ^13^C NMR spectrum (CD_3_OD, 100 MHz) of palmarumycin CP_30_ (**1**).


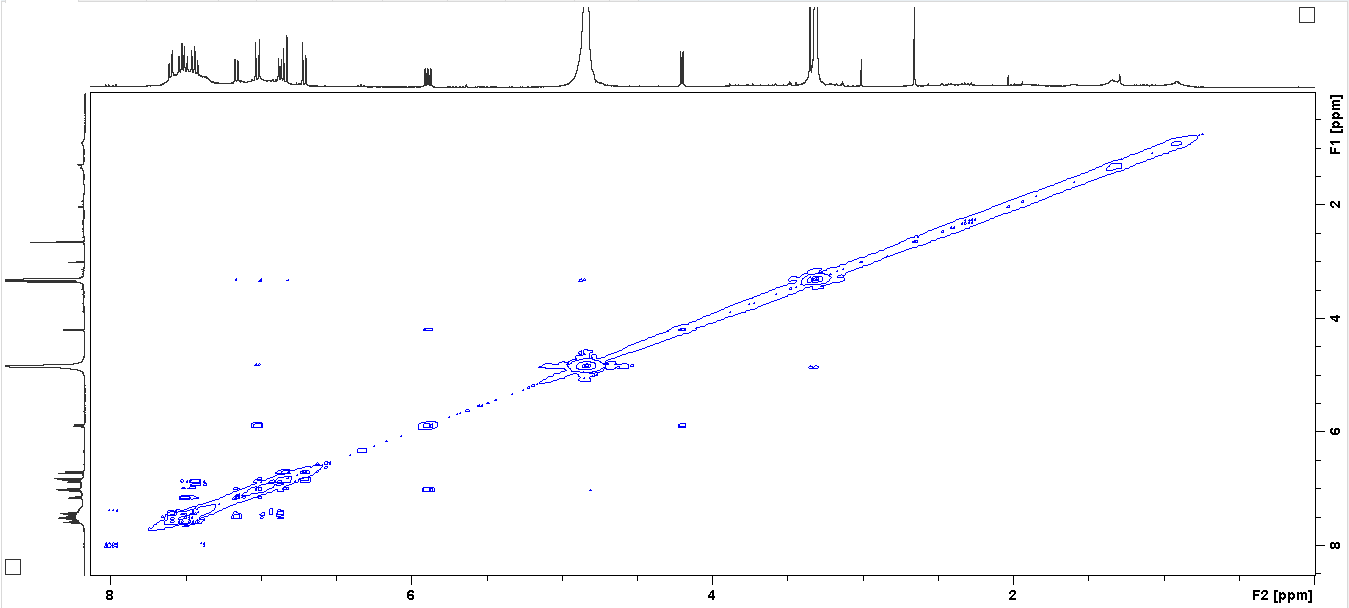


**Figure S9.** COSY spectrum (CD_3_OD) of palmarumycin CP_30_ (**1**).


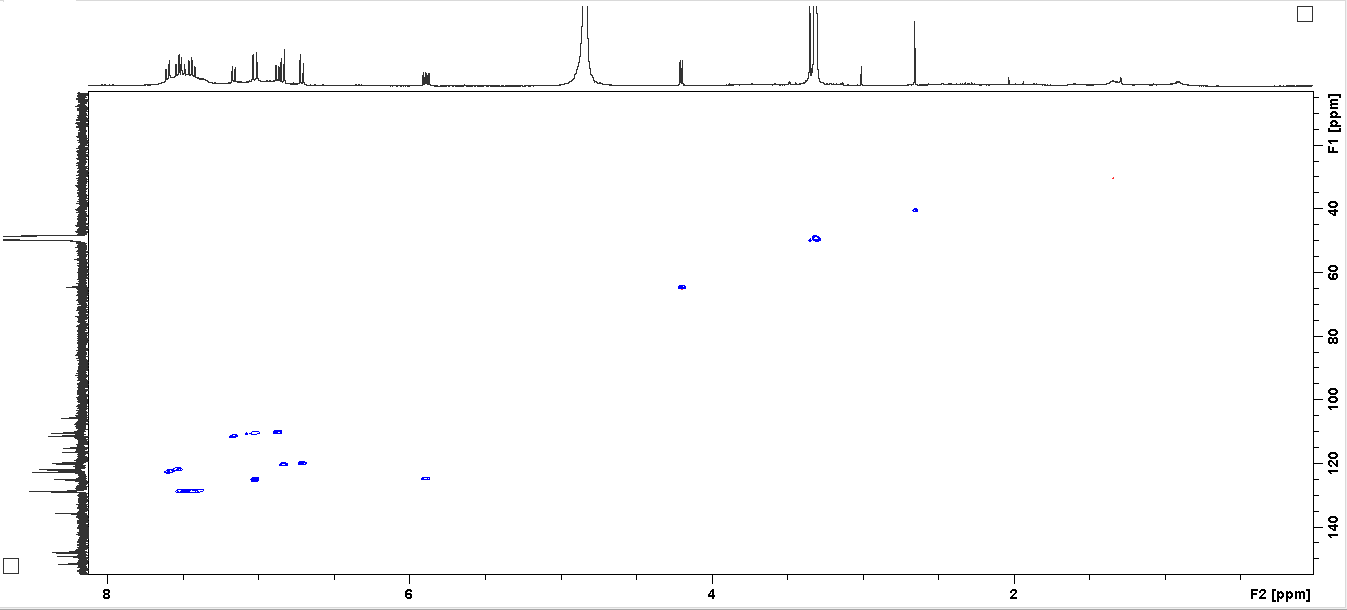


**Figure S10.** HSQC spectrum (CD_3_OD) of palmarumycin CP_30_ (**1**).


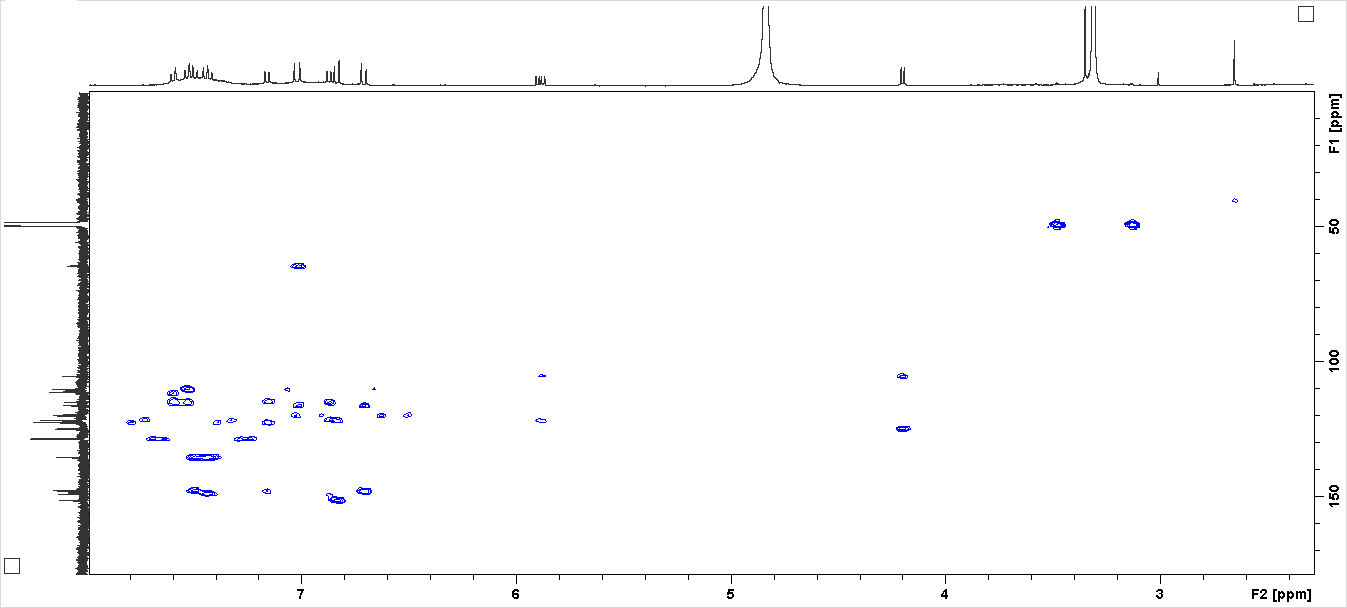


**Figure S11.** HMBC spectrum (CD_3_OD) of palmarumycin CP_30_ (**1**).


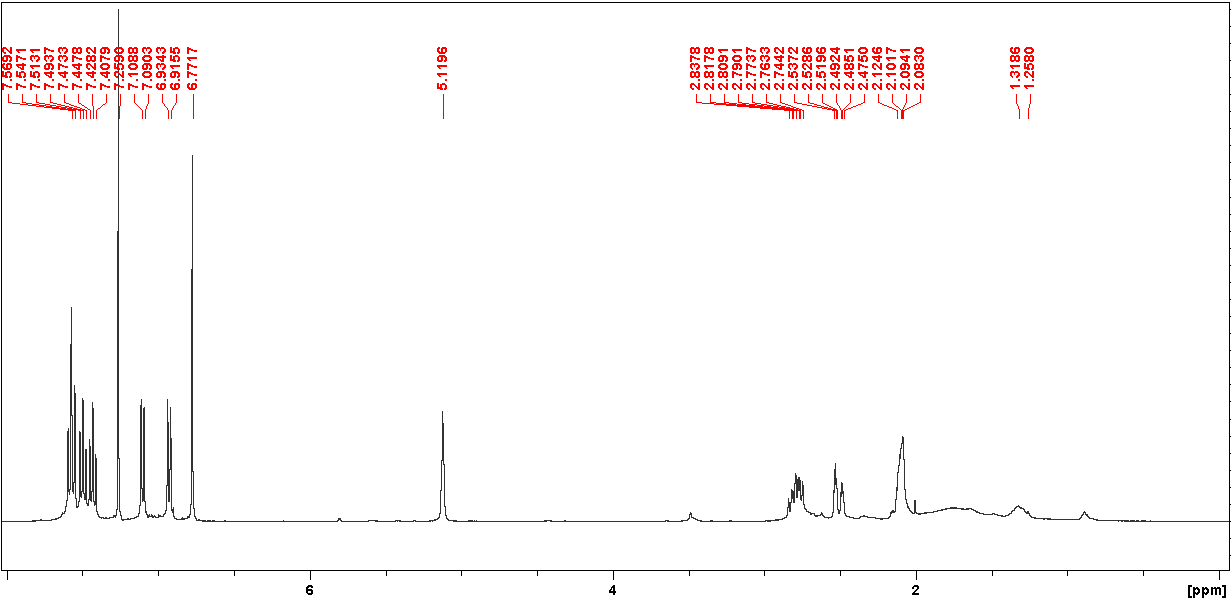


**Figure S12.** ^1^H NMR spectrum (CD_3_OD, 400 MHz) of palmarumycin C_8_ (**2**).


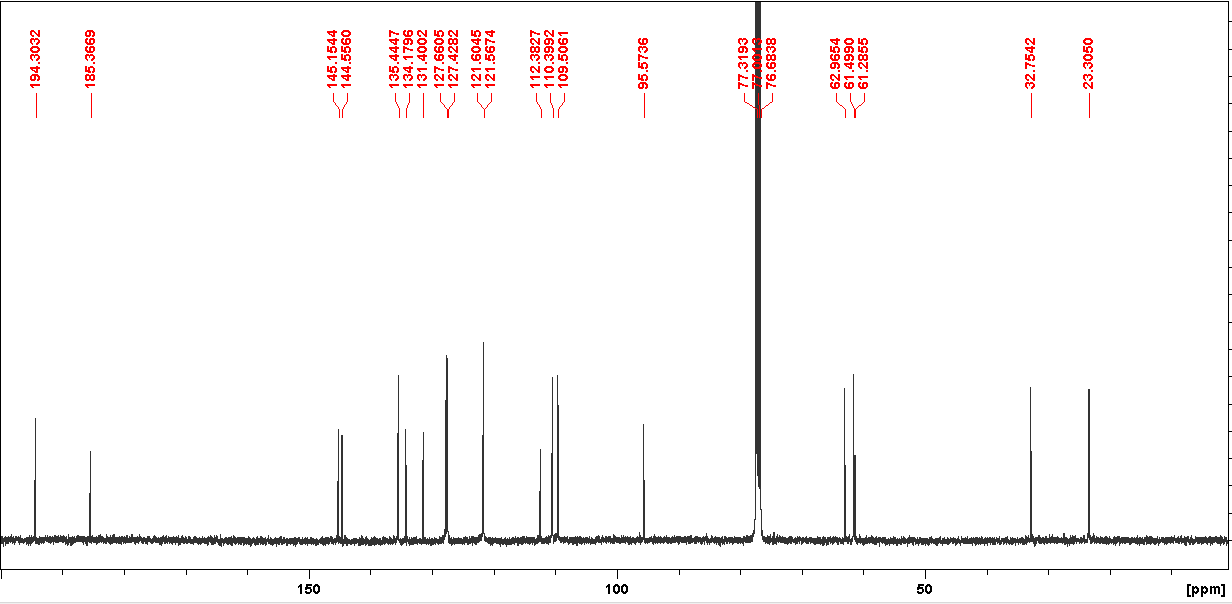


**Figure S13.** ^13^C NMR spectrum (CD_3_OD, 100 MHz) of palmarumycin C_8_ (**2**).


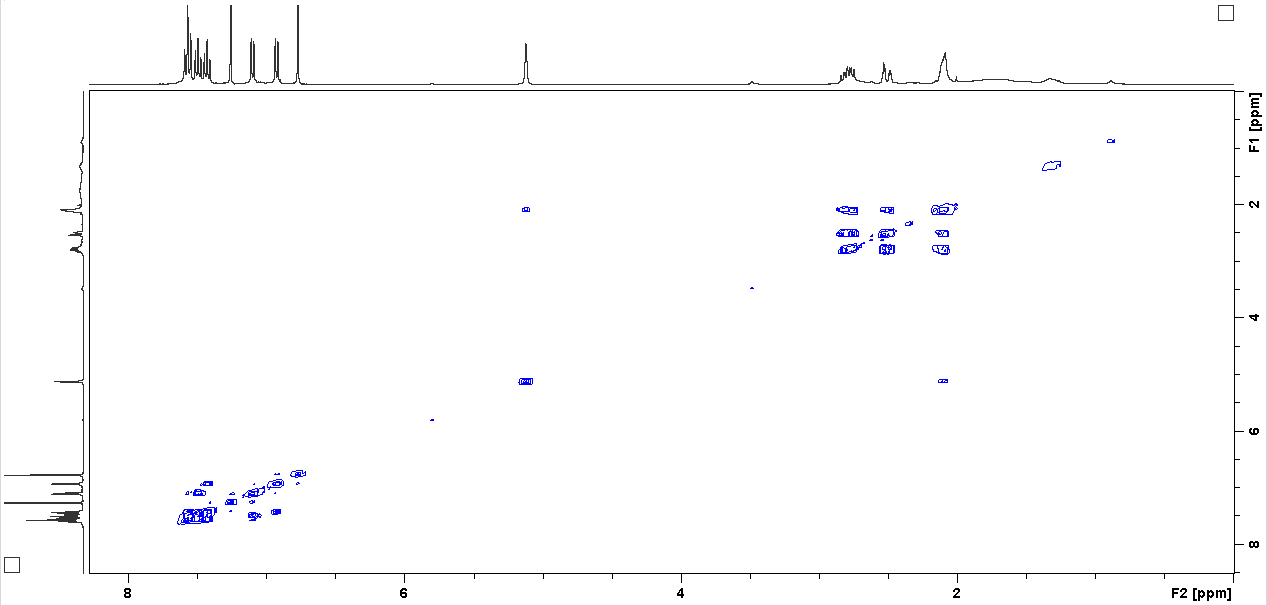


**Figure S14.** COSY spectrum (CD_3_OD) of palmarumycin C_8_ (**2**).


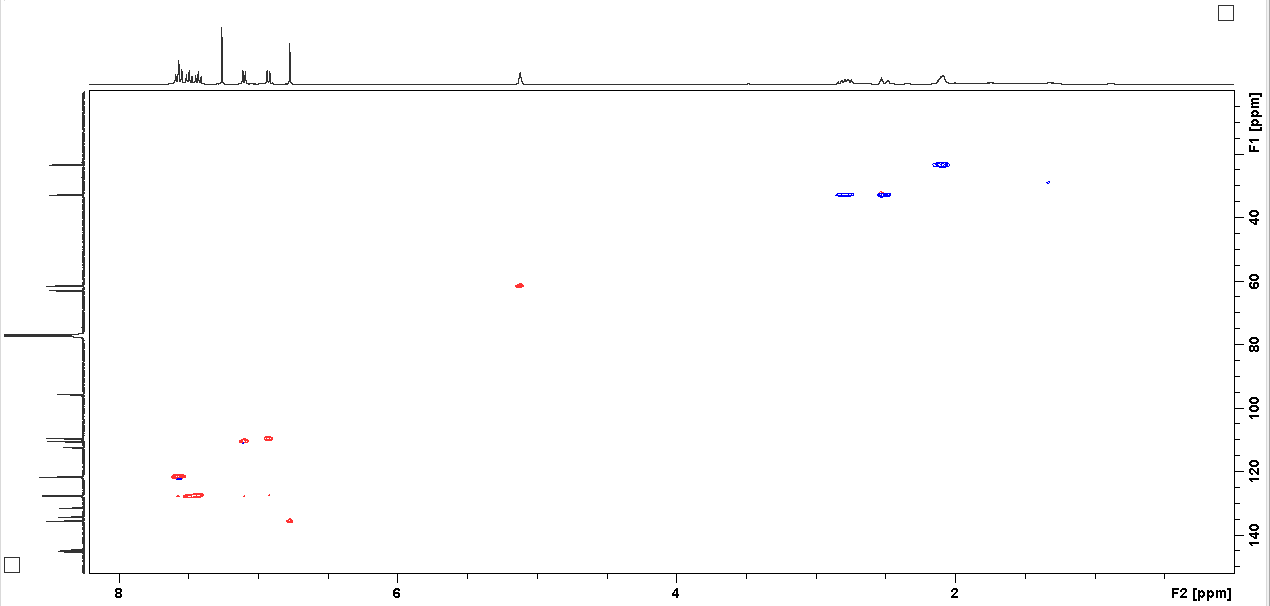


**Figure S15.** HSQC spectrum (CD_3_OD) of palmarumycin C_8_ (**2**).


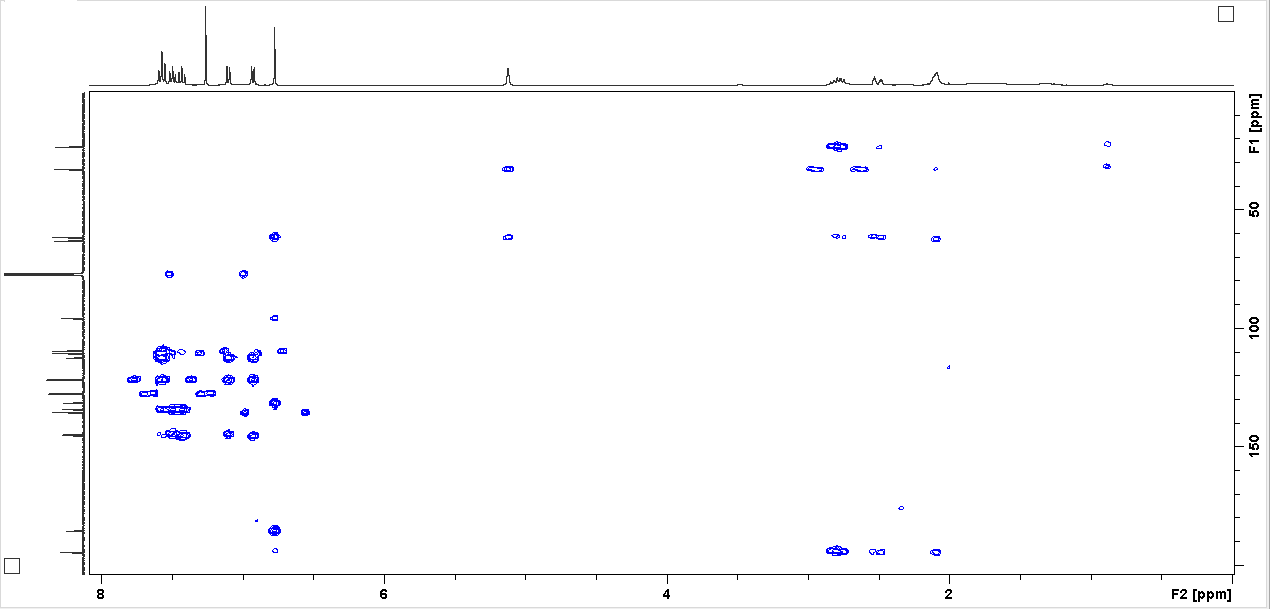


**Figure S16.** HMBC spectrum (CD_3_OD) of palmarumycin C_8_ (**2**).


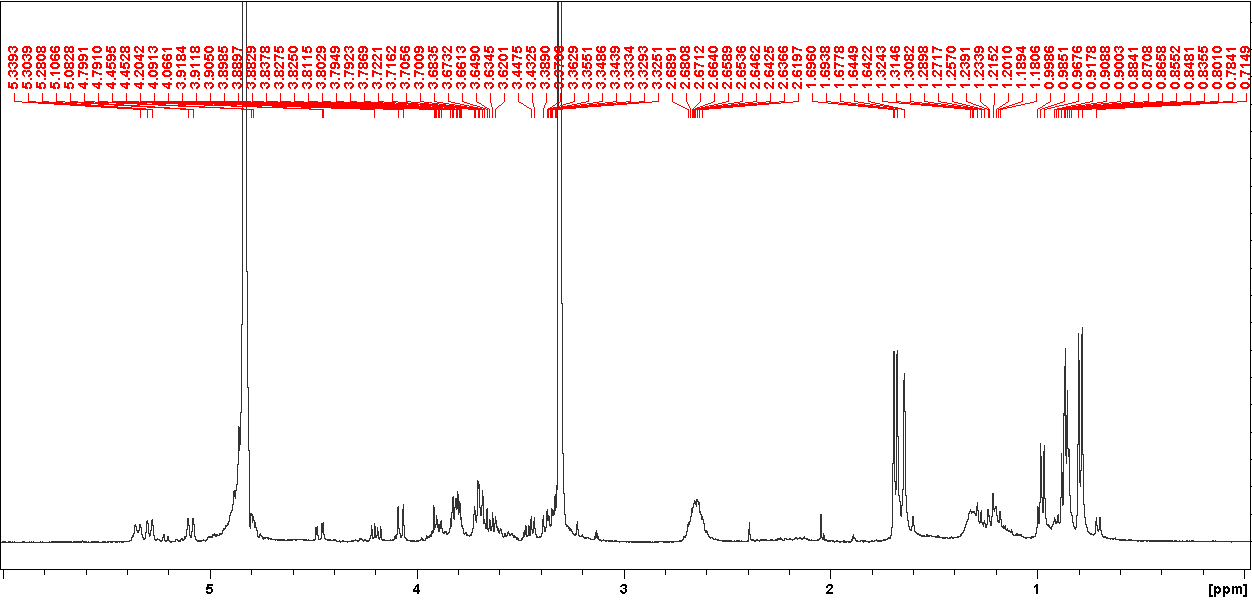


**Figure S17.** ^1^H NMR spectrum (CD_3_OD, 400 MHz) of muyocopronol A (**3**).


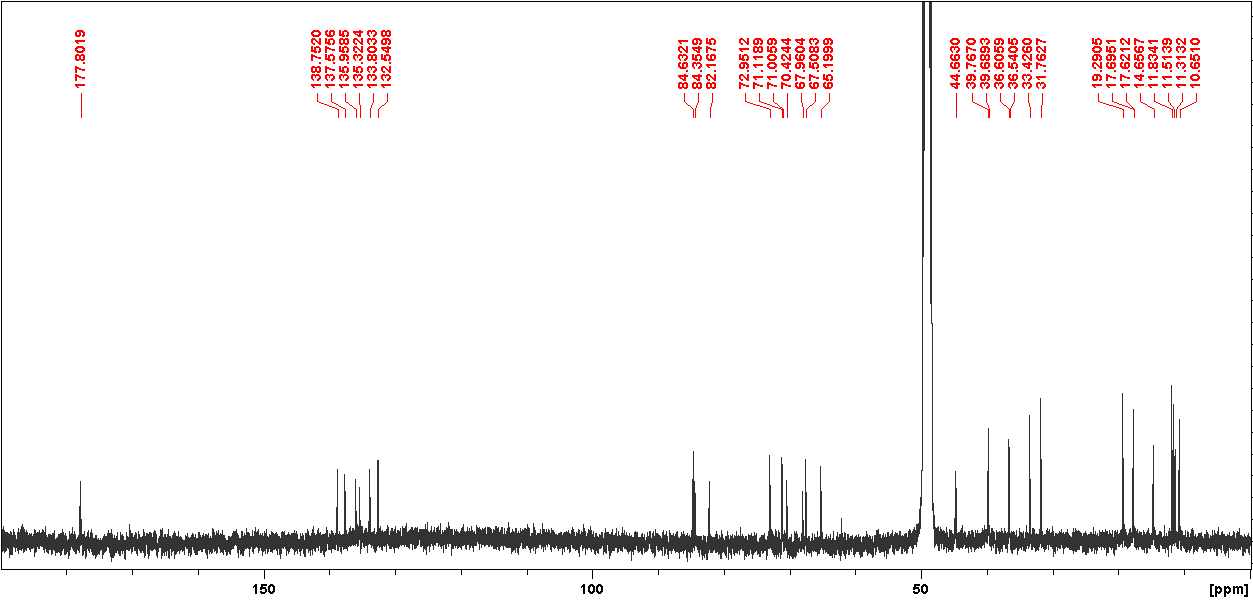


**Figure S18.** ^13^C NMR spectrum (CD_3_OD 100 MHz) of muyocopronol A (**3**).


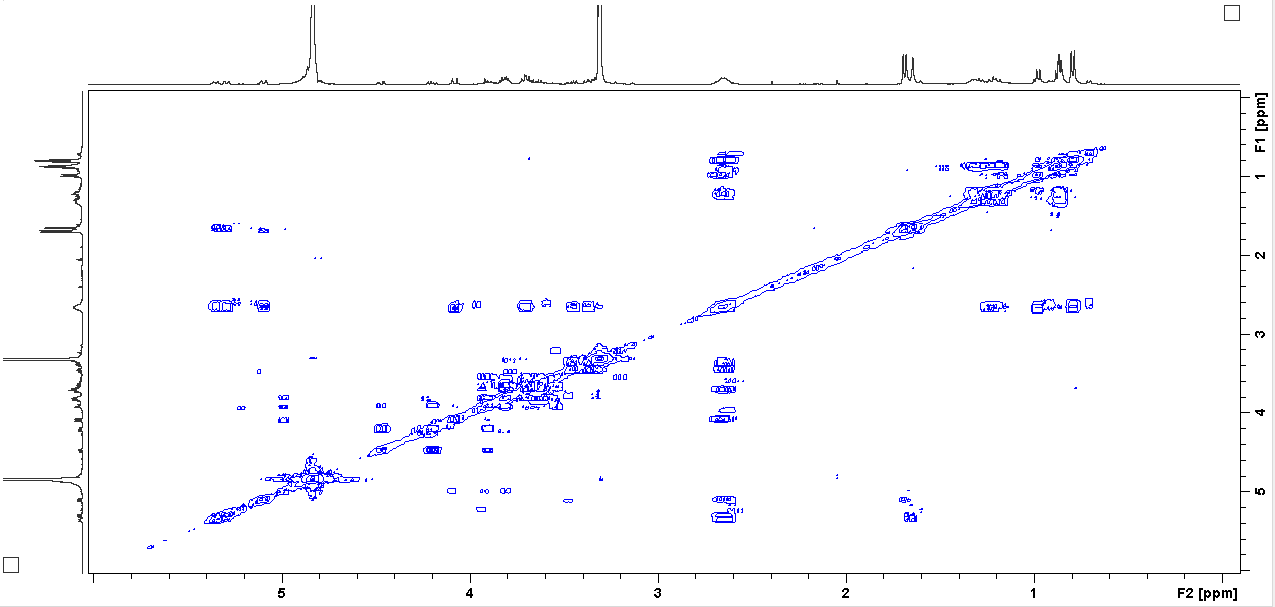


**Figure S19.** COSY spectrum (CD_3_OD) of muyocopronol A (**3**).


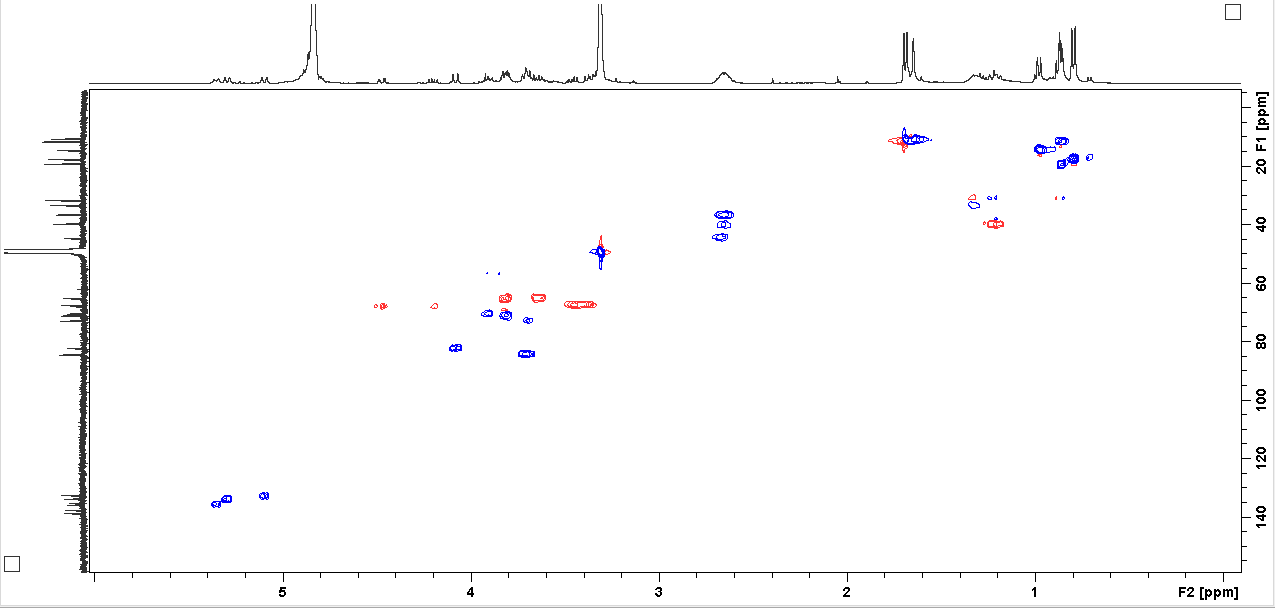


**Figure S20.** HSQC spectrum (CD_3_OD) of muyocopronol A (**3**).


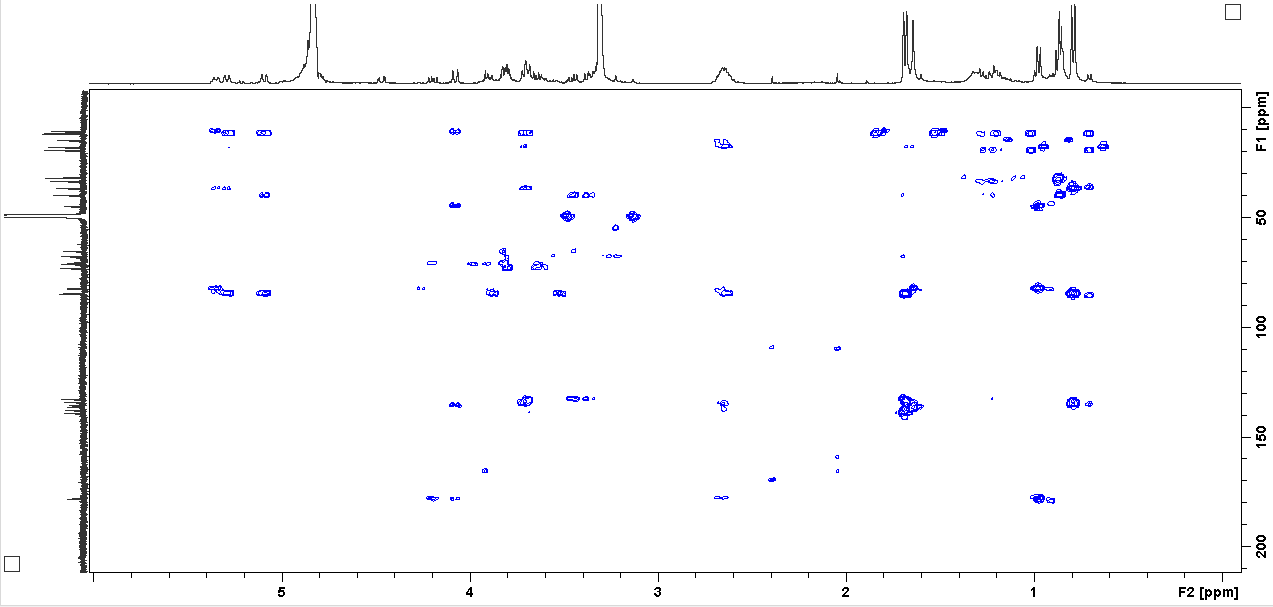


**Figure S21.** HMBC spectrum (CD_3_OD) of muyocopronol A (**3**).


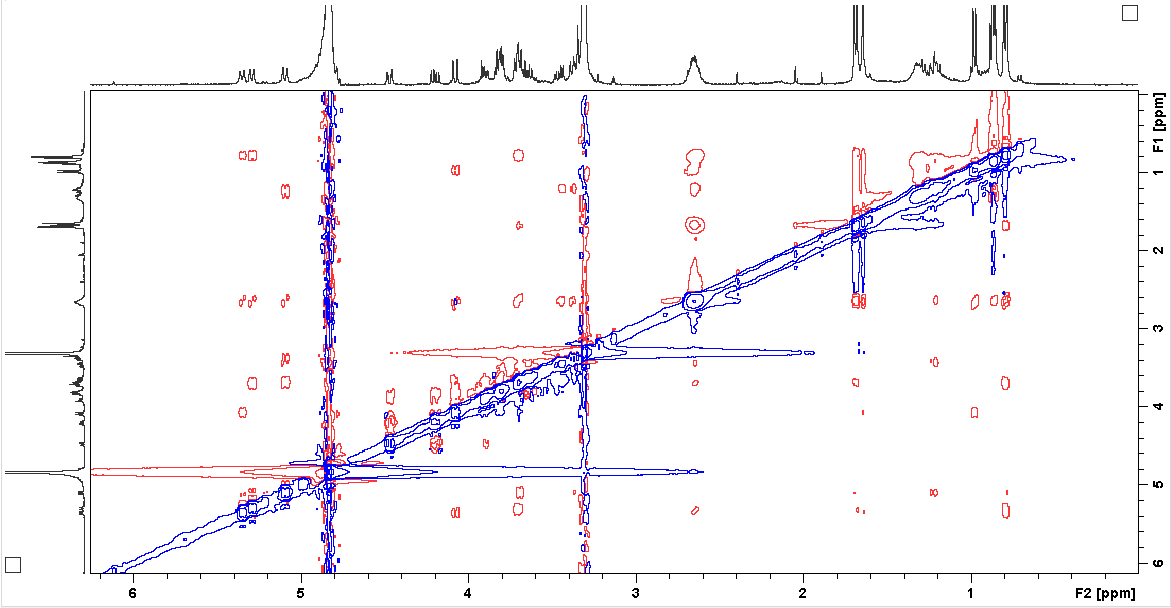


**Figure S22.** NOESY spectrum (CD_3_OD) of muyocopronol A (**3**).


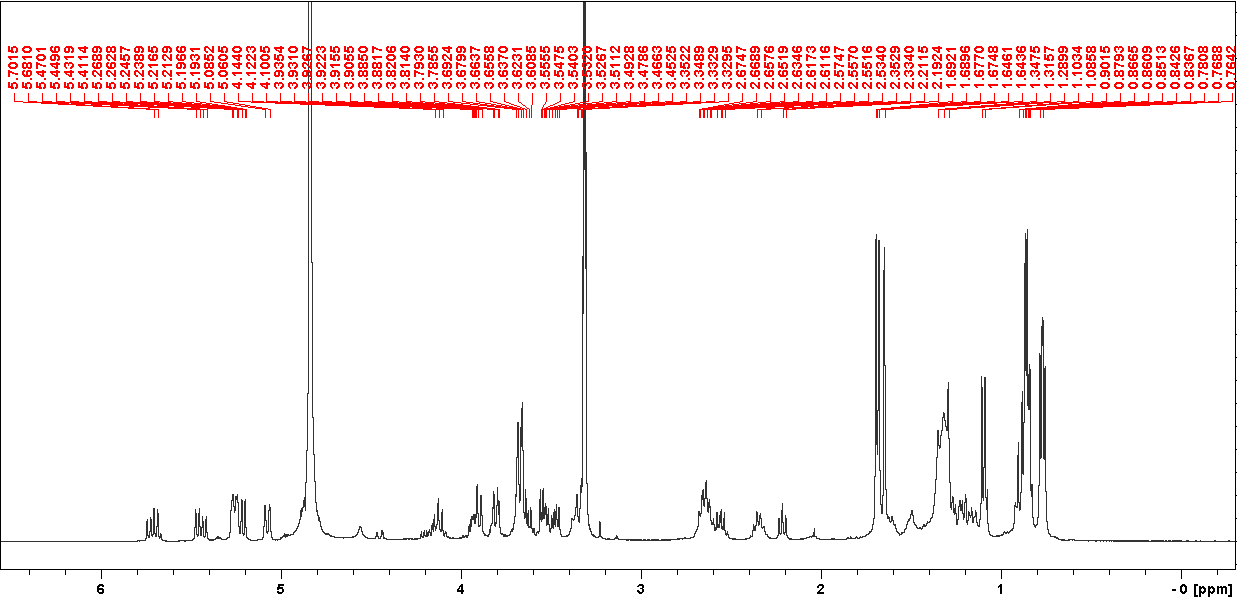


**Figure S23.** ^1^H NMR spectrum (CD_3_OD, 400 MHz) of muyocopronol B (**4**).


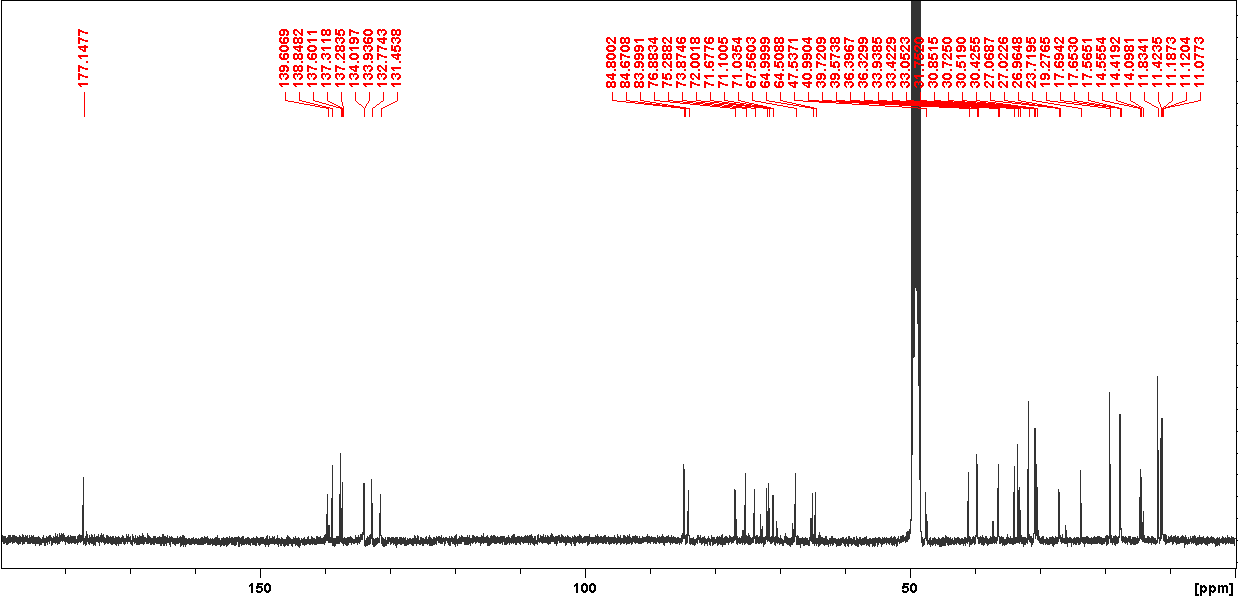


**Figure S24.** ^13^C NMR spectrum (CD_3_OD 100 MHz) muyocopronol B (**4**).


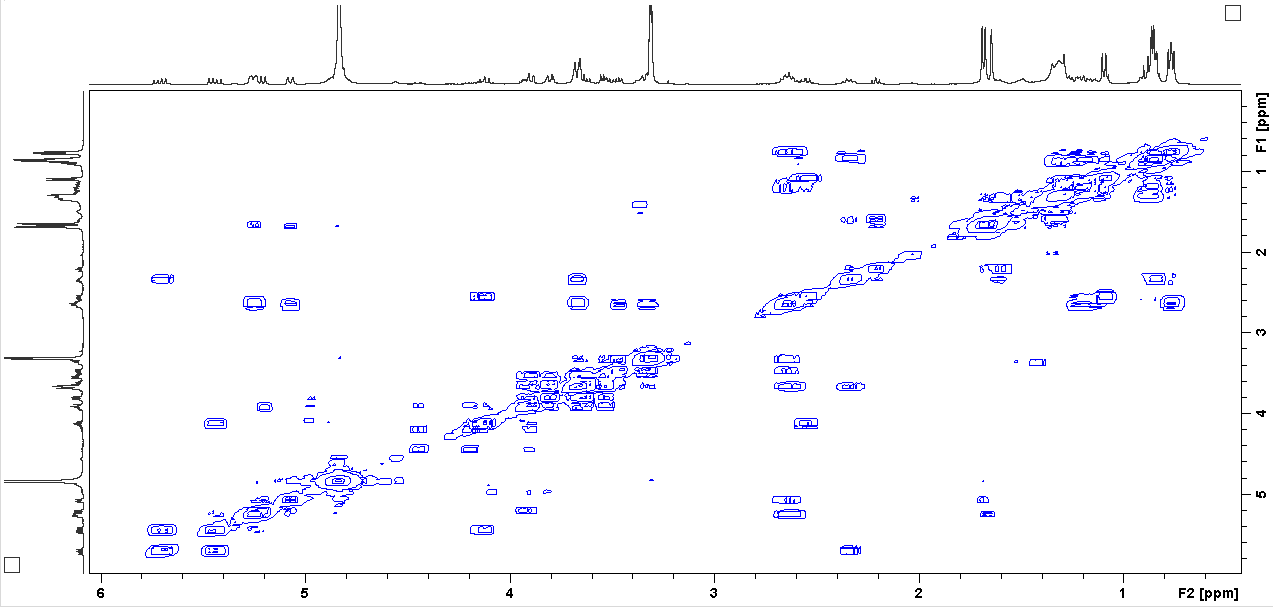


**Figure S25.** COSY spectrum (CD_3_OD) of muyocopronol B (**4**).


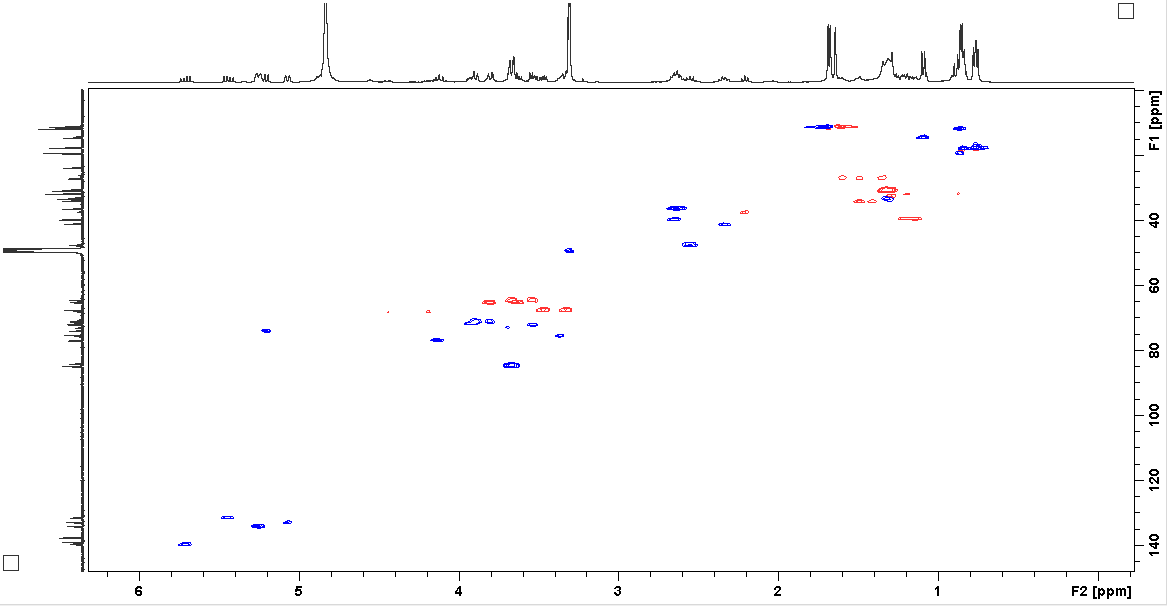


**Figure S26.** HSQC spectrum (CD_3_OD) of muyocopronol B (**4**).


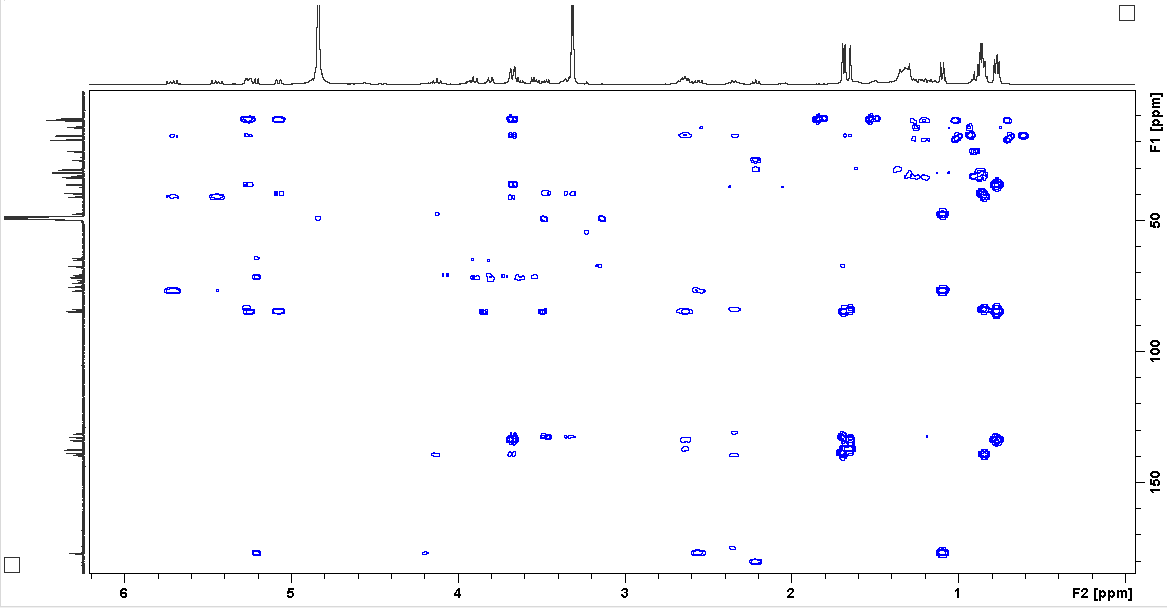


**Figure S27.** HMBC spectrum (CD_3_OD) of muyocopronol B (**4**).


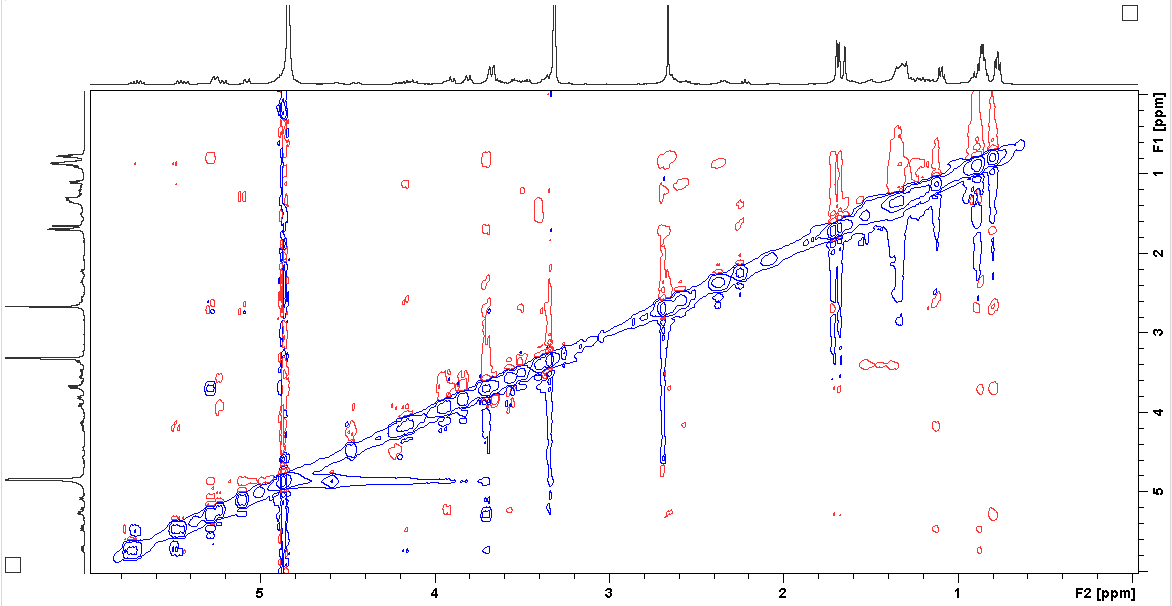


**Figure S28.** NOESY spectrum (CD_3_OD) of muyocopronol B (**4**).


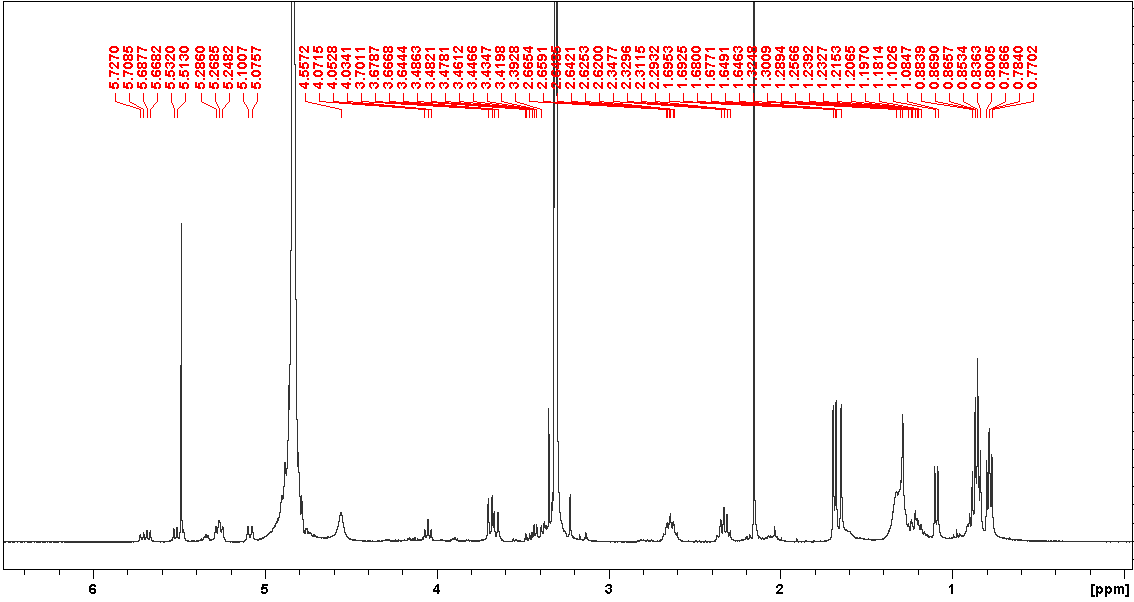


**Figure S29.** ^1^H NMR spectrum (CD_3_OD, 400 MHz) of muyocopronol C (**5**).


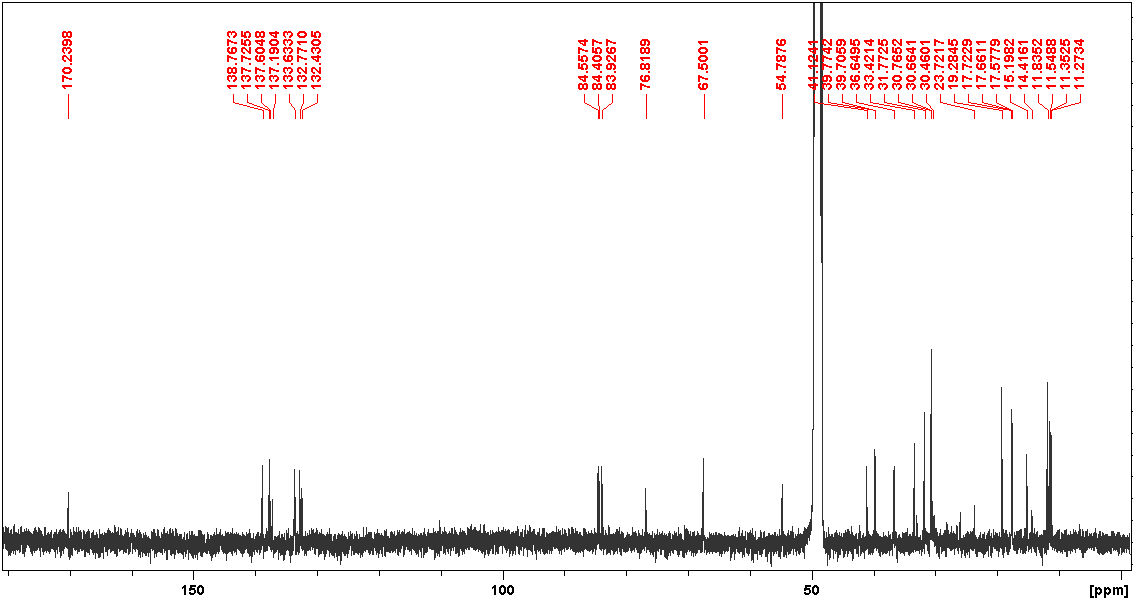


**Figure S30.** ^13^C NMR spectrum (CD_3_OD, 100 MHz) of muyocopronol C (**5**).


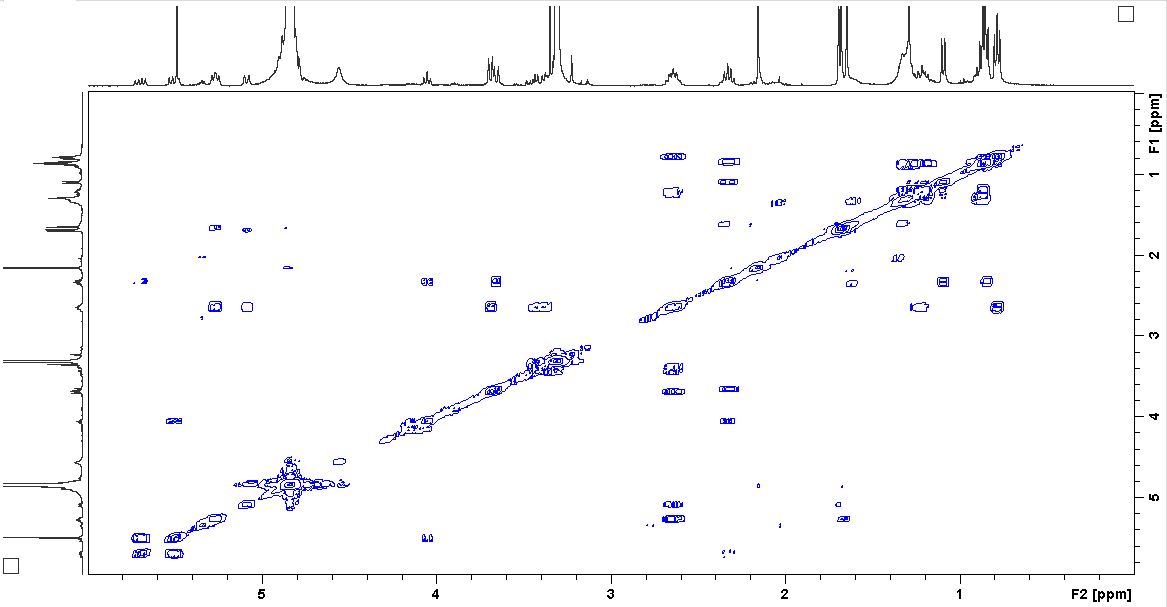


**Figure S31.** COSY spectrum (CD_3_OD) of muyocopronol C (**5**).


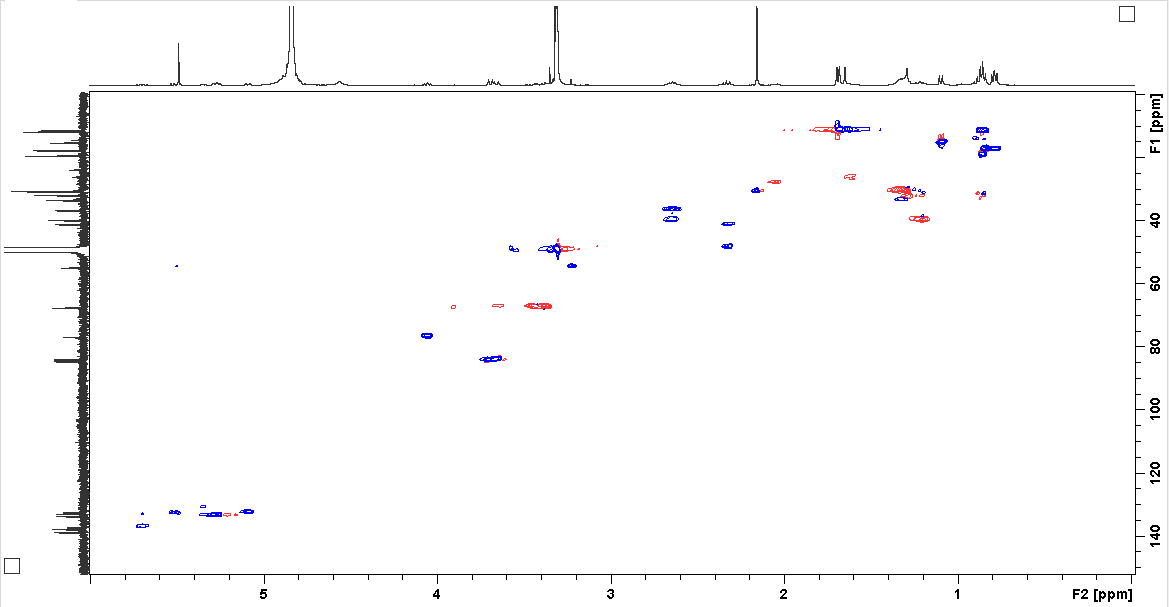


**Figure S32.** HSQC spectrum (CD_3_OD) of muyocopronol C (**5**).


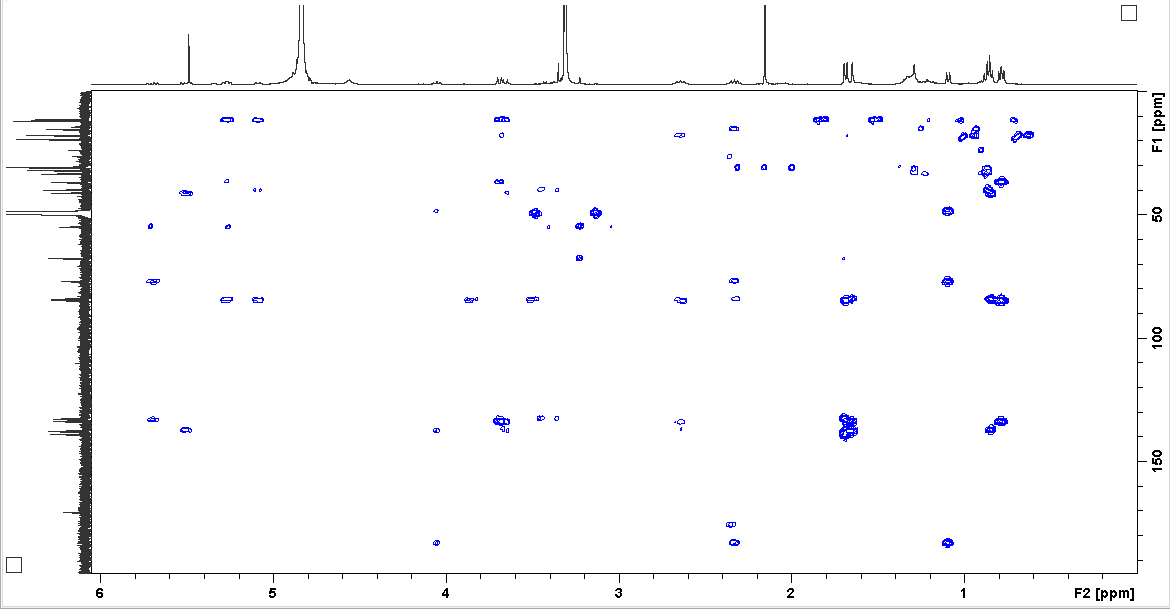


**Figure S33.** HMBC spectrum (CD_3_OD) of muyocopronol C (**5**).


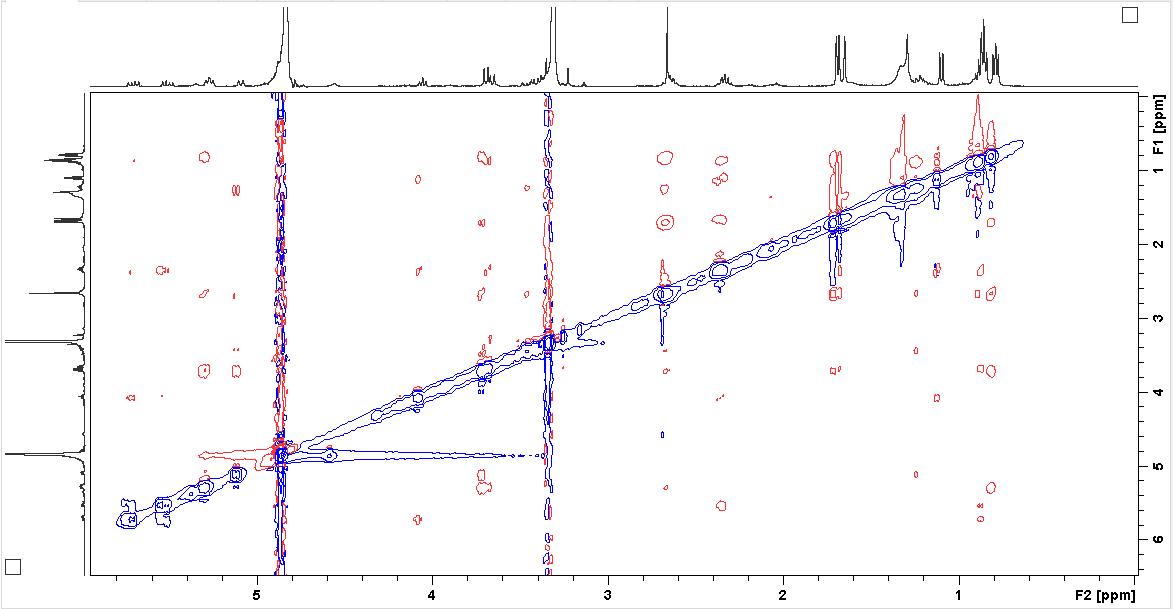


**Figure S34.** NOESY spectrum (CD_3_OD) of muyocopronol C (**5**).


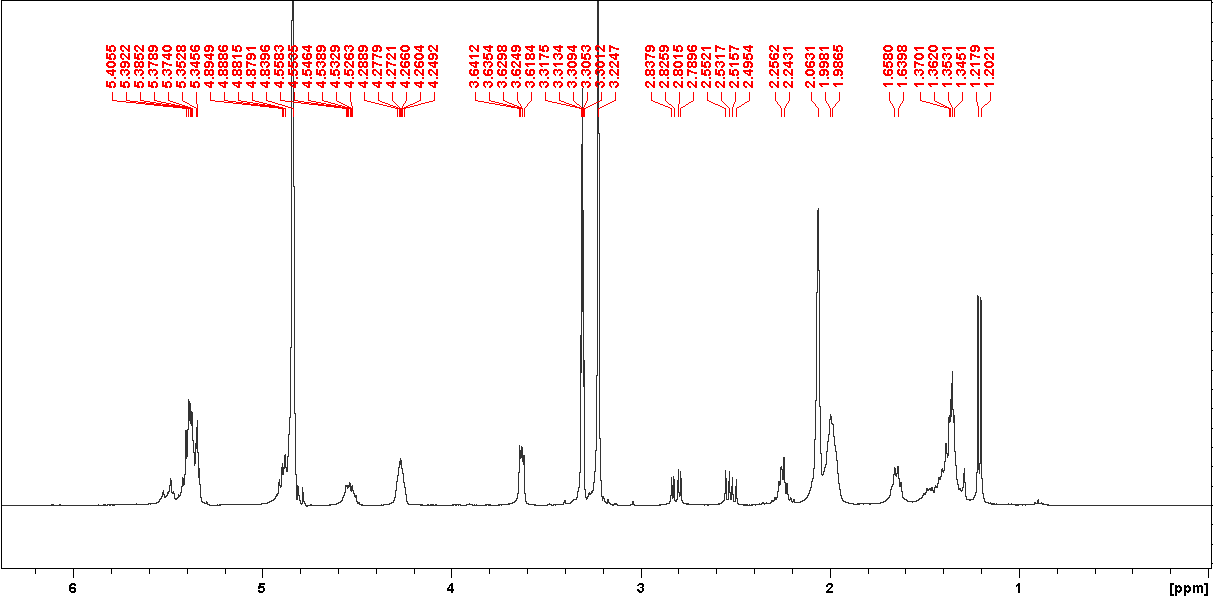


**Figure S35.** ^1^H NMR spectrum (CD_3_OD, 400 MHz) of tropicicolide (**6**).


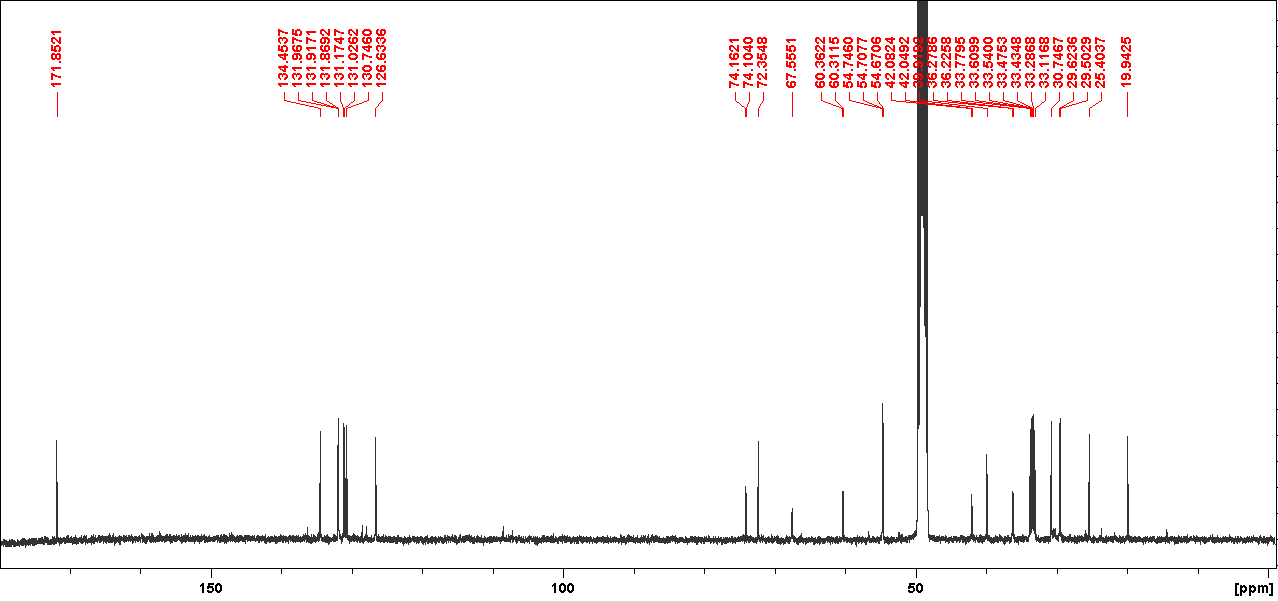


**Figure S36.** ^13^C NMR spectrum (CD_3_OD, 100 MHz) of tropicicolide (**6**).


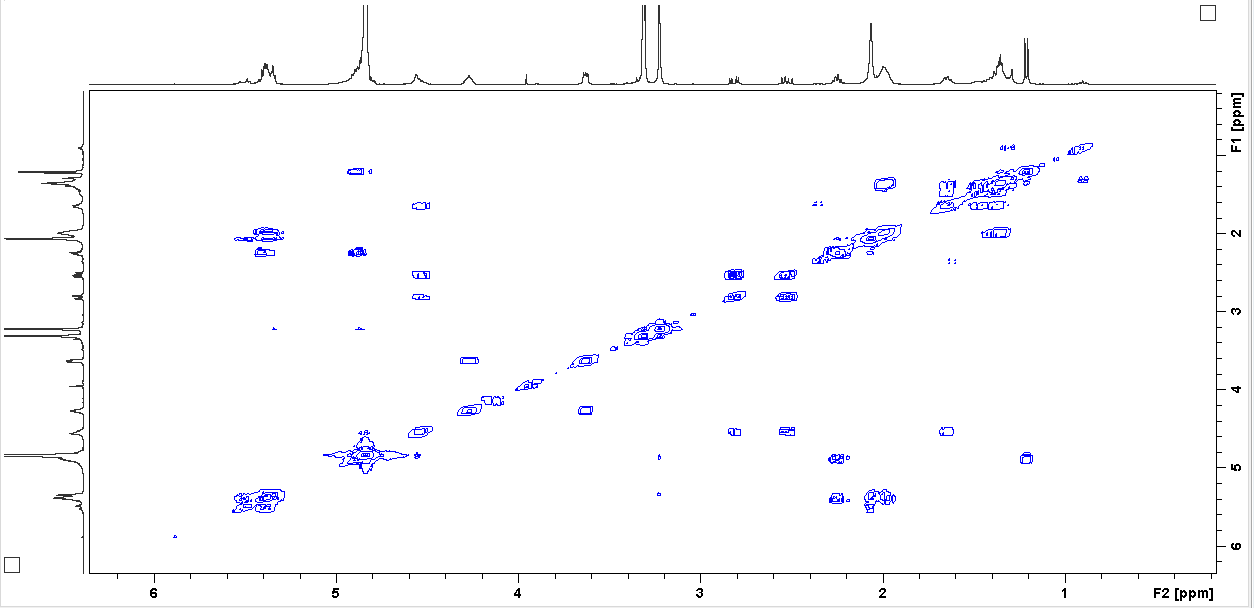


**Figure S37.** COSY spectrum (CD_3_OD) of tropicicolide (**6**).


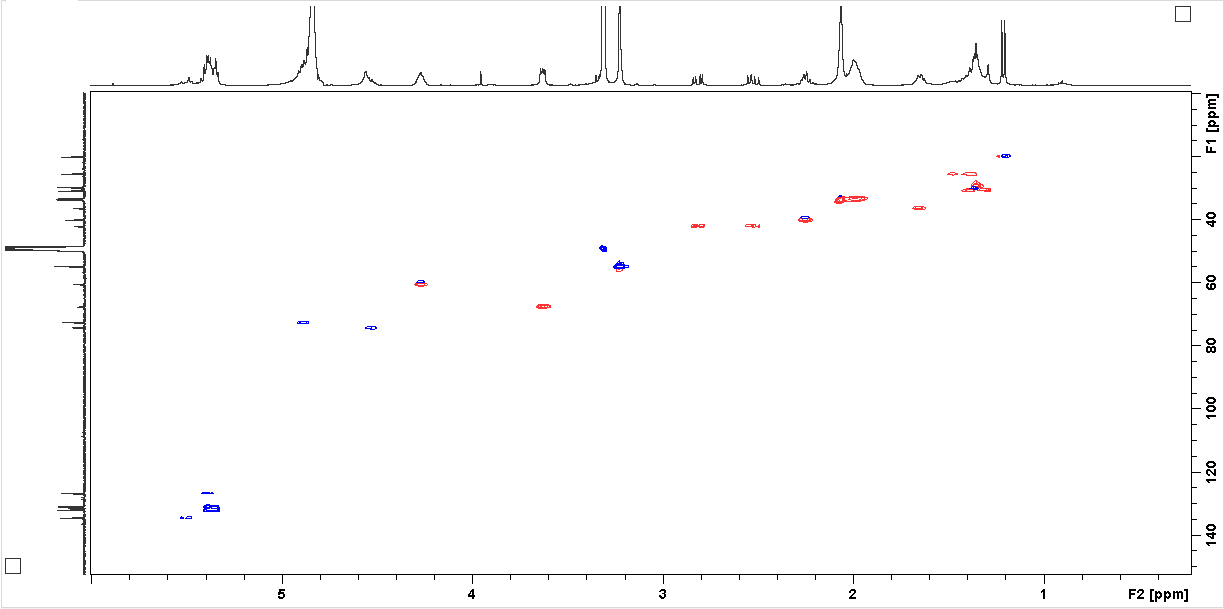


**Figure S38.** HSQC spectrum (CD_3_OD) of tropicicolide (**6**).


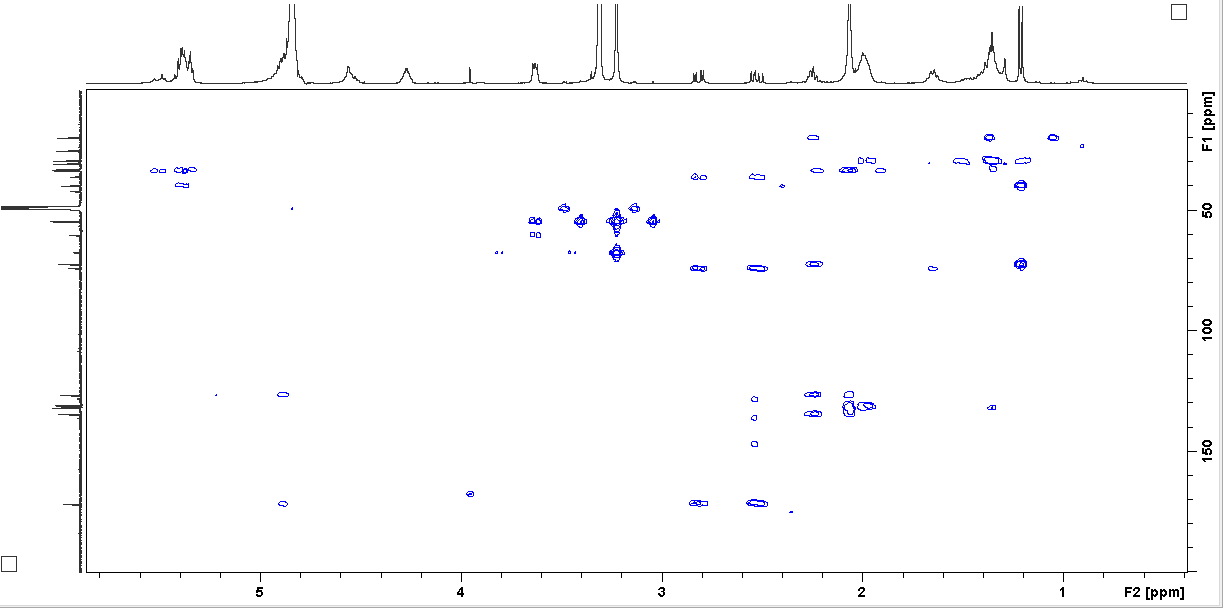


**Figure S39.** HMBC spectrum (CD_3_OD) of tropicicolide (**6**).


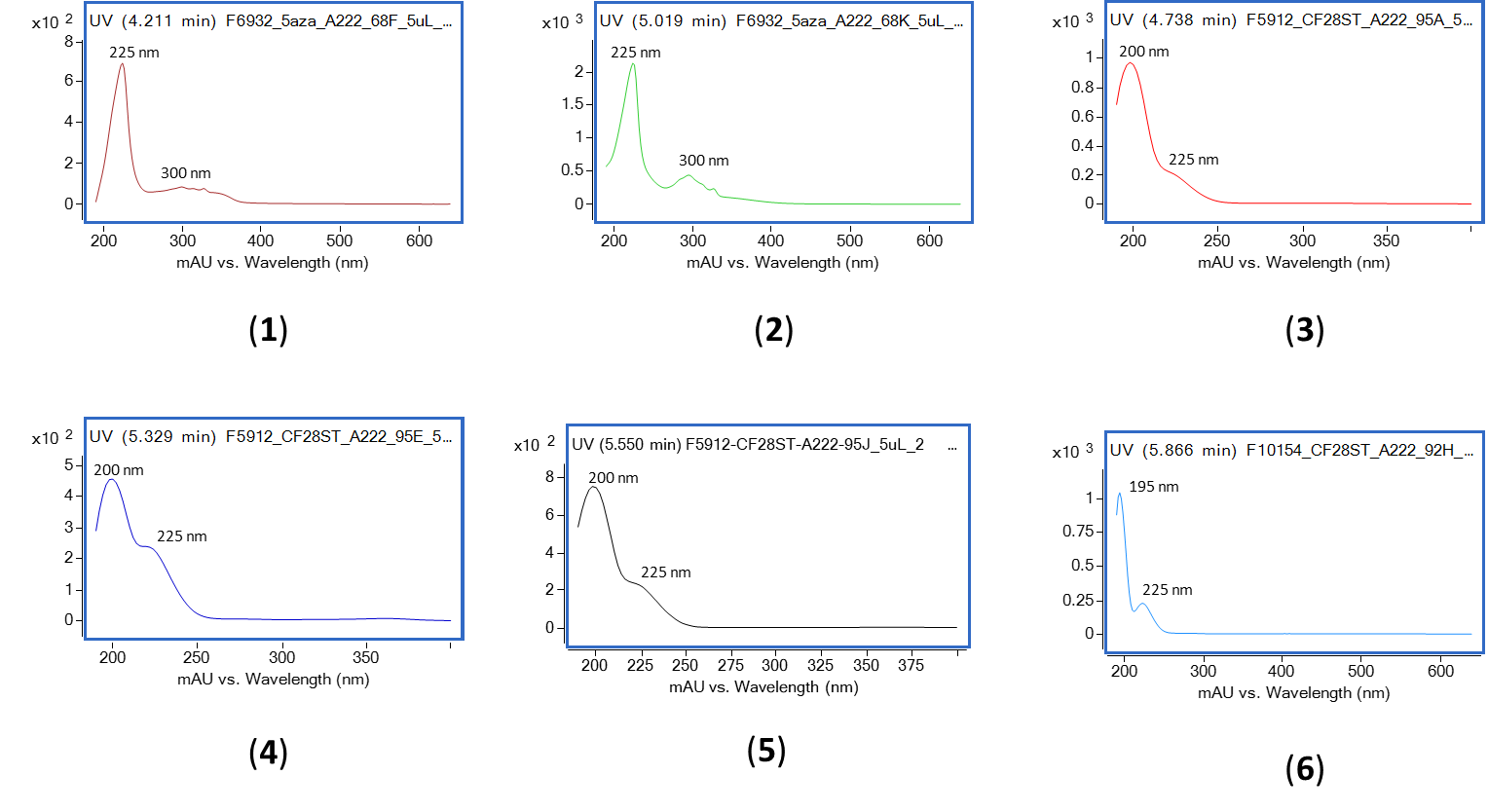


**Figure S40**. UV spectra of compounds **1**-**6**.


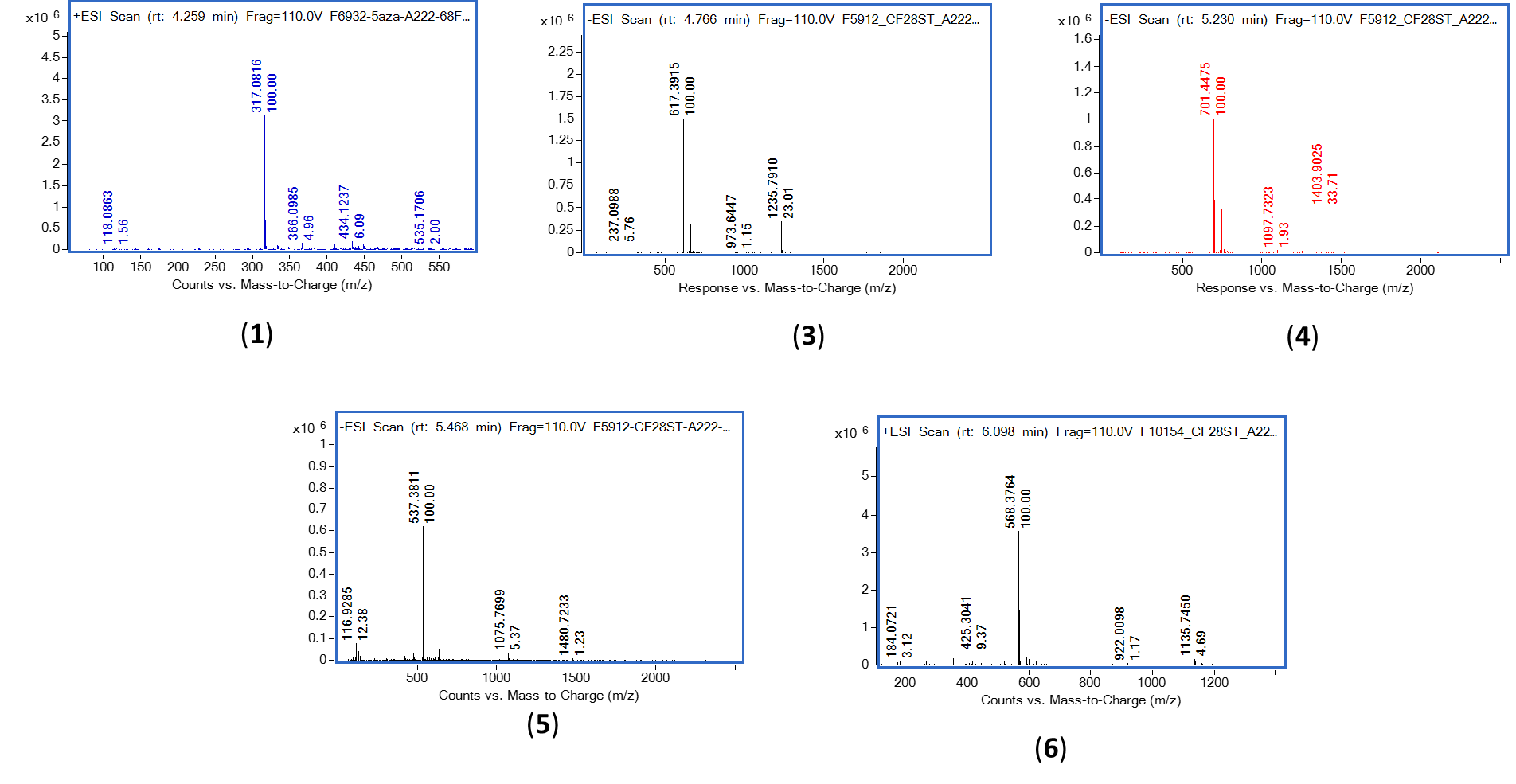


**Figure S41**. HRESIMS spectra of the new compounds **1** and **3 - 6**.


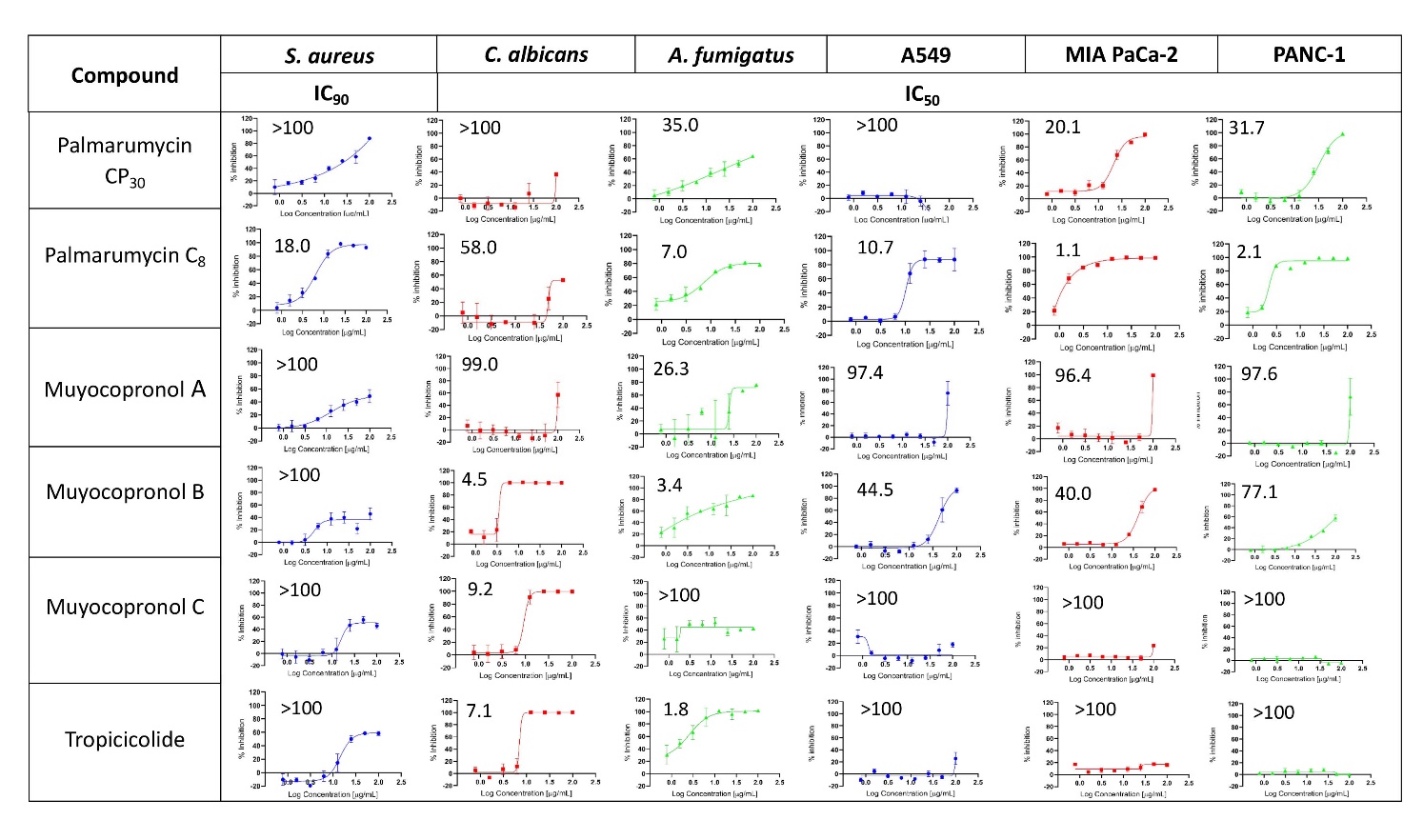


**Figure S42.** Dose-response curved for compounds **1- 6** against a panel of microbial pathogens and cancer cell lines. Values indicated within each of the curve represents IC_90_ (µg/mL) for S. aureus and IC_50_ (µg/mL) for the two fungal pathogens and the three cancer cell lines. The analysis for each sample was performed in triplicate.


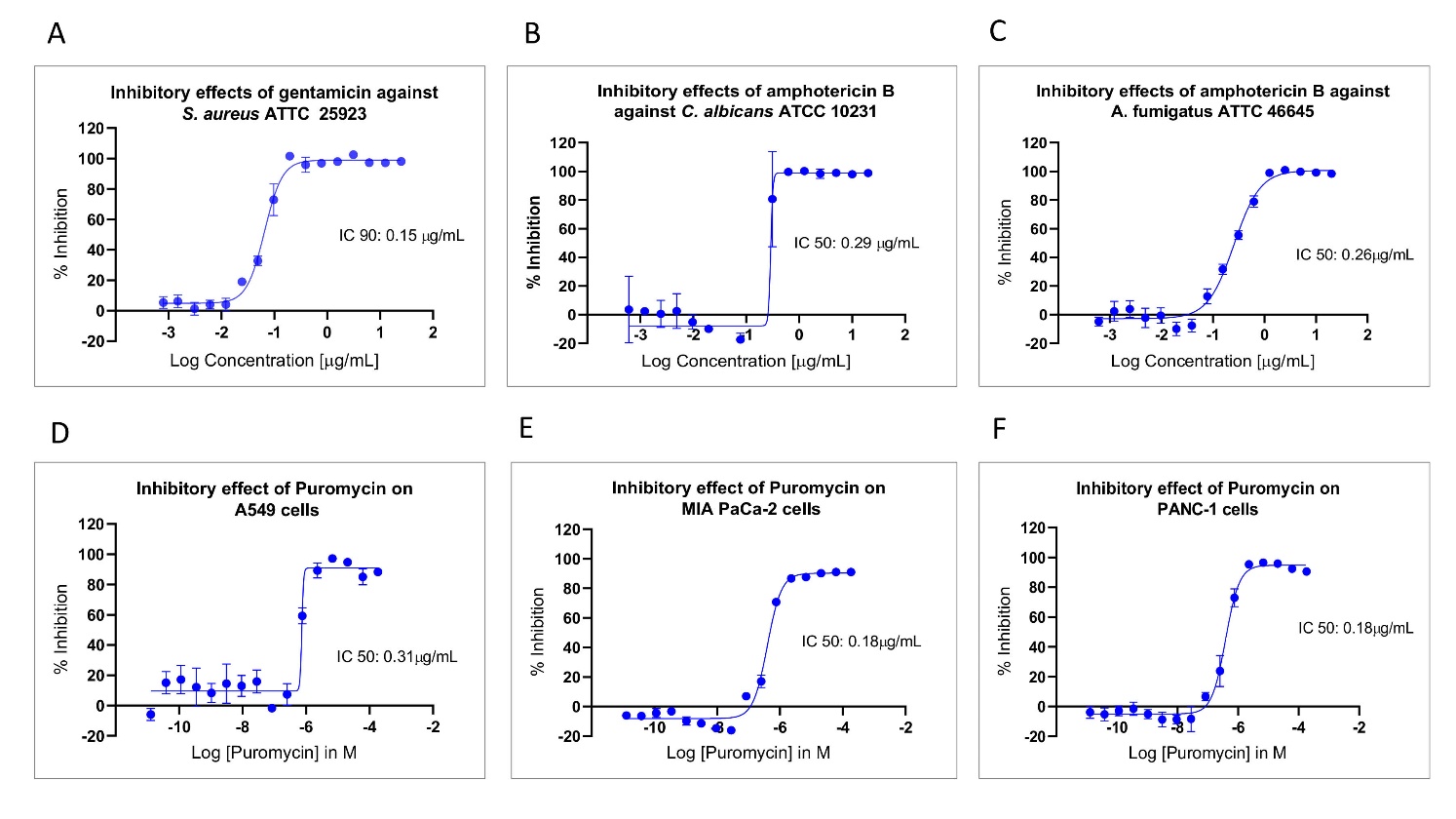


**Figure S43.** Inhibitory effects of standard antimicrobial agents; gentamicin and amphotericin B (**A-C**) and cytotoxic agent; puromycin (**D-F**) against the tested microbial pathogens and cell lines respectively.
